# Genetic Liability to Sedentary Behavior in Relation to Stroke, Its Subtypes and Neurodegenerative Diseases: A Mendelian Randomization Study

**DOI:** 10.3389/fnagi.2021.757388

**Published:** 2021-11-08

**Authors:** Fangkun Yang, Songzan Chen, Zihao Qu, Kai Wang, Xiaojie Xie, Hanbin Cui

**Affiliations:** ^1^Department of Cardiology, Ningbo Hospital of Zhejiang University (Ningbo First Hospital), Ningbo, China; ^2^School of Medicine, Zhejiang University, Hangzhou, China; ^3^Department of Cardiology, Second Affiliated Hospital, Zhejiang University School of Medicine, Hangzhou, China; ^4^Cardiology Center, Ningbo First Hospital, Ningbo University, Ningbo, China

**Keywords:** sedentary behavior, Mendelian randomization, stroke, television watching, neurodegenerative diseases

## Abstract

**Objective:** To investigate the causal association of domain-specific sedentary behaviors with cerebrovascular diseases and neurodegenerative diseases, and the potential mediators among these associations.

**Methods:** Genetic instruments were identified for television watching, computer use and driving behavior from a genome-wide association study including 408,815 subjects. Mendelian randomization (MR) analysis was used to estimate the causal effect of sedentary behaviors on the cerebrovascular diseases and neurodegenerative diseases. Multivariable MR analysis was applied to adjust potential confounding factors, and mediation analysis was conducted to explore potential mediators.

**Results:** Genetically predisposition to 1.5 h/day increase in leisure time watching television was associated with increased risk of all-cause stroke [odds ratio (OR) = 1.32, 95% confidence interval (CI) = 1.15–1.52, *p*-value for MR-Egger method (*P*_Egger_) = 0.11, *I*^2^ = 37%, Cochrane’s Q = 212, *p*-value for Cochran Q test (*P*_Q_) < 0.001], and ischemic stroke (OR = 1.28, 95%CI = 1.10–1.49, *P*_Egger_ = 0.04, *I*^2^ = 35%, Cochrane’s Q = 206, *P*_Q_ = 0.002). Interestingly, television watching may decrease the risk of Parkinson’s disease (OR = 0.65, 95%CI = 0.50–0.84, *P*_Egger_ = 0.47, *I*^2^ = 19%, Cochrane’s Q = 157, *P*_Q_ = 0.04). Television watching was a detrimental factor of cognitive performance (estimate = −0.46, 95%CI = −0.55 – −0.37, *P*_Egger_ = 0.001, *I*^2^ = 85%, Cochrane’s Q = 862, *P*_Q_ < 0.001). Sensitivity analyses using leave out method and MR-PRESSO method suggested weak evidence of pleiotropy.

**Conclusion:** We provided genetic evidence for the causal association of television watching with increased risk of all-cause stroke and ischemic stroke, decreased risk of Parkinson’s disease, and worse cognitive performance. The results should be interpreted with caution considering the pleiotropy.

## Introduction

Recently, prolonged time spent on sedentary behaviors has been suggested to increase the risk of cardiovascular disease (Young et al., 2016; Lavie et al., 2019). However, the evidence for association of leisure sedentary behaviors with cerebrovascular diseases (Chomistek et al., 2013; Cumming et al., 2019) and neurodegenerative diseases (Veldhuijzen van Zanten et al., 2016; Mattson and Arumugam, 2018; Younan, 2018; Ellingson et al., 2019) remains inconclusive. Whether this association is causal remains unclear. Elucidating the role of domain-specific sedentary behaviors in stroke subtypes and neurodegenerative diseases may help to provide a simple preventive approach to further mitigate stroke and neurodegenerative diseases for older people, which represent substantial burden for public health with significant mortality and disability (GBD 2016 Neurology Collaborators, 2019).

Mendelian randomization (MR) is an instrumental variable (IV) analysis approach that can be used to strengthen the causal inference in observational epidemiological studies (Lawlor et al., 2008). In brief, MR analysis utilizes single nucleotide polymorphisms (SNPs) associated with exposure of interest as IVs to determine whether the association between exposure and outcome reflects a causal relationship (Lawlor et al., 2008). Since SNPs are randomly assigned at conception, MR analysis is less vulnerable to confounding bias. In addition, the genotype is not modified by the phenotype, thus MR analysis can avoid the bias of reverse causality (Lawlor et al., 2008).

Leveraging the most updated genome-wide association study (GWAS) data for domain-specific sedentary behaviors, stroke and its subtypes including ischemic stroke (IS) and intracerebral hemorrhage (ICH), neurodegenerative diseases such as Alzheimer’s disease (AD), Parkinson’s disease (PD), Multiple sclerosis (MS) and cognitive performance (CP), and additional confounding traits, we aim to: (i) investigate the association of domain-specific sedentary behaviors with stroke and its subtypes and neurodegenerative diseases; (ii) determine the potential mediators of these associations and clarify the proportions mediated.

## Methods

### Genetic Instrument for Domain-Specific Sedentary Behavior

**FIGURE 1:**
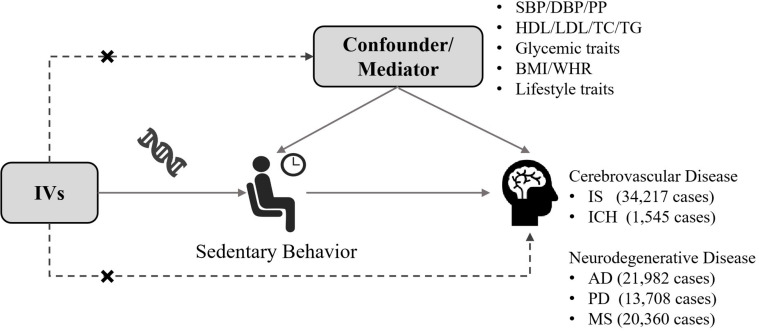
Conceptual schematic of the two-sample Mendelian randomization for the association between sedentary behavior and the risks of cerebrovascular diseases and neurodegenerative diseases. IV, instrumental variable; IS, ischemic stroke; ICH, intracerebral hemorrhage; AD, Alzheimer’s disease; PD, Parkinson’s disease; MS, multiple sclerosis; SBP, systolic blood pressure; DBP, diastolic blood pressure; PP, pulse pressure; HDL, high-density lipoprotein; LDL, low-density lipoprotein; TC, total cholesterol; TG, triglyceride; BMI, body mass index; WHR, waist-to-hip ratio.

The diagram of this two-sample MR analysis was displayed in Figure 1. Instrumental variables for domain-specific sedentary behaviors were identified from a GWAS of 408,815 European-descent subjects in the United Kingdom Biobank at a genome-wide significance threshold of *p* < 5 × 10^–8^. Three phenotypes of domain-specific sedentary behaviors were studied in that GWAS, including leisure television watching, computer use and driving behavior (van de Vegte et al., 2020). In the United Kingdom Biobank, 45.7% of the participants were male, and the average age was 57.4 [standard deviation (SD) 8.0] years old. The mean daily leisure television watching time was 2.8 (SD 1.5) hours, leisure computer use time was 1.0 (SD 1.2) hours and driving time was 0.9 (SD 1.0) hours, respectively (van de Vegte et al., 2020). The SNPs were identified at a genome-wide significance threshold of *p* < 5 × 10^–8^. Then, we performed linkage disequilibrium tests across SNPs based on the European 1000 Genomes Project reference panel (r^2^ < 0.01 and clump distance > 10,000 kb). If SNPs were in linkage disequilibrium, the SNP with greater *p* value would be removed. If a SNP was not available in the outcome dataset, we would search this SNP in an online tool named SNiPa^1^ based on European population genotype data and another SNP which was in linkage disequilibrium (r^2^ > 0.8) with such SNP would be identified as a proxy SNP. For palindromic SNPs, if the minor allele frequency is smaller than 0.42, then this SNP was regarded as inferable. The allele frequency of the effect allele gave us information whether the exposure effect allele and the outcome effect allele were on the same direction of strand. Any palindromic SNPs that had minor allele frequency larger than 0.42 (such as, rs17568389, rs870151, rs61331678, rs10786658, rs7043521, and rs62471080) were regarded as not inferable and we would discard these SNPs. The unit for the genetic estimates of exposures was 1 SD increase in leisure sedentary time. Specifically, it was about 1.5 h per day (h/day) for television watching, 1.2 h/day for computer use, and 1.0 h/day for driving. The overall F statistics was 5,747, 1,685, and 193 for the instrumental variables of television watching, computer use and driving, respectively (Supplementary Tables 1–3). The F-statistic of > 10 indicated a relatively low risk of weak instrument bias in MR analyses.

### Outcome Data Sources

The genetic data for stroke and neurodegenerative diseases were obtained from corresponding publicly available GWAS. Summary statistics for associations with all-cause stroke (AS) were obtained from the MEGASTROKE consortium, with up to 40,585 cases and 406,111 controls of European ancestry. Among all stroke cases, 34,217 were subclassified as IS, which were further divided into three subtypes: 4,373 for large-artery atherosclerotic stroke (LAS), 7,193 for cardioembolic stroke (CES) and 5,386 for small-vessel stroke (SVS) (Malik et al., 2018). Genetic data for ICH were obtained from International Stroke Genetics Consortium (ISGC), including a total of 1,545 cases and 1,481 controls of European ancestry. In that meta-analysis, ICH was further divided as lobar ICH (*n* = 664) and non-lobar ICH (*n* = 881) (Woo et al., 2014). In addition, genetic associations with white matter hyperintensity volume (WMHV) were obtained from an United Kingdom Biobank study (*n* = 8,448), as previously described, which was a biomarker of cerebral small vessel disease (Rutten-Jacobs et al., 2018). Summary statistics for associations with AD were obtained from the stage 1 GWAS meta-analysis implemented by the International Genomics of Alzheimer’s Project (IGAP), which consisted of 21,982 cases and 41,944 controls of non-Hispanic Whites (Kunkle et al., 2019). Genetic association data of PD were obtained from a large-scale meta-analysis of European ancestry, including a total of 13,708 cases and 95,282 controls (Nalls et al., 2014). The genetic data for MS were obtained from the previously published MS Chip conducted by the International Multiple Sclerosis Genetics Consortium (IMSGC), including 20,360 MS subjects and 19,047 controls of European ancestry [International Multiple Sclerosis Genetics Consortium [IMSGC], 2019]. Summary statistics for CP were derived from the Social Science Genetic Association Consortium (SSGAC), including 257,841 European-descent individuals (Lee et al., 2018). Data sources for associations with confounding risk factors, such as high-density lipoprotein (HDL) cholesterol, low-density lipoprotein (LDL) cholesterol, total cholesterol (TC), triglycerides, fasting glucose (FG), fasting insulin (FI), surrogate estimates of β-cell function (HOMA-B) and insulin resistance (HOMA-IR), HbA1c, modified Stumvoll insulin sensitivity index (ISI), systolic blood pressure (SBP), diastolic blood pressure (DBP), pulse pressure (PP), body mass index (BMI), waist-to-hip ratio (WHR), smoking, alcohol use, and physical activity were listed in Supplementary Table 4.

All studies included in the GWASs had been approved by relevant ethical review committees, and participants provided written informed consent. The current study only used summary-level data which was publicly available. Thus, additional ethical review for this study was not needed.

### Statistical Analysis

Our primary aim was to assess the associations of three phenotypes of domain-specific sedentary behaviors with stroke and its subtypes and four kinds of neurodegenerative disease traits. In secondary analyses, we investigated the mediation effect of the potential mediators. The statistical significance was defined as a *p* value of <1.3 × 10^–3^, corresponding to a Bonferroni correction of 39 independent tests (3 exposures and 13 outcomes). A *p* value between 1.3 × 10^––3^ and 0.05 was deemed suggestive evidence of possible associations. Statistical power was calculated using the online tool named mRnd^2^ (Freeman et al., 2013). The main parameters included sample size, Type-I error rate, proportion of cases, odds ratio (OR) of outcome, and proportion of variance explained for the association between the SNPs and the exposure variable (*r*^2^).

### Mendelian Randomization Analysis

First, the random-effects inverse-variance-weighted (IVW) method (Burgess et al., 2013) was used to estimate the causal effect of domain-specific sedentary behaviors on the outcomes. Wald estimate of each SNP was calculated based on summary statistics of its effect on sedentary behaviors and outcomes (Johnson, 2012). The relevant standard error was calculated using the Delta method (Johnson, 2012). The Wald estimates were meta-analyzed using the IVW method to generate the primary results (Burgess et al., 2016). In addition, the weighted-median method (Bowden et al., 2016), Maximum likelihood, MR-Egger method (Bowden et al., 2015), and MR Pleiotropy Residual Sum and Outlier (MR-PRESSO) method (Verbanck et al., 2018) were conducted in the follow-up sensitivity analyses. *I*^2^-index and Cochran’s Q statistics were calculated to test for heterogeneity produced by different genetic variants in the IVW analyses. An *I*^2^-index > 25% and Cochran’s Q *p* value of < 0.05 were considered as an indication of heterogeneity. The MR-Egger regression intercept test was used to assess the potential pleiotropy between the associations of exposure and outcome. A *p* value of < 0.05 indicated the present of pleiotropy. The MR-PRESSO method could detect outliers and provide causal estimates after the removal of identified outliers. Multivariable MR (MVMR) analysis (Burgess and Thompson, 2015) was applied to assess whether any association of domain-specific sedentary behaviors with strokes and neurodegenerative diseases could be affected by potential confounders, including lipids, glycaemic traits, blood pressure, body composition, tobacco and alcohol use, and physical activity. Moreover, MVMR analysis was also used to estimate the direct effect of sedentary behaviors on the outcomes not mediated *via* above factors.

### Mediation Analysis

For the mediation analysis, we used the difference method (Sanderson, 2020). First, IVW method was used to estimate the total effect of the domain-specific sedentary behaviors on the outcomes and MVMR method was used to estimate the direct effect of these sedentary behaviors on the outcomes conditional on mediator (or mediators). Then the difference between these estimates generated the indirect effect of the sedentary behaviors on the outcomes that acted *via* the corresponding mediator (or mediators) included in the MVMR. Finally, indirect effect was divided by total effect to estimate the proportion mediated, as previously done (Burgess et al., 2017). We performed the statistical analyses using R version 3.6.3 software (R Foundation for Statistical Computing) together with the R package TwoSampleMR^3^ and MR-PRESSO^4^.

## Results

### Domain-Specific Sedentary Behaviors and Cerebrovascular Diseases

The primary IVW analyses results for association of domain-specific sedentary behaviors with stroke and its subtypes were shown in Table 1 and Supplementary Table 5. Overall, prolonged genetically predicted television watching time was significantly associated with increased risk of AS [OR = 1.32, 95% confidence interval (CI) = 1.15–1.52, *p* = 1.1 × 10^–4^]. However, genetically predicted computer use and driving behavior were not associated with the risk of AS. Subgroup analyses showed that television watching was significantly associated with the risk of IS (OR = 1.28, CI = 1.10–1.49, *p* = 1.2 × 10^–3^), suggestively associated with the risk of LAS, SVS, and ICH, but not associated with the risk of CES, lobar ICH, non-lobar ICH, and WMHV. Computer use was suggestively associated with decreased risk of ICH and non-lobar ICH, but not associated with other stroke subtypes. Driving behavior was not associated with any stroke subtype.

**TABLE 1:** Mendelian randomization association of genetically determined sedentary behaviors with cerebrovascular disease traits and neurodegenerative disease traits.

Outcome	Television watching					Computer use				
	OR/beta (95%CI)	*p* value	*I* ^2^	*P*_Q_	*P*_intercept_	OR/beta (95%CI)	*p* value	*I* ^2^	*P*_Q_	*P*_intercept_
**Cerebrovascular disease traits**										
AS	1.32(1.15,1.52)	**1.1E-04**	37.3	<0.001	0.14	0.88(0.71,1.10)	0.27	19.6	0.13	0.66
AIS	1.28(1.10,1.49)	**1.2E-03**	35.4	0.002	0.20	0.94(0.74,1.19)	0.62	19.1	0.13	0.56
CES	0.99(0.78,1.25)	0.92	24.1	0.003	0.44	1.30(0.82,2.06)	0.26	21.9	0.10	0.23
LAS	1.48(1.09,1.99)	0.01	20.1	0.01	0.12	0.84(0.48,1.48)	0.55	11.2	0.26	0.44
SVS	1.66(1.21,2.27)	1.8E-03	27.2	0.003	0.89	0.73(0.44,1.20)	0.22	4.8	0.38	0.46
ICH	1.97(1.03,3.75)	0.04	0	0.52	0.80	0.26(0.08,0.86)	0.03	0	0.99	0.75
Non-lobar ICH	1.61(0.75,3.49)	0.22	0	0.95	0.61	0.16(0.04,0.67)	0.01	0	0.99	0.84
Lobar ICH	2.25(0.93,5.40)	0.07	8.6	0.18	0.61	0.45(0.08,2.37)	0.35	0	0.79	0.42
WMH	0.12(−0.02,0.27)	0.10	12.4	0.09	0.91	0.29(−0.06,0.64)	0.10	43.8	0.001	0.44
**Neurodegenerative disease traits**										
AD	1.15(0.97,1.36)	0.11	16.6	0.03	0.06	0.67(0.48,0.92)	0.01	14.2	0.21	0.99
PD	0.65(0.50,0.84)	**1.3E-03**	18.5	0.04	0.13	0.98(0.62,1.55)	0.92	12.0	0.24	0.06
MS	1.00(0.99,1.00)	0.84	33.6	0.02	0.72	1.00(1.00,1.01)	0.90	33.9	0.01	0.78
CP	−0.46(−0.55,−0.37)	**1.2E-24**	84.8	<0.001	0.28	0.65(0.50,0.80)	**4.0E-17**	82.5	<0.001	0.14

*beta, the Mendelian randomization effect of continuous variable outcome, such as white matter hyperintensity and cognitive performance.*

*OR, odds ratio; CI, confidence interval; *P*_Q_, *p* value corresponding to Cochran Q test; *P*_intercept_, *p* value corresponding to MR-Egger intercept test; AS, all-cause stroke; AIS, all-cause ischemic stroke; CES, cardioembolic stroke; LAS, large-artery atherosclerotic stroke; SVS, small vessel stroke; ICH, intracerebral hemorrhage; WMH, white matter hyperintensity; AD, Alzheimer’s disease; PD, Parkinson’s disease; MS, multiple sclerosis; CP, cognitive performance. Bold value indicates a *p* value of < 1.3 × 10^−3^.*

### Domain-Specific Sedentary Behaviors and Neurodegenerative Diseases

The associations between domain-specific sedentary behaviors and neurodegenerative diseases estimated using the IVW method were demonstrated in Table 1. Genetically predicted television watching was significantly associated with decreased risk of PD (OR = 0.65, CI = 0.50–0.84, *p* = 1.3 × 10^–3^) and worse CP (estimate = −0.46, CI = −0.55 – −0.37, *p* = 1.2 × 10^–24^), but not associated with the risk of AD or MS. Computer use was significantly associated with better CP (estimate = 0.65, CI = 0.50–0.80, *p* = 4.0 × 10^–17^), suggestively associated with decreased risk of AD, but not associated with the risk of PD or MS. Driving behavior was suggestively associated with worse CP, but not associated with the risk of AD, PD, or MS.

### Sensitivity Analyses

**FIGURE 2:**
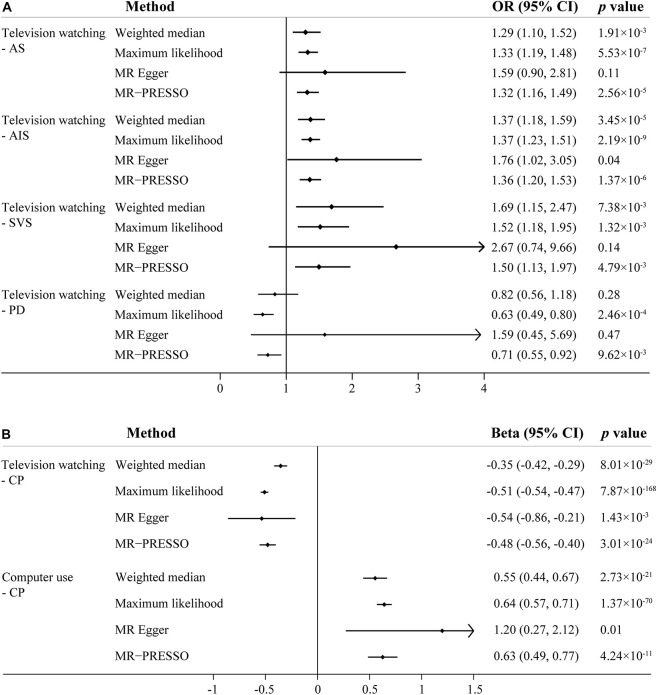
Sensitivity analysis of sedentary behaviors with stroke, Parkinson’s disease and cognitive performance using different Mendelian randomization statistical models. **(A)** The effect of television watching on all-cause stroke (AS), any ischemic stroke (AIS) and Parkinson’s disease (PD); **(B)** The effect of television watching and computer use on cognitive performance (CP). OR, odds ratio; CI, confidence interval.

Given concern that pleiotropy may contribute to an inflation of association statistics, we turned to additional MR approaches (weighted median, Maximum likelihood, MR-Egger, MR-PRESSO) to reanalyze the significant associations observed in primary IVW analyses (Figure 2). In line with the primary result, television watching was significantly or suggestively associated with increased risk of AS and IS in sensitivity analyses except the MR-Egger analysis. Television watching was significantly associated with decreased risk of PD in Maximum likelihood and MR-PRESSO analysis, but not in other analyses. Television watching was significantly or suggestively associated with worse CP in all of the sensitivity analyses. Similarly, computer use was significantly or suggestively associated with better CP in all of the sensitivity analyses. As shown in the Table 1, we found that the main MR analyses suffered from different degrees of heterogeneity. The random-effects IVW method was used as the main statistic method. Heterogeneity statistics provided important information on pleiotropy. Low heterogeneity usually indicated the absence of pleiotropic effects. However, no obvious pleiotropy was found using MR-Egger regression intercept tests (all the *p* value > 0.05). Several outliers were detected in the main analyses and the MR-PRESSO method provided causal estimates after the removal of identified outliers. We further re-analyzed these genetic associations using IVW method after the removal of the outlier SNPs as sensitivity analyses. The association patterns did not change and the degrees of heterogeneity were attenuated (Supplementary Table 6).

### Multivariable Mendelian Randomization and Mediation Analysis

**FIGURE 3:**
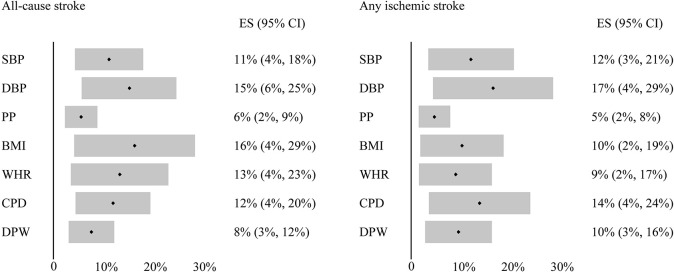
Mediation analysis of the effect of television watching on all-cause stroke and any ischemic stroke. Black dot indicates the average proportion, and gray bar indicates the 95% confidence intervals. ES, estimate for certain factor mediating the effect of television watching on stroke outcomes; CI, confidence interval; CPD, cigarettes per day; DPW, drinks per week.

Multivariable MR analysis was used to adjust for other traits and reevaluate the significant associations observed in our primary analysis, and to determine the potential mediators together with mediation analysis. The associations between domain-specific sedentary behaviors and confounding risk factors were demonstrated in Supplementary Table 7. After multivariable adjustment, the association between television watching and AS was still significant or suggestive though attenuated after adjusting for blood pressure, body composition, smoking and alcohol use, respectively (Table 2). Mediation analysis showed that this association was partially mediated by DBP (proportion mediated = 15%, 95%CI = 6–25%), BMI (proportion mediated = 16%, 95%CI = 4–29%), smoking (proportion mediated = 12%, 95%CI = 4–20%) and drinking (proportion mediated = 8%, 95%CI = 3–12%; Figure 3). Similar results were observed in analysis of association between television watching and AIS, which was also attenuated after adjusting for blood pressure, body composition, smoking and alcohol use (Supplementary Table 4). Mediation analysis showed that this association was partially mediated by DBP (proportion mediated = 17%, 95%CI = 4–29%), BMI (proportion mediated = 10%, 95%CI = 2–19%), smoking (proportion mediated = 14%, 95%CI = 4–24%) and drinking (proportion mediated = 10%, 95%CI = 3–16%; Figure 3). The association between television watching and PD was substantially attenuated when adjusted for BMI, and slightly attenuated after adjusting for smoking. Mediation analysis suggested that this association might be partially mediated by BMI (proportion mediated = 28%, 95%CI = −5–62%), and smoking (proportion mediated = 8%, 95%CI = 1–15%). The association between television watching and CP was still significant though attenuated after adjusting for HDL and WHR (Supplementary Table 8). This association was partially mediated by HDL (proportion mediated = 14%, 95%CI = 9–19%), and WHR (proportion mediated = 18%, 95%CI = 12–24%). The association between computer use and CP was consistent and not attenuated after adjustment for any above potential confounding factors (Supplementary Table 8).

**TABLE 2:** Multivariable Mendelian randomization of genetically determined television watching and stroke adjusted for confounding traits.

	All-cause stroke		Any ischemic stroke	
	OR (95%CI)	*p* value	OR (95%CI)	*p* value
**Adjusted for blood pressure traits**				
Systolic blood pressure	1.31 (1.15, 1.49)	3.2E-05	1.27 (1.12, 1.46)	3.5E-04
Diastolic blood pressure	1.29 (1.14, 1.47)	6.8E-05	1.26 (1.10, 1.44)	6.7E-04
Pulse pressure	1.33 (1.18, 1.51)	6.5E-06	1.30 (1.14, 1.48)	8.3E-05
**Adjusted for serum lipid traits**				
HDL-cholesterol	1.39 (1.15, 1.69)	8.8E-04	1.37 (1.12, 1.67)	2.0E-03
LDL-cholesterol	1.37 (1.15, 1.63)	3.9E-04	1.33 (1.11, 1.59)	1.6E-03
Total cholesterol	1.37 (1.15, 1.62)	3.7E-04	1.34 (1.12, 1.59)	1.3E-03
Triglyceride	1.35 (1.12, 1.63)	1.8E-03	1.32 (1.09, 1.60)	5.1E-03
**Adjusted for glycemic traits**				
Fasting glucose	1.35 (1.12, 1.62)	1.5E-03	1.33 (1.10, 1.61)	3.8E-03
Fasting insulin	1.40 (1.16, 1.69)	3.9E-04	1.36 (1.12, 1.65)	2.2E-03
HOMA-B	1.43 (1.20, 1.70)	6.2E-05	1.39 (1.16, 1.66)	4.3E-04
HOMA-IR	1.41 (1.16, 1.71)	6.0E-04	1.36 (1.11, 1.66)	3.1E-03
HbA1c	1.36 (1.14, 1.62)	5.2E-04	1.34 (1.12, 1.61)	1.4E-03
Insulin sensitivity index	1.35 (1.14, 1.60)	6.4E-04	1.33 (1.11, 1.59)	1.6E-03
**Adjusted for obesity traits**				
Body mass index	1.29 (1.10, 1.52)	2.0E-03	1.28 (1.08, 1.52)	4.1E-03
Waist-to-hip ratio	1.30 (1.10, 1.54)	1.7E-03	1.29 (1.08, 1.53)	4.4E-03
**Adjusted for lifestyle traits**				
Smoking	1.31 (1.15, 1.49)	6.9E-05	1.27 (1.10, 1.46)	8.1E-04
Alcohol use	1.33 (1.17, 1.51)	1.5E-05	1.28 (1.12, 1.47)	2.6E-04
Physical activity	1.39 (1.23, 1.57)	1.4E-07	1.36 (1.20, 1.54)	2.3E-06

*HOMA-B, homeostatic model assessment of β-cell function; HOMA-IR, homeostatic model assessment of insulin resistance; HDL-cholesterol, high-density lipoprotein cholesterol; LDL-cholesterol, low-density lipoprotein cholesterol; OR, odds ratio; CI, confidence interval.*

## Discussion

In current study, we investigated the association of domain-specific sedentary behaviors with cerebrovascular diseases and neurodegenerative diseases leveraging the GWAS summary data. Our study provided evidence for association of prolonged leisure television watching time with increased risk of AS and IS, and clarified a possible role for mediation through blood pressure, obesity, smoking and drinking. However, only suggestive or no evidence was observed for association of three phenotypes of sedentary behaviors with other stroke subtypes. In addition, our study suggested that increased television watching was associated with decreased risk of PD partially *via* the BMI and smoking, and with worse CP through the HDL and WHR in part. Our findings also supported the association between increased computer use and better CP. However, no significant evidence was observed for association between sedentary behaviors and other neurodegenerative diseases.

In line with these findings, previous studies also suggested that prolonged television watching time was a risk for AS and IS. A large prospective cohort study with 22,257 participants and median follow-up of 7.1 years identified a significant association of time spent watching television with all stroke (hazard ratio (HR) = 1.37, 95%CI: 1.10-1.71) and IS (HR = 1.35, 95%CI: 1.06-1.72) after adjusting for demographic factors (McDonnell et al., 2016). Several prior studies also found that leisure television watching might affect the outcome independent of physical inactivity (Ekelund et al., 2016; Carter et al., 2017). In addition, BMI, hypertension, tobacco and alcohol use has been reported as potential mediators for leisure sedentary behaviors (Ejsing et al., 2015; Grace et al., 2017; Bellettiere et al., 2019). Though hyperlipidemia and hyperglycaemia were also considered as covarying biomarkers for sedentary behaviors (Bellettiere et al., 2019), our findings suggested these two factors were not mediators. One prospective cohort study with 487,334 adults and median follow-up of 7.5 years indicated that physical activity was a protective factor for ICH (Bennett et al., 2017), but little was known about the relation between sedentary behavior and the ICH risk. Our study suggested no significant association between sedentary behaviors and the risk of ICH. As for the WMHV, our finding was supported by a prior large study, which found that sedentary time was only associated with WMHV in patients with reduced kidney function (Bronas et al., 2019). Few if any studies have explored the association of sedentary behaviors with IS subtypes and ICH subtypes. Suggestive evidence was observed in our study for some subtypes though not significant, further investigation was warranted to verify these potential associations.

Despite the evidence observed in conventional epidemiology studies for the association between sedentary behaviors and neurodegenerative diseases, this association might suffer from reverse causality or other confounding. For example, individuals with neurodegenerative diseases adopt a sedentary lifestyle. One cohort study suggested that compared with normal participants, individuals with AD spent larger proportion of time on leisure sedentary behavior and had more long-time sedentary bouts (Lu et al., 2018). Another cross-sectional study found that individuals with PD had longer sedentary bouts than that of controls, though the total time spent inactive was similar (Chastin et al., 2010). In addition, mobility disability was one of the major concerns in people with MS, who tended to engage in more sedentary behaviors and accumulate prolonged sedentary bouts (Ezeugwu et al., 2015). Therefore, it was difficult for observational studies to determine the causal direction of this association. The genetic determinants of cardiovascular diseases and neurodegenerative diseases have been increasingly well-characterized (Greuel et al., 2020; Hu et al., 2020, 2021a,b). In our MR study, we suggested an association between television watching with worse CP, which was consistent with previous studies (Falck et al., 2017; Stubbs et al., 2017). However, different from the prior studies, no evidence was observed for association of sedentary behaviors with AD or MS, and even protective effect of television watching on the risk of PD was observed in our study. The association between television watching and PD must be interpreted with caution, since it was not robust in sensitivity analysis and potential pleiotropy may exist. Further research was needed to explore this nature association. A possible explanation for the association between computer use and better CP observed in our study was that computer use might involve intellectual challenges to bolster brain function instead of merely watching the auto-playing video like television (Mattson, 2015). The underlying mechanism needed further investigation.

The major strength of current study was the MR design, which reduced the confounding and reverse causation bias. Multiple approaches were applied to estimate the effect of sedentary behavior on the cerebrovascular diseases and neurodegenerative diseases, though the estimates calculated by the MR-Egger method were imprecise (statistically inefficient). MVMR and mediation analysis were used to determine potential mediators. Another strength was the large sample sizes for both the exposures and the outcomes. This, together with the valid IVs for domain-specific sedentary behaviors, resulted in high precision of the results in our study.

There were potential limitations to this study. First, although robust results were observed using multiple MR methods, the pleiotropy could not be fully excluded, which was an established limitation of the MR analysis. Different degrees of heterogeneity were observed in the main analyses, indicating the presence of pleiotropic effects. Thus, the results of current study should be interpreted with caution. Though, MR-Egger regression intercept tests found little evidence for the pleiotropy. In this study, MVMR was also applied to limit the misleading inferences introduced by other traits. Second, the sample sizes for ICH and its subtypes were limited, which might lead to statistically insufficient for MR analysis. Third, only five SNPs were selected as IVs for driving behaviors, which also might lead to statistically insufficient. Thus, the results for association between driving behavior and outcomes might be imprecise and must be interpreted with caution. Fourth, since the research on mechanism for association between sedentary behavior and neurodegenerative diseases was rare, it was difficult to interpret the results of mediation analysis. Fifth, our study was restricted to individuals of European ancestry, as such it was unclear whether our findings could be extrapolated to other ancestral populations. Finally, the sample overlapping might lead to an inflated estimate in the two-sample MR analysis. We provided the information of cohorts used in the GWASs of main outcomes (Supplementary Tables 6–9). The samples of GWAS for WMHV were all from the United Kingdom Biobank study. Thus, the results of sedentary behavior and WMHV should be interpreted with caution, considering the bias due to sample overlap.

## Conclusion

Our study provided genetic evidence for the causal association of prolonged television watching time and increased risk of AS and IS, decreased risk of PD, and worse CP, as well as the causal association of prolonged computer use time with better CP. These findings have major clinical and public health implications as sedentary behaviors can easily be modified. However, the results should be interpreted with caution considering the difficulty in completely rule out pleiotropy.

## Data Availability Statement

The datasets presented in this study can be found in online repositories. The names of the repository/repositories and accession number(s) can be found in the article/Supplementary Material.

## Ethics Statement

Ethical review and approval was not required for the study on human participants in accordance with the local legislation and institutional requirements. The patients/participants provided their written informed consent to participate in this study.

## Author Contributions

FY, SC, and HC contributed to the study conception and design. FY and ZQ performed material preparation, data collection, and analysis. FY, SC, and KW wrote the first draft of the manuscript. KW, XX, and HC critically revised the manuscript. All authors interpreted the results in the study and gave final approval of the version to be published.

## Conflict of Interest

The authors declare that the research was conducted in the absence of any commercial or financial relationships that could be construed as a potential conflict of interest.

## Publisher’s Note

All claims expressed in this article are solely those of the authors and do not necessarily represent those of their affiliated organizations, or those of the publisher, the editors and the reviewers. Any product that may be evaluated in this article, or claim that may be made by its manufacturer, is not guaranteed or endorsed by the publisher.

## Footnotes

1
http://snipa.helmholtz-muenchen.de/snipa3/

2
https://shiny.cnsgenomics.com/mRnd/

3
https://github.com/MRCIEU/TwoSampleMR

4
https://github.com/rondolab/MR-PRESSO
