# Supplementary material for: Genetic Liability to Sedentary Behavior in Relation to Stroke, Its Subtypes and Neurodegenerative Diseases: A Mendelian Randomization Study
Source: Front Aging Neurosci. 2021 Nov 8;13:757388. doi: 10.3389/fnagi.2021.757388 (PMC8641575; doi:10.3389/fnagi.2021.757388)
Supplement: Supplementary file 1 [file Data_Sheet_1.pdf]

Supplementary Table 1. Genome-wide significant and independent single nucleotide polymorphisms that were used as instruments for television watching.

SNP	chr	position	effect_allele	other_allele	eaf	beta	se	*p* value	F-statistics
rs10041724	5	124273520	T	C	0.81	0.018	0.003	3.9E-11	41.2
rs10054327	5	147871137	G	A	0.58	0.017	0.002	3.4E-15	58.9
rs10145592	14	94287215	C	G	0.41	-0.015	0.002	1.8E-11	43.3
rs10189857	2	60713235	A	G	0.57	-0.021	0.002	6.2E-21	84.2
rs1022785	14	29571653	G	A	0.14	0.018	0.003	7.1E-09	31.9
rs10234444	7	41828527	G	A	0.82	0.016	0.003	2.9E-08	30.5
rs10246289	7	115544763	A	G	0.11	0.019	0.003	1.7E-08	29.8
rs1031423	5	93276883	T	C	0.22	-0.019	0.003	1.8E-12	48.0
rs10427502	21	40654840	G	A	0.62	0.014	0.002	8.6E-10	36.2
rs10737620	1	193057971	T	A	0.27	0.014	0.002	2.6E-09	33.4
rs10771746	12	30791864	C	T	0.72	-0.014	0.002	2.5E-09	33.7
rs10772643	12	13415288	C	T	0.11	0.025	0.004	1.3E-12	49.2
rs10876864	12	56401085	G	A	0.43	-0.013	0.002	1.0E-09	35.5
rs10890123	1	74802123	C	T	0.77	0.014	0.003	3.3E-08	29.2
rs10932837	2	221044785	C	T	0.49	-0.013	0.002	1.3E-09	35.1
rs10940659	5	59360341	A	G	0.53	-0.013	0.002	1.5E-08	32.9
rs10994943	10	63591413	T	G	0.58	0.013	0.002	3.1E-09	33.7
rs11020045	11	92487907	A	C	0.67	-0.013	0.002	1.6E-08	30.6
rs11130793	3	60884659	C	T	0.60	0.013	0.002	5.0E-09	32.7
rs111901094	19	19642795	G	T	0.82	-0.017	0.003	2.0E-09	35.6
rs11201422	10	86924483	T	C	0.67	0.013	0.002	3.4E-08	29.2
rs11218575	11	122154576	C	T	0.57	0.015	0.002	2.1E-12	47.5

rs11245482	10	126733546	T	C	0.61	-0.013	0.002	2.6E-09	33.9
rs114328297	1	91190854	T	G	0.78	0.014	0.003	4.4E-08	29.1
rs114600294	3	181419367	G	C	0.79	-0.016	0.003	7.9E-10	35.6
rs1156541	18	39952989	C	T	0.22	0.015	0.003	2.8E-08	29.5
rs11654952	17	4813799	T	G	0.86	-0.017	0.003	4.3E-08	29.1
rs11657730	17	79372489	C	T	0.64	0.013	0.002	1.1E-08	31.4
rs11689199	2	100820421	A	G	0.60	0.019	0.002	5.5E-17	67.2
rs11714337	3	71582521	G	A	0.57	0.014	0.002	4.7E-11	41.6
rs11763734	7	126400910	A	C	0.51	-0.013	0.002	8.2E-09	31.9
rs11810109	1	44186812	A	T	0.70	0.016	0.002	5.4E-12	45.6
rs12105701	2	41737200	C	T	0.40	-0.013	0.002	5.4E-09	32.7
rs12272012	11	84861286	G	C	0.96	-0.030	0.005	1.9E-08	27.5
rs12289262	11	12894758	C	T	0.73	-0.014	0.002	2.0E-08	30.2
rs1243182	10	21916728	C	T	0.69	-0.019	0.002	2.0E-15	60.5
rs12476388	2	116330983	C	T	0.71	0.013	0.002	2.1E-08	29.8
rs12491503	3	165706855	G	A	0.67	-0.014	0.002	5.5E-10	37.0
rs12541615	8	118870151	T	C	0.82	-0.018	0.003	3.9E-10	37.0
rs12554512	9	23352293	T	C	0.58	0.021	0.002	3.8E-21	85.4
rs12725114	1	62432641	G	A	0.80	0.015	0.003	1.6E-08	30.6
rs1278847	1	110037483	C	A	0.69	0.016	0.002	1.2E-11	44.2
rs13029509	2	215374209	G	A	0.52	-0.018	0.002	2.0E-17	68.4
rs13107325	4	103188709	C	T	0.93	-0.029	0.004	1.5E-12	45.4
rs138256022	3	11603090	C	T	0.96	-0.031	0.006	2.9E-08	30.8
rs141184308	9	86463339	A	G	0.98	0.043	0.008	2.6E-08	30.0
rs1421334	8	30865733	A	C	0.45	0.017	0.002	2.2E-15	60.6

rs142710267	16	72169262.00	T	G	0.65	0.016	0.002	1.1E-10	49.1
rs1451533	2	105466005	G	A	0.72	-0.016	0.002	6.7E-12	40.6
rs17207890	11	95490754	G	A	0.66	0.016	0.002	1.1E-16	45.2
rs17379561	1	98340139	A	T	0.86	-0.026	0.003	7.0E-10	64.0
rs17512836	18	53194961	T	C	0.97	0.042	0.007	3.1E-09	42.4
rs17727474	10	127182959	C	T	0.83	0.018	0.003	1.4E-13	35.7
rs17789218	6	100600097	T	C	0.76	0.019	0.003	8.6E-12	51.6
rs2034768	3	93986371	A	G	0.49	0.015	0.002	5.9E-09	44.2
rs2045147	10	56660437	A	G	0.45	0.013	0.002	1.5E-10	32.6
rs2073869	9	135763816	C	T	0.83	0.019	0.003	1.3E-08	39.9
rs2092829	22	31780514	G	A	0.71	0.014	0.002	1.1E-08	31.1
rs2164744	12	109872039	T	C	0.64	-0.013	0.002	4.6E-09	31.4
rs2173650	12	117525235	G	T	0.85	0.018	0.003	3.0E-09	33.0
rs2184364	6	143183596	A	G	0.78	0.016	0.003	6.8E-12	34.1
rs2447098	17	2277720	C	A	0.48	-0.015	0.002	5.0E-10	45.3
rs2460	15	53073084	G	A	0.74	-0.015	0.003	2.9E-10	36.8
rs2584597	17	61941284	T	C	0.66	0.015	0.002	2.9E-14	41.8
rs2616830	9	1721385	G	A	0.46	0.017	0.002	3.2E-15	55.3
rs262890	5	62930015	A	G	0.70	-0.019	0.002	1.5E-08	59.4
rs2717559	8	143882420	A	G	0.56	0.012	0.002	1.2E-08	31.0
rs2787374	9	103054951	T	C	0.41	0.013	0.002	2.7E-10	30.9
rs303753	18	21074922	G	A	0.65	-0.015	0.002	1.4E-09	39.1
rs34864022	9	22609110	A	G	0.93	-0.026	0.004	3.0E-08	37.1
rs35574015	16	15146061	T	C	0.71	-0.013	0.002	5.5E-16	29.3
rs374722	2	147839830	G	A	0.15	0.025	0.003	4.8E-12	62.6

rs3754970	2	162091836	T	C	0.50	-0.015	0.002	3.2E-33	46.0
rs3796386	3	49899795	G	A	0.57	-0.026	0.002	2.8E-09	137.6
rs405797	14	26972834	T	A	0.25	-0.015	0.003	7.3E-09	34.0
rs42210	5	166408788	G	C	0.29	-0.014	0.002	2.1E-08	32.5
rs4334769	4	130245233	G	T	0.47	0.012	0.002	6.7E-09	29.8
rs4382592	9	134870755	T	G	0.30	0.014	0.002	5.0E-10	32.2
rs4523073	6	67550288	A	G	0.59	-0.014	0.002	1.6E-13	37.7
rs4577309	2	191288833	A	G	0.47	0.016	0.002	2.9E-08	52.1
rs4675246	2	202864487	G	T	0.80	-0.015	0.003	4.7E-09	28.7
rs4775373	15	61476936	T	C	0.36	0.013	0.002	1.3E-12	32.8
rs4845364	1	154141908	A	G	0.50	-0.015	0.002	1.4E-08	47.9
rs4937842	11	133734542	G	C	0.63	-0.013	0.002	1.0E-09	31.2
rs4973576	2	233806771	C	A	0.30	-0.015	0.002	2.1E-09	36.1
rs55700114	20	43717080	G	A	0.71	-0.014	0.002	2.4E-09	34.4
rs55909997	1	107581039	G	A	0.65	-0.014	0.002	3.8E-10	34.4
rs56103247	20	62483184	C	T	0.94	0.030	0.005	3.4E-10	41.0
rs56858768	13	86511730	G	A	0.70	-0.015	0.002	4.3E-09	38.1
rs57585211	5	107197725	T	G	0.83	-0.017	0.003	3.1E-13	32.2
rs6131281	20	11891724	C	T	0.60	0.016	0.002	1.3E-09	50.9
rs6141814	20	31368960	C	A	0.61	-0.014	0.002	7.8E-10	35.5
rs62379379	5	141082015	G	T	0.93	-0.026	0.004	7.0E-10	36.0
rs62641636	2	68425427	A	G	0.69	0.014	0.002	1.8E-09	36.3
rs6472942	8	76827190	T	C	0.57	-0.013	0.002	2.2E-11	34.9
rs6673341	1	184655649	T	G	0.47	-0.015	0.002	7.9E-12	42.8
rs66852340	4	3311070	C	T	0.78	-0.018	0.003	1.7E-10	44.5

rs6721975	2	5832667	T	C	0.23	-0.017	0.003	1.7E-13	40.4
rs6797840	3	85656569	A	C	0.46	-0.016	0.002	4.9E-15	52.7
rs6825241	4	152543783	C	A	0.54	-0.017	0.002	1.1E-10	58.7
rs6850494	4	82291771	A	C	0.62	-0.014	0.002	8.5E-18	39.4
rs6905544	6	98411631	A	G	0.40	-0.019	0.002	1.0E-09	70.9
rs6973656	7	77422583	A	G	0.60	-0.014	0.002	3.5E-08	35.8
rs6996198	8	65463442	C	T	0.84	-0.016	0.003	2.4E-09	29.6
rs7089973	10	116569565	C	A	0.62	-0.013	0.002	2.3E-08	34.6
rs7157001	14	99749484	A	G	0.74	-0.014	0.003	6.3E-10	30.8
rs71658797	1	77967507	T	A	0.88	-0.020	0.003	8.2E-13	35.9
rs7184800	16	53509131	G	A	0.70	0.017	0.002	3.4E-11	48.5
rs7189927	16	28913787	T	C	0.36	0.015	0.002	3.4E-10	42.4
rs7248205	19	10770305	C	T	0.40	0.014	0.002	4.5E-08	37.9
rs72671494	8	93195457	T	C	0.86	-0.017	0.003	3.0E-12	29.5
rs72781699	2	24277709	G	A	0.80	-0.019	0.003	4.9E-09	45.8
rs72828890	5	167806836	C	T	0.87	0.019	0.003	2.7E-13	34.4
rs72834698	6	26176517	G	A	0.86	0.023	0.003	2.7E-12	50.7
rs749671	16	31088347	G	A	0.63	0.016	0.002	2.8E-11	47.0
rs7564130	2	50606642	T	C	0.64	-0.015	0.002	1.7E-10	42.4
rs7693082	4	159857819	G	C	0.30	0.015	0.002	2.7E-09	39.2
rs7693703	4	118357405	G	A	0.91	0.023	0.004	1.9E-11	34.8
rs7700107	4	17880416	A	C	0.86	-0.021	0.003	3.6E-09	43.8
rs7716447	5	88800331	A	G	0.64	-0.013	0.002	1.5E-08	33.8
rs77215114	4	45187658	A	T	0.93	0.024	0.004	1.1E-10	31.7
rs7834121	8	10770311	G	T	0.50	-0.014	0.002	8.8E-15	39.8

rs7991062	13	100713194	C	G	0.66	-0.018	0.002	7.3E-14	57.5
rs801733	11	65934549	A	C	0.64	0.017	0.002	3.4E-08	53.8
rs8043253	15	41503730	C	T	0.57	-0.012	0.002	5.3E-10	29.3
rs8756	12	66359752	C	A	0.48	-0.014	0.002	1.5E-09	37.2
rs9471333	6	40362023	C	T	0.45	0.013	0.002	4.3E-11	34.7
rs9563168	13	54247827	G	A	0.79	0.018	0.003	3.4E-10	42.0
rs9569734	13	58319476	A	G	0.84	0.019	0.003	9.3E-19	39.3
rs9718104	6	166170539	T	G	0.94	-0.041	0.005	1.5E-08	76.8
rs973734	7	89387578	C	A	0.15	0.017	0.003	3.3E-09	30.5
rs9834970	3	36856030	T	C	0.50	0.013	0.002	4.1E-11	33.5
rs984409	1	67020440	G	A	0.36	-0.015	0.002	3.9E-12	41.8
rs9867121	3	114631548	C	A	0.82	0.020	0.003	4.5E-11	45.9
rs9902312	17	65070304	T	C	0.68	0.015	0.002	3.3E-14	41.7
rs9964724	18	35159124	C	T	0.32	0.018	0.002	6.6E-12	55.1

SNP, single nucleotide polymorphisins; chr, chromosome; eaf, effect allele frequence; se, standard error.

Supplementary Table 2. Genome-wide significant and independent single nucleotide polymorphisms that were used as instruments for computer use.

SNP	chr	position	effect_allele	other_allele	eaf	beta	se	*p* value	F-statistics
rs10208088	2	221055873	C	T	0.42	0.0131	0.0022	4.4E-09	34.1
rs10754920	2	146456888	C	G	0.11	0.0202	0.0035	6.7E-09	33.3
rs113851275	9	98297220	G	A	0.89	-0.0209	0.0035	3.2E-09	34.6
rs11708955	3	49540114	T	C	0.69	-0.0157	0.0024	4.2E-11	42.7
rs11749912	5	88065628	A	G	0.42	0.0139	0.0022	4.9E-10	38.5
rs12145677	1	110023610	G	A	0.70	-0.0171	0.0024	1.2E-12	50.1
rs12603813	17	43196584	T	C	0.75	0.0144	0.0025	1.3E-08	32.1
rs12706626	7	124531370	G	A	0.62	-0.0129	0.0023	1.4E-08	32.1
rs13262595	8	143316970	A	G	0.44	-0.0157	0.0022	1.2E-12	49.7
rs136553	22	27255675	C	T	0.62	-0.0152	0.0023	2.3E-11	44.3
rs1448355	11	131286685	C	T	0.38	-0.0146	0.0023	1.3E-10	41.1
rs1469249	5	113837198	G	A	0.79	0.0156	0.0027	9.5E-09	33.2
rs162894	5	131611872	T	G	0.33	0.0134	0.0023	1.1E-08	32.6
rs166835	15	47716037	C	T	0.44	0.0131	0.0022	3.4E-09	34.6
rs198262	14	57283283	C	T	0.04	0.0300	0.0055	3.9E-08	30.6
rs2068625	4	159856739	T	C	0.30	-0.0158	0.0024	4.0E-11	43.1
rs206965	12	120856332	T	C	0.21	0.0156	0.0027	9.1E-09	32.6
rs2220599	5	7378854	C	G	0.63	-0.0161	0.0023	2.4E-12	49.2
rs2345941	7	133337635	A	G	0.55	0.0146	0.0022	4.0E-11	43.1
rs2734849	11	113270160	A	G	0.49	-0.0135	0.0022	8.2E-10	37.2
rs2748985	1	1853184	T	C	0.45	-0.0153	0.0022	4.1E-12	47.5
rs35933007	12	38929341	G	A	0.77	-0.0152	0.0026	9.2E-09	33.3

rs3730399	16	67229019	A	G	0.93	0.0247	0.0045	3.3E-08	30.4
rs3944151	7	126970005	A	G	0.28	0.0137	0.0025	3.1E-08	30.6
rs4592851	2	215364232	G	T	0.75	-0.0144	0.0025	1.4E-08	31.8
rs4702	15	91426560	G	A	0.44	0.0122	0.0022	3.8E-08	30.0
rs4977839	9	23355310	G	A	0.58	-0.0199	0.0022	4.7E-19	78.7
rs55772938	7	70025113	A	G	0.70	-0.0151	0.0024	3.9E-10	38.8
rs6129084	20	59828857	A	T	0.36	-0.0141	0.0023	9.3E-10	37.4
rs6498759	16	18020295	T	C	0.31	0.0133	0.0024	2.7E-08	30.9
rs66643547	17	79375315	C	T	0.65	-0.0154	0.0023	3.2E-11	44.3
rs6744254	2	155986195	C	T	0.47	-0.0159	0.0022	5.4E-13	51.5
rs6774533	3	62471086	C	T	0.29	-0.0149	0.0025	1.2E-09	37.7
rs6857629	4	39684977	G	A	0.76	0.0154	0.0026	1.9E-09	35.9
rs6935828	6	140811367	C	T	0.45	-0.0121	0.0022	4.3E-08	29.6
rs7020477	9	116827760	A	G	0.73	0.0142	0.0025	1.2E-08	32.1
rs7209653	17	19882084	T	C	0.70	0.0156	0.0024	9.1E-11	41.4
rs7288455	22	39966547	A	G	0.44	0.0131	0.0022	4.0E-09	34.5
rs73578186	9	126334485	C	T	0.68	0.0150	0.0024	2.3E-10	40.3
rs78082503	3	116556913	G	C	0.79	-0.0154	0.0027	1.1E-08	32.6
rs784256	18	53398626	G	A	0.19	0.0185	0.0028	6.2E-11	42.7
rs9372625	6	98344031	G	A	0.62	-0.0184	0.0023	5.7E-16	65.3
rs9477970	6	19082663	T	A	0.81	-0.0160	0.0028	1.0E-08	32.4

SNP, single nucleotide polymorphisins; chr, chromosome; eaf, effect allele frequence; se, standard error.

Supplementary Table 3. Genome-wide significant and independent single nucleotide polymorphisms that were used as instruments for driving.

SNP	chr	position	effect_allele	other_allele	eaf	beta	se	*p* value	F-statistics
rs10186876	2	44241155	A	G	0.36	0.0144	0.0023	7.2E-10	39.1
rs1198575	1	98562260	T	C	0.19	0.0190	0.0028	2.0E-11	46.0
rs4765541	12	124465995	T	C	0.66	0.0138	0.0024	5.1E-09	34.7
rs6012558	20	47531286	G	A	0.58	0.0145	0.0023	1.6E-10	41.9
rs9840902	3	78369573	G	A	0.78	-0.0149	0.0027	4.5E-08	31.0

SNP, single nucleotide polymorphisins; chr, chromosome; eaf, effect allele frequence; se, standard error.

Supplementary Table 4. Data sources for the confounding traits.

Confounding trait	Consortium or GWAS	Race	Number of cases	Number of controls
Lipids				
High-density lipoprotein cholesterol	UKBB and GLGC [1]	Transethnic	NA	188,578
Low-density lipoprotein cholesterol				
Total cholesterol				
Triglycerides				
Glycaemic traits				
Fasting glucose	MAGIC [2-4]	European	NA	46,186
Fasting insulin				
HOMA-B				
HOMA-IR				
HbA1c				123,665
Modified Stumvoll insulin sensitivity index				30,107
Blood Pressure				
Systolic blood pressure	UKBB and ICBP [5]	European	NA	757,601
Diastolic blood pressure				
Pulse pressure				
Body composition				
Body mass index	GIANT [6]	European	NA	694,649
Waist-to-hip ratio				
Habits and customs				
Smoking	GSCAN [7]	European	NA	1.2 million
Alcohol use				
Physical activity	UKBB [8]	European	NA	91,105

GWAS, Genome-wide Association Study; UKBB, UK Biobank; NA, not available; GLGC, Global Lipids Genetics Consortium; MAGIC, Meta-Analyses of Glucose and Insulin-related traits Consortium; ICBP, International Consortium of Blood Pressure; GIANT, Genetic Investigation of ANthropometric Traits; GSCAN, GWAS and Sequencing Consortium of Alcohol and Nicotine use.

Supplementary Table 5. Mendelian randomization association of genetically determined driving behavior with cerebrovascular disease traits and neurodegenerative disease traits.

Outcome	Driving				
	OR/beta (95%CI)	*p* value	I^2	P_Q	$P_{intercept}$
Cerebrovascular disease traits					
AS	1.03 (0.56, 1.89)	0.92	0	0.50	0.09
AIS	1.24 (0.61, 2.50)	0.55	7.4	0.36	0.05
CES	1.69 (0.48, 5.97)	0.42	0	0.67	0.15
LAS	2.45 (0.21, 28.54)	0.47	55.5	0.06	0.01
SVS	0.74 (0.08, 7.10)	0.79	54.9	0.06	0.64
ICH	0.25 (0.00, 48.54)	0.61	0	0.95	0.92
Nonlobar ICH	0.38 (0.00, 49.85)	0.86	38.5	0.16	0.72
Lobar ICH	0.20 (0.00, 224.15)	0.65	0	0.68	0.67
WMH	0.28 (-0.59, 1.14)	0.53	0	0.73	0.67
Neurodegenerative disease traits					
AD	1.26 (0.50, 3.19)	0.63	0	0.69	0.43
PD	0.64 (0.02, 16.68)	0.79	81.5	<0.001	0.90
MS	0.29 (0.02, 4.23)	0.36	0	0.97	0.93
CP	-0.80 (-1.4, -0.19)	0.01	89.1	<0.001	0.36

beta, the Mendelian randomization effect of continuous variable outcome, such as white matter hyperintensity and cognitive performance. OR, odds ratio; CI, confidence interval; P_Q, p value corresponding to Cochran Q test; $P_{intercept}$, p value corresponding to MR-Egger intercept test; AS, all-cause stroke; AIS, all-cause ischemic stroke; CES, cardioembolic stroke; LAS, large-artery atherosclerotic stroke; SVS, small vessel stroke; ICH, intracerebral hemorrhage; WMH, white matter hyperintensity; AD, Alzheimer's disease; PD, Parkinson’s disease; MS, multiple sclerosis; CP, cognitive performance.

Supplementary Table 6. Sensitivity analyses after removal of the outlier single nucleotide polymorphisms using IVW method.

Exposure	Outcome	OR (95%CI)	*p* value	I^2	P_Q	$P_{intercept}$
Television watching	AS	1.38 (1.21, 1.58)	1.1E-06	24.9%	0.01	0.16
Television watching	AIS	1.35 (1.17, 1.55)	3.1E-05	23.7%	0.01	0.23
Television watching	SVS	1.49 (1.09, 2.05)	1.2E-02	22.5%	0.01	0.49
Television watching	PD	0.69 (0.53, 0.88)	3.2E-03	7.9%	0.24	0.12
Television watching	CP	0.70 (0.66, 0.75)	5.3E-24	68.9%	<0.001	0.27
Computer use	CP	1.87 (1.67, 2.10)	8.3E-28	58.5%	<0.001	0.89

OR, odds ratio; CI, confidence interval; P_Q, p value corresponding to Cochran Q test; $P_{intercept}$, p value corresponding to MR-Egger intercept test; AS, all-cause stroke; AIS, all-cause ischemic stroke; SVS, small vessel stroke; PD, Parkinson’s disease; CP, cognitive performance.

Supplementary Table 7. Mendelian randomization association of genetically determined sedentary behaviors with blood pressure, serum lipid, glycemic, obesity and lifestyle traits.

Outcomes/Traits	Consortium	Television watching		Computer use		Driving	
		beta (95%CI)	*p* value	beta (95%CI)	*p* value	beta (95%CI)	*p* value
Systolic blood pressure	UKBB+ICBP	1.54 (0.62, 2.45)	1.01E-03	-3.76 (-6.09, -1.43)	1.58E-03	1.16 (-5.85, 8.17)	0.75
Diastolic blood pressure		1.04 (0.44, 1.63)	6.25E-04	-1.70 (-2.94, -0.45)	7.61E-03	0.60 (-3.10, 4.29)	0.75
Pulse pressure		0.51 (-0.02, 1.04)	0.06	-2.09 (-3.42, -0.75)	2.23E-03	0.65 (-3.17, 4.48)	0.74
High-density lipoprotein cholesterol	GLGC	-0.28 (-0.39, -0.17)	2.72E-07	0.07 (-0.07, 0.21)	0.33	-0.03 (-0.72, 0.66)	0.92
Low-density lipoprotein cholesterol		0.09 (-0.02, 0.21)	0.09	-0.25 (-0.5, 0.00)	0.05	0.31 (-0.47, 1.08)	0.43
Total cholesterol		0.04 (-0.08, 0.15)	0.52	-0.19 (-0.40, 0.02)	0.08	0.28 (-0.24, 0.80)	0.30
Triglyceride		0.21 (0.12, 0.30)	4.18E-06	-0.07 (-0.26, 0.11)	0.44	0.41 (0.00, 0.83)	0.05
Fasting glucose	MAGIC	0.11 (0.05, 0.17)	1.71E-04	-0.05 (-0.18, 0.08)	0.43	0.14 (-1.05, 1.33)	0.81
Fasting insulin		0.13 (0.07, 0.19)	6.30E-05	0.01 (-0.08, 0.10)	0.76	0.58 (-0.16, 1.32)	0.13
HOMA-B		0.06 (0.01, 0.11)	0.02	0.04 (-0.05, 0.12)	0.42	0.25 (-0.08, 0.57)	0.13
HOMA-IR		0.15 (0.09, 0.22)	1.61E-06	0.01 (-0.09, 0.11)	0.82	0.48 (-0.19, 1.14)	0.16
HbA1c		0.03 (0.00, 0.05)	0.06	0.01 (-0.04, 0.07)	0.59	-0.04 (-0.33, 0.24)	0.75
Insulin sensitivity index		-0.93 (-2.11, 0.25)	0.12	1.26 (-0.91, 3.44)	0.26	1.65 (-5.70, 8.99)	0.66
Body mass index	GIANT	0.39 (0.32, 0.46)	1.28E-26	0.06 (-0.07, 0.19)	0.35	0.10 (-0.47, 0.67)	0.73
Waist-to-hip ratio		0.27 (0.23, 0.32)	6.87E-30	-0.02 (-0.13, 0.09)	0.71	0.53 (-0.41, 1.48)	0.27
Smoking	GSCAN	-0.12 (-0.17, -0.06)	3.93E-05	0.00 (-0.09, 0.10)	0.94	-0.20 (-0.47, 0.07)	0.15
Alcohol use		0.05 (0.01, 0.09)	0.03	-0.04 (-0.13, 0.05)	0.35	-0.08 (-0.29, 0.13)	0.44
Physical activity	UKBB	-0.11 (-0.16, -0.05)	2.51E-04	-0.18 (-0.31, -0.05)	8.67E-03	0.56 (0.29, 0.84)	6.31E-05

HOMA-B, homeostatic model assessment of β-cell function; HOMA-IR, homeostatic model assessment of insulin resistance; UKBB, UK biobank; ICBP, International Consortium of Blood Pressure; GLGC, Global Lipids Genetics Consortium; MAGIC, Meta-Analyses of Glucose and Insulin-related traits Consortium; GIANT, Genetic Investigation of ANthropometric Traits; GSCAN, GWAS and Sequencing Consortium of Alcohol and Nicotine use; NA, not available; CI, confidence interval.

Supplementary Table 8. Multivariable Mendelian randomization of genetically determined sedentary behaviors and cognitive performance adjusted for confounding traits.

	Television watching		Computer use	
	beta (95%CI)	*p* value	beta (95%CI)	*p* value
Adjusted for blood pressure traits				
Systolic blood pressure	-0.48 (-0.56, -0.40)	3.37E-31	0.69 (0.54, 0.84)	5.28E-19
Diastolic blood pressure	-0.49 (-0.57, -0.41)	1.00E-32	0.66 (0.50, 0.81)	2.93E-17
Pulse pressure	-0.47 (-0.55, -0.39)	6.00E-32	0.70 (0.55, 0.85)	3.77E-20
Adjusted for serum lipid traits				
HDL-cholesterol	-0.41 (-0.53, -0.29)	2.72E-11	0.60 (0.41, 0.80)	1.14E-09
LDL-cholesterol	-0.49 (-0.61, -0.38)	1.38E-17	0.61 (0.40, 0.81)	9.49E-09
Total cholesterol	-0.49 (-0.60, -0.38)	3.39E-18	0.61 (0.41, 0.82)	3.85E-09
Triglyceride	-0.47 (-0.59, -0.34)	8.94E-14	0.63 (0.43, 0.82)	4.51E-10
Adjusted for glycemic traits				
Fasting glucose	-0.47 (-0.59, -0.35)	4.39E-14	0.63 (0.44, 0.83)	2.84E-10
Fasting insulin	-0.51 (-0.63, -0.38)	2.01E-15	0.62 (0.43, 0.82)	4.92E-10
HOMA-B	-0.50 (-0.62, -0.38)	6.60E-17	0.60 (0.40, 0.79)	1.07E-09
HOMA-IR	-0.49 (-0.62, -0.36)	6.98E-14	0.62 (0.42, 0.81)	3.70E-10
HbA1c	-0.48 (-0.59, -0.37)	1.14E-16	0.61 (0.43, 0.80)	1.33E-10
Insulin sensitivity index	-0.50 (-0.61, -0.38)	1.34E-17	0.62 (0.42, 0.82)	2.10E-09
Adjusted for obesity traits				
Body mass index	-0.43 (-0.53, -0.33)	3.38E-17	0.63 (0.48, 0.77)	1.84E-17
Waist-to-hip ratio	-0.39 (-0.49, -0.29)	4.58E-14	0.63 (0.48, 0.77)	8.99E-18
Adjusted for lifestyle traits				
Smoking	-0.48 (-0.56, -0.40)	3.17E-30	0.63 (0.49, 0.77)	2.37E-18
Alcohol use	-0.46 (-0.54, -0.38)	2.49E-31	0.61 (0.47, 0.75)	1.58E-17
Physical activity	-0.51 (-0.59, -0.43)	2.50E-37	0.61 (0.46, 0.77)	1.78E-15

HOMA-B, homeostatic model assessment of β-cell function; HOMA-IR, homeostatic model assessment of insulin resistance; HDL-cholesterol, high-density lipoprotein cholesterol; LDL-cholesterol, low-density lipoprotein cholesterol; OR, odds ratio; CI, confidence interval.

Supplementary Table 9. Summary data for the genetic association between television watching and all-cause stroke.

SNP	effect_allele	eaf.exposure	eaf.outcome	beta.exposure	se.exposure	beta.outcome	se.outcome	proxy
rs10041724	T	0.81	0.81	0.018	0.003	0.007	0.012	
rs10054327	G	0.58	0.59	0.017	0.002	-0.033	0.009	
rs10145592	C	0.41	0.40	-0.015	0.002	-0.001	0.009	
rs10189857	A	0.57	0.56	-0.021	0.002	-0.010	0.009	
rs1022785	G	0.14	0.15	0.018	0.003	0.032	0.013	
rs10234444	G	0.82	0.83	0.016	0.003	0.027	0.013	
rs10246289	A	0.11	0.12	0.019	0.003	0.018	0.014	
rs1031423	T	0.22	0.20	-0.019	0.003	0.005	0.011	
rs10427502	G	0.62	0.64	0.014	0.002	-0.008	0.010	
rs10737620	T	0.27	0.28	0.014	0.002	-0.002	0.010	
rs10771746	C	0.72	0.72	-0.014	0.002	0.009	0.010	
rs10772643	C	0.11	0.12	0.025	0.004	-0.005	0.015	
rs10876864	G	0.43	0.41	-0.013	0.002	-0.013	0.009	
rs10890123	C	0.77	0.79	0.014	0.003	-0.004	0.011	
rs10932837	C	0.49	0.49	-0.013	0.002	0.000	0.009	
rs10940659	A	0.53	0.53	-0.013	0.002	0.013	0.009	
rs10994943	T	0.58	0.61	0.013	0.002	0.018	0.009	
rs11020045	A	0.67	0.67	-0.013	0.002	-0.023	0.010	
rs11130793	C	0.60	0.63	0.013	0.002	0.021	0.010	
rs111901094	G	0.82	0.88	-0.017	0.003	0.020	0.014	rs57962361
rs11201422	T	0.67	0.68	0.013	0.002	0.004	0.010	
rs11218575	C	0.57	0.58	0.015	0.002	0.006	0.010	
rs11245482	T	0.61	0.62	-0.013	0.002	-0.018	0.010	

rs114328297	T	0.78	0.78	0.014	0.003	-0.003	0.012	
rs114600294	G	0.79	0.82	-0.016	0.003	0.030	0.016	
rs1156541	C	0.22	0.23	0.015	0.003	0.000	0.011	
rs11654952	T	0.86	0.86	-0.017	0.003	-0.022	0.014	
rs11657730	C	0.64	0.63	0.013	0.002	-0.002	0.011	
rs11689199	A	0.60	0.63	0.019	0.002	-0.002	0.010	
rs11714337	G	0.57	0.57	0.014	0.002	0.014	0.010	
rs11763734	A	0.51	0.52	-0.013	0.002	0.006	0.009	
rs11810109	A	0.70	0.70	0.016	0.002	0.018	0.010	
rs12105701	C	0.40	0.39	-0.013	0.002	-0.004	0.010	
rs12272012	G	0.96	0.95	-0.030	0.005	0.004	0.022	
rs12289262	C	0.73	0.75	-0.014	0.002	0.005	0.011	
rs1243182	C	0.69	0.68	-0.019	0.002	0.020	0.010	
rs12476388	C	0.71	0.71	0.013	0.002	0.005	0.011	
rs12491503	G	0.67	0.68	-0.014	0.002	-0.028	0.010	
rs12541615	T	0.82	0.81	-0.018	0.003	-0.007	0.012	
rs12554512	T	0.58	0.60	0.021	0.002	0.020	0.010	
rs12725114	G	0.80	0.79	0.015	0.003	0.008	0.011	
rs1278847	C	0.69	0.70	0.016	0.002	0.020	0.011	
rs13029509	G	0.52	0.52	-0.018	0.002	0.007	0.009	
rs13107325	C	0.93	0.93	-0.029	0.004	0.001	0.020	
rs138256022	C	0.96	0.95	-0.031	0.006	0.011	0.026	
rs1421334	A	0.45	0.46	0.017	0.002	-0.015	0.009	
rs1451533	G	0.72	0.72	-0.016	0.002	0.001	0.010	
rs17207890	G	0.66	0.65	0.016	0.002	0.006	0.010	

rs17379561	A	0.86	0.85	-0.026	0.003	-0.004	0.013	
rs17512836	T	0.97	0.97	0.042	0.007	0.066	0.031	
rs17727474	C	0.83	0.81	0.018	0.003	0.019	0.012	
rs17789218	T	0.76	0.77	0.019	0.003	-0.015	0.011	
rs2034768	A	0.49	0.47	0.015	0.002	0.000	0.009	
rs2045147	A	0.45	0.46	0.013	0.002	-0.008	0.009	
rs2073869	C	0.83	0.82	0.019	0.003	-0.003	0.012	
rs2092829	G	0.71	0.69	0.014	0.002	-0.017	0.011	
rs2164744	T	0.64	0.62	-0.013	0.002	0.008	0.009	
rs2173650	G	0.85	0.84	0.018	0.003	-0.016	0.013	
rs2184364	A	0.78	0.80	0.016	0.003	-0.013	0.013	
rs2447098	C	0.48	0.48	-0.015	0.002	0.001	0.009	
rs2460	G	0.74	0.75	-0.015	0.003	0.008	0.011	
rs2584597	T	0.66	0.71	0.015	0.002	0.018	0.011	
rs2616830	G	0.46	0.45	0.017	0.002	-0.006	0.009	
rs262890	A	0.70	0.70	-0.019	0.002	-0.014	0.010	
rs2717559	A	0.56	0.57	0.012	0.002	-0.001	0.011	
rs2787374	T	0.41	0.42	0.013	0.002	0.019	0.009	
rs303753	G	0.65	0.64	-0.015	0.002	-0.036	0.010	
rs34864022	A	0.93	0.92	-0.026	0.004	-0.005	0.018	
rs35574015	T	0.71	0.70	-0.013	0.002	-0.003	0.010	
rs374722	G	0.15	0.16	0.025	0.003	0.023	0.013	
rs3754970	T	0.50	0.51	-0.015	0.002	0.005	0.009	
rs3796386	G	0.57	0.58	-0.026	0.002	-0.024	0.010	
rs405797	T	0.25	0.30	-0.015	0.003	-0.011	0.012	

rs42210	G	0.29	0.29	-0.014	0.002	0.003	0.011	
rs4334769	G	0.47	0.48	0.012	0.002	0.001	0.009	
rs4382592	T	0.30	0.31	0.014	0.002	0.029	0.010	
rs4523073	A	0.59	0.61	-0.014	0.002	0.001	0.009	
rs4577309	A	0.47	0.47	0.016	0.002	0.009	0.009	
rs4675246	G	0.80	0.80	-0.015	0.003	-0.011	0.012	
rs4775373	T	0.36	0.36	0.013	0.002	0.006	0.010	
rs4845364	A	0.50	0.49	-0.015	0.002	0.013	0.009	
rs4937842	G	0.63	0.61	-0.013	0.002	-0.001	0.010	
rs4973576	C	0.30	0.30	-0.015	0.002	-0.006	0.010	
rs55700114	G	0.71	0.68	-0.014	0.002	-0.007	0.010	
rs55909997	G	0.65	0.66	-0.014	0.002	-0.003	0.010	
rs56103247	C	0.94	0.95	0.030	0.005	0.038	0.024	
rs56858768	G	0.70	0.70	-0.015	0.002	-0.009	0.010	
rs57585211	T	0.83	0.83	-0.017	0.003	-0.022	0.012	
rs6131281	C	0.60	0.60	0.016	0.002	-0.008	0.010	
rs6141814	C	0.61	0.63	-0.014	0.002	0.001	0.009	
rs62379379	G	0.93	0.92	-0.026	0.004	-0.023	0.018	
rs62641636	A	0.69	0.70	0.014	0.002	0.008	0.010	
rs6472942	T	0.57	0.57	-0.013	0.002	-0.012	0.009	
rs6673341	T	0.47	0.47	-0.015	0.002	-0.003	0.009	
rs66852340	C	0.78	0.78	-0.018	0.003	-0.018	0.012	
rs6721975	T	0.23	0.28	-0.017	0.003	-0.037	0.012	
rs6797840	A	0.46	0.46	-0.016	0.002	-0.002	0.010	
rs6825241	C	0.54	0.55	-0.017	0.002	-0.022	0.010	

rs6850494	A	0.62	0.60	-0.014	0.002	-0.002	0.010	
rs6905544	A	0.40	0.40	-0.019	0.002	-0.007	0.009	
rs6973656	A	0.60	0.61	-0.014	0.002	-0.012	0.009	
rs6996198	C	0.84	0.84	-0.016	0.003	-0.024	0.013	
rs7089973	C	0.62	0.63	-0.013	0.002	0.011	0.010	
rs7157001	A	0.74	0.73	-0.014	0.003	-0.007	0.012	
rs71658797	T	0.88	0.89	-0.020	0.003	-0.025	0.015	
rs7184800	G	0.70	0.69	0.017	0.002	0.007	0.010	
rs7189927	T	0.36	0.35	0.015	0.002	-0.004	0.010	
rs7248205	C	0.40	0.40	0.014	0.002	-0.042	0.010	
rs72671494	T	0.86	0.86	-0.017	0.003	-0.002	0.014	
rs72781699	G	0.80	0.80	-0.019	0.003	-0.001	0.011	
rs72828890	C	0.87	0.87	0.019	0.003	0.002	0.015	
rs72834698	G	0.86	0.86	0.023	0.003	0.009	0.014	
rs749671	G	0.63	0.61	0.016	0.002	-0.015	0.010	
rs7564130	T	0.64	0.63	-0.015	0.002	-0.012	0.010	
rs7693082	G	0.30	0.30	0.015	0.002	0.005	0.010	
rs7693703	G	0.91	0.90	0.023	0.004	0.015	0.016	
rs7700107	A	0.86	0.87	-0.021	0.003	-0.032	0.014	
rs7716447	A	0.64	0.71	-0.013	0.002	0.003	0.012	
rs77215114	A	0.93	0.92	0.024	0.004	0.045	0.019	
rs7834121	G	0.50	0.51	-0.014	0.002	0.016	0.009	rs7843666
rs7991062	C	0.66	0.65	-0.018	0.002	-0.010	0.010	
rs801733	A	0.64	0.65	0.017	0.002	-0.001	0.010	
rs8043253	C	0.57	0.57	-0.012	0.002	-0.010	0.009	

rs8756	C	0.48	0.49	-0.014	0.002	-0.011	0.010	
rs9471333	C	0.45	0.44	0.013	0.002	-0.001	0.009	
rs9563168	G	0.79	0.79	0.018	0.003	0.020	0.011	
rs9569734	A	0.84	0.86	0.019	0.003	-0.003	0.014	
rs9718104	T	0.94	0.94	-0.041	0.005	-0.031	0.023	
rs973734	C	0.15	0.15	0.017	0.003	0.005	0.013	
rs9834970	T	0.50	0.50	0.013	0.002	0.000	0.010	
rs984409	G	0.36	0.36	-0.015	0.002	-0.007	0.009	
rs9867121	C	0.82	0.82	0.020	0.003	-0.004	0.012	
rs9902312	T	0.68	0.67	0.015	0.002	0.008	0.010	
rs9964724	C	0.32	0.32	0.018	0.002	-0.005	0.010	

Supplementary Table 10. Summary data for the genetic association between television watching and all-cause ischemic stroke.

SNP	effect_allele	eaf.exposure	eaf.outcome	beta.exposure	se.exposure	beta.outcome	se.outcome	proxy
rs10041724	T	0.81	0.81	0.018	0.003	-0.001	0.013	
rs10054327	G	0.58	0.59	0.017	0.002	-0.035	0.010	
rs10145592	C	0.41	0.40	-0.015	0.002	0.005	0.010	
rs10189857	A	0.57	0.56	-0.021	0.002	-0.008	0.010	
rs1022785	G	0.14	0.15	0.018	0.003	0.038	0.014	
rs10234444	G	0.82	0.83	0.016	0.003	0.025	0.014	
rs10246289	A	0.11	0.12	0.019	0.003	0.020	0.016	
rs1031423	T	0.22	0.20	-0.019	0.003	0.006	0.013	
rs10427502	G	0.62	0.64	0.014	0.002	-0.003	0.010	
rs10737620	T	0.27	0.28	0.014	0.002	-0.009	0.011	
rs10771746	C	0.72	0.71	-0.014	0.002	0.015	0.011	
rs10772643	C	0.11	0.12	0.025	0.004	-0.007	0.016	
rs10876864	G	0.43	0.41	-0.013	0.002	-0.008	0.010	
rs10890123	C	0.77	0.79	0.014	0.003	-0.008	0.012	
rs10932837	C	0.49	0.49	-0.013	0.002	-0.003	0.010	
rs10940659	A	0.53	0.53	-0.013	0.002	0.008	0.010	
rs10994943	T	0.58	0.61	0.013	0.002	0.022	0.010	
rs11020045	A	0.67	0.67	-0.013	0.002	-0.025	0.011	
rs11130793	C	0.60	0.63	0.013	0.002	0.019	0.011	
rs111901094	G	0.82	0.88	-0.017	0.003	0.031	0.014	rs57962361
rs11201422	T	0.67	0.68	0.013	0.002	0.009	0.011	
rs11218575	C	0.57	0.59	0.015	0.002	0.009	0.010	
rs11245482	T	0.61	0.62	-0.013	0.002	-0.013	0.010	

rs114328297	T	0.78	0.78	0.014	0.003	0.001	0.013	
rs114600294	G	0.79	0.82	-0.016	0.003	0.028	0.016	
rs1156541	C	0.22	0.23	0.015	0.003	-0.005	0.012	
rs11654952	T	0.86	0.86	-0.017	0.003	-0.021	0.014	
rs11657730	C	0.64	0.63	0.013	0.002	0.003	0.012	
rs11689199	A	0.60	0.63	0.019	0.002	-0.003	0.010	
rs11714337	G	0.57	0.57	0.014	0.002	0.014	0.010	
rs11763734	A	0.51	0.52	-0.013	0.002	0.004	0.010	
rs11810109	A	0.70	0.70	0.016	0.002	0.014	0.011	
rs12105701	C	0.40	0.39	-0.013	0.002	-0.003	0.011	
rs12272012	G	0.96	0.95	-0.030	0.005	0.011	0.025	
rs12289262	C	0.73	0.75	-0.014	0.002	0.007	0.012	
rs1243182	C	0.69	0.68	-0.019	0.002	0.030	0.012	
rs12476388	C	0.71	0.71	0.013	0.002	0.006	0.011	
rs12491503	G	0.67	0.68	-0.014	0.002	-0.019	0.011	
rs12541615	T	0.82	0.81	-0.018	0.003	-0.008	0.013	
rs12554512	T	0.58	0.60	0.021	0.002	0.018	0.010	
rs12725114	G	0.80	0.79	0.015	0.003	0.016	0.013	
rs1278847	C	0.69	0.71	0.016	0.002	0.017	0.012	
rs13029509	G	0.52	0.52	-0.018	0.002	-0.001	0.010	
rs13107325	C	0.93	0.93	-0.029	0.004	0.007	0.022	
rs138256022	C	0.96	0.95	-0.031	0.006	0.009	0.029	
rs1421334	A	0.45	0.46	0.017	0.002	-0.011	0.010	
rs1451533	G	0.72	0.72	-0.016	0.002	-0.001	0.012	
rs17207890	G	0.66	0.65	0.016	0.002	0.015	0.011	

rs17379561	A	0.86	0.85	-0.026	0.003	-0.001	0.014	
rs17512836	T	0.97	0.97	0.042	0.007	0.044	0.033	
rs17727474	C	0.83	0.81	0.018	0.003	0.019	0.013	
rs17789218	T	0.76	0.77	0.019	0.003	-0.015	0.013	
rs2034768	A	0.49	0.47	0.015	0.002	-0.004	0.010	
rs2045147	A	0.45	0.46	0.013	0.002	-0.006	0.010	
rs2073869	C	0.83	0.82	0.019	0.003	-0.009	0.013	
rs2092829	G	0.71	0.69	0.014	0.002	-0.019	0.011	
rs2164744	T	0.64	0.63	-0.013	0.002	0.004	0.010	
rs2173650	G	0.85	0.84	0.018	0.003	-0.024	0.014	
rs2184364	A	0.78	0.80	0.016	0.003	-0.016	0.014	
rs2447098	C	0.48	0.48	-0.015	0.002	0.005	0.010	
rs2460	G	0.74	0.75	-0.015	0.003	0.015	0.012	
rs2584597	T	0.66	0.72	0.015	0.002	0.020	0.013	
rs2616830	G	0.46	0.45	0.017	0.002	-0.005	0.010	
rs262890	A	0.70	0.70	-0.019	0.002	-0.016	0.011	
rs2717559	A	0.56	0.57	0.012	0.002	-0.010	0.011	
rs2787374	T	0.41	0.41	0.013	0.002	0.018	0.010	
rs303753	G	0.65	0.64	-0.015	0.002	-0.034	0.011	
rs34864022	A	0.93	0.92	-0.026	0.004	-0.014	0.019	
rs35574015	T	0.71	0.70	-0.013	0.002	-0.001	0.011	
rs374722	G	0.15	0.16	0.025	0.003	0.025	0.014	
rs3754970	T	0.50	0.51	-0.015	0.002	0.001	0.010	
rs3796386	G	0.57	0.58	-0.026	0.002	-0.022	0.011	
rs405797	T	0.25	0.30	-0.015	0.003	-0.019	0.013	

rs42210	G	0.29	0.29	-0.014	0.002	0.006	0.012	
rs4334769	G	0.47	0.48	0.012	0.002	0.002	0.010	
rs4382592	T	0.30	0.31	0.014	0.002	0.028	0.011	
rs4523073	A	0.59	0.61	-0.014	0.002	-0.005	0.010	
rs4577309	A	0.47	0.47	0.016	0.002	0.014	0.010	
rs4675246	G	0.80	0.80	-0.015	0.003	-0.015	0.013	
rs4775373	T	0.36	0.36	0.013	0.002	0.009	0.010	
rs4845364	A	0.50	0.49	-0.015	0.002	0.016	0.010	
rs4937842	G	0.63	0.61	-0.013	0.002	0.006	0.011	
rs4973576	C	0.30	0.30	-0.015	0.002	-0.004	0.011	
rs55700114	G	0.71	0.68	-0.014	0.002	0.002	0.011	
rs55909997	G	0.65	0.66	-0.014	0.002	-0.003	0.011	
rs56103247	C	0.94	0.95	0.030	0.005	0.031	0.026	
rs56858768	G	0.70	0.70	-0.015	0.002	-0.006	0.011	
rs57585211	T	0.83	0.83	-0.017	0.003	-0.027	0.014	
rs6131281	C	0.60	0.60	0.016	0.002	-0.014	0.010	
rs6141814	C	0.61	0.63	-0.014	0.002	0.004	0.010	
rs62379379	G	0.93	0.92	-0.026	0.004	-0.018	0.019	
rs62641636	A	0.69	0.70	0.014	0.002	0.011	0.011	
rs6472942	T	0.57	0.57	-0.013	0.002	-0.018	0.010	
rs6673341	T	0.47	0.47	-0.015	0.002	-0.006	0.010	
rs66852340	C	0.78	0.78	-0.018	0.003	-0.015	0.013	
rs6721975	T	0.23	0.28	-0.017	0.003	-0.030	0.013	
rs6797840	A	0.46	0.46	-0.016	0.002	0.000	0.010	
rs6825241	C	0.54	0.54	-0.017	0.002	-0.023	0.010	

rs6850494	A	0.62	0.60	-0.014	0.002	-0.003	0.010	
rs6905544	A	0.40	0.40	-0.019	0.002	-0.008	0.010	
rs6973656	A	0.60	0.61	-0.014	0.002	-0.013	0.010	
rs6996198	C	0.84	0.84	-0.016	0.003	-0.027	0.014	
rs7089973	C	0.62	0.63	-0.013	0.002	0.014	0.010	
rs7157001	A	0.74	0.73	-0.014	0.003	-0.009	0.013	
rs71658797	T	0.88	0.89	-0.020	0.003	-0.025	0.016	
rs7184800	G	0.70	0.69	0.017	0.002	0.003	0.011	
rs7189927	T	0.36	0.36	0.015	0.002	0.002	0.011	
rs7248205	C	0.40	0.40	0.014	0.002	-0.044	0.011	
rs72671494	T	0.86	0.86	-0.017	0.003	-0.008	0.015	
rs72781699	G	0.80	0.80	-0.019	0.003	-0.007	0.012	
rs72828890	C	0.87	0.87	0.019	0.003	-0.008	0.016	
rs72834698	G	0.86	0.86	0.023	0.003	0.001	0.015	
rs749671	G	0.63	0.61	0.016	0.002	-0.015	0.011	
rs7564130	T	0.64	0.63	-0.015	0.002	-0.004	0.012	
rs7693082	G	0.30	0.30	0.015	0.002	0.004	0.011	
rs7693703	G	0.91	0.90	0.023	0.004	0.022	0.018	
rs7700107	A	0.86	0.87	-0.021	0.003	-0.028	0.015	
rs7716447	A	0.64	0.71	-0.013	0.002	0.007	0.013	
rs77215114	A	0.93	0.92	0.024	0.004	0.036	0.021	
rs7834121	G	0.50	0.51	-0.014	0.002	0.020	0.009	rs7843666
rs7991062	C	0.66	0.65	-0.018	0.002	-0.010	0.011	
rs801733	A	0.64	0.65	0.017	0.002	0.007	0.011	
rs8043253	C	0.57	0.57	-0.012	0.002	-0.016	0.010	

rs8756	C	0.48	0.49	-0.014	0.002	-0.013	0.010	
rs9471333	C	0.45	0.44	0.013	0.002	-0.007	0.010	
rs9563168	G	0.79	0.79	0.018	0.003	0.017	0.012	
rs9569734	A	0.84	0.86	0.019	0.003	-0.004	0.015	
rs9718104	T	0.94	0.94	-0.041	0.005	-0.047	0.025	
rs973734	C	0.15	0.15	0.017	0.003	0.010	0.014	
rs9834970	T	0.50	0.51	0.013	0.002	-0.004	0.010	
rs984409	G	0.36	0.36	-0.015	0.002	-0.011	0.011	
rs9867121	C	0.82	0.81	0.020	0.003	-0.001	0.014	
rs9902312	T	0.68	0.67	0.015	0.002	0.008	0.010	
rs9964724	C	0.32	0.32	0.018	0.002	-0.006	0.011	

Supplementary Table 11. Summary data for the genetic association between television watching and small vessel stroke.

SNP	effect_allele	eaf.exposure	eaf.outcome	beta.exposure	se.exposure	beta.outcome	se.outcome	proxy
rs10041724	T	0.81	0.81	0.018	0.003	-0.024	0.030	
rs10054327	G	0.58	0.58	0.017	0.002	-0.049	0.023	
rs10145592	C	0.41	0.40	-0.015	0.002	0.027	0.023	
rs10189857	A	0.57	0.56	-0.021	0.002	-0.049	0.023	
rs1022785	G	0.14	0.15	0.018	0.003	0.055	0.033	
rs10234444	G	0.82	0.83	0.016	0.003	0.043	0.031	
rs10246289	A	0.11	0.12	0.019	0.003	0.024	0.036	
rs1031423	T	0.22	0.21	-0.019	0.003	-0.001	0.029	
rs10427502	G	0.62	0.63	0.014	0.002	-0.005	0.024	
rs10737620	T	0.27	0.28	0.014	0.002	0.002	0.026	
rs10771746	C	0.72	0.72	-0.014	0.002	0.050	0.026	
rs10772643	C	0.11	0.11	0.025	0.004	-0.041	0.038	
rs10876864	G	0.43	0.42	-0.013	0.002	-0.013	0.023	
rs10890123	C	0.77	0.78	0.014	0.003	-0.011	0.028	
rs10932837	C	0.49	0.49	-0.013	0.002	0.032	0.023	
rs10940659	A	0.53	0.53	-0.013	0.002	0.028	0.023	
rs10994943	T	0.58	0.61	0.013	0.002	0.042	0.024	
rs11020045	A	0.67	0.67	-0.013	0.002	0.009	0.024	
rs11130793	C	0.60	0.62	0.013	0.002	0.016	0.024	
rs111901094	G	0.82	0.88	-0.017	0.003	-0.002	0.014	rs57962361
rs11201422	T	0.67	0.68	0.013	0.002	-0.001	0.025	
rs11218575	C	0.57	0.58	0.015	0.002	0.001	0.024	
rs11245482	T	0.61	0.62	-0.013	0.002	-0.042	0.023	

rs114328297	T	0.78	0.79	0.014	0.003	0.020	0.030	
rs114600294	G	0.79	0.82	-0.016	0.003	0.020	0.036	
rs1156541	C	0.22	0.23	0.015	0.003	-0.003	0.028	
rs11654952	T	0.86	0.86	-0.017	0.003	-0.054	0.035	
rs11657730	C	0.64	0.64	0.013	0.002	0.061	0.027	
rs11689199	A	0.60	0.64	0.019	0.002	0.015	0.025	
rs11714337	G	0.57	0.57	0.014	0.002	0.008	0.024	
rs11763734	A	0.51	0.52	-0.013	0.002	0.007	0.023	
rs11810109	A	0.70	0.70	0.016	0.002	0.019	0.025	
rs12105701	C	0.40	0.39	-0.013	0.002	-0.008	0.024	
rs12272012	G	0.96	0.95	-0.030	0.005	-0.056	0.058	
rs12289262	C	0.73	0.75	-0.014	0.002	0.011	0.027	
rs1243182	C	0.69	0.68	-0.019	0.002	0.050	0.025	
rs12476388	C	0.71	0.71	0.013	0.002	0.044	0.026	
rs12491503	G	0.67	0.68	-0.014	0.002	-0.020	0.025	
rs12541615	T	0.82	0.81	-0.018	0.003	0.006	0.029	
rs12554512	T	0.58	0.60	0.021	0.002	0.031	0.024	
rs12725114	G	0.80	0.79	0.015	0.003	0.003	0.028	
rs1278847	C	0.69	0.72	0.016	0.002	0.033	0.027	
rs13029509	G	0.52	0.52	-0.018	0.002	0.015	0.023	
rs13107325	C	0.93	0.92	-0.029	0.004	-0.023	0.048	
rs138256022	C	0.96	0.95	-0.031	0.006	0.021	0.066	
rs1421334	A	0.45	0.46	0.017	0.002	0.043	0.024	
rs1451533	G	0.72	0.73	-0.016	0.002	0.004	0.027	
rs17207890	G	0.66	0.65	0.016	0.002	-0.045	0.024	

rs17379561	A	0.86	0.85	-0.026	0.003	-0.006	0.032	
rs17512836	T	0.97	0.97	0.042	0.007	0.022	0.077	
rs17727474	C	0.83	0.81	0.018	0.003	0.080	0.031	
rs17789218	T	0.76	0.77	0.019	0.003	-0.014	0.029	
rs2034768	A	0.49	0.48	0.015	0.002	0.011	0.023	
rs2045147	A	0.45	0.46	0.013	0.002	-0.006	0.023	
rs2073869	C	0.83	0.83	0.019	0.003	-0.015	0.031	
rs2092829	G	0.71	0.70	0.014	0.002	-0.035	0.025	
rs2164744	T	0.64	0.62	-0.013	0.002	-0.005	0.023	
rs2173650	G	0.85	0.84	0.018	0.003	-0.031	0.032	
rs2184364	A	0.78	0.80	0.016	0.003	-0.041	0.030	
rs2447098	C	0.48	0.48	-0.015	0.002	-0.022	0.023	
rs2460	G	0.74	0.75	-0.015	0.003	0.034	0.027	
rs2584597	T	0.66	0.72	0.015	0.002	0.018	0.026	
rs2616830	G	0.46	0.45	0.017	0.002	-0.036	0.023	
rs262890	A	0.70	0.70	-0.019	0.002	0.011	0.025	
rs2717559	A	0.56	0.57	0.012	0.002	-0.002	0.024	
rs2787374	T	0.41	0.42	0.013	0.002	-0.012	0.023	
rs303753	G	0.65	0.65	-0.015	0.002	-0.022	0.024	
rs34864022	A	0.93	0.92	-0.026	0.004	-0.050	0.045	
rs35574015	T	0.71	0.70	-0.013	0.002	0.026	0.025	
rs374722	G	0.15	0.16	0.025	0.003	0.044	0.032	
rs3754970	T	0.50	0.50	-0.015	0.002	-0.014	0.023	
rs3796386	G	0.57	0.58	-0.026	0.002	-0.025	0.025	
rs405797	T	0.25	0.32	-0.015	0.003	-0.021	0.028	

rs42210	G	0.29	0.29	-0.014	0.002	-0.008	0.027	
rs4334769	G	0.47	0.48	0.012	0.002	0.013	0.023	
rs4382592	T	0.30	0.31	0.014	0.002	0.027	0.025	
rs4523073	A	0.59	0.61	-0.014	0.002	-0.031	0.024	
rs4577309	A	0.47	0.47	0.016	0.002	0.038	0.023	
rs4675246	G	0.80	0.80	-0.015	0.003	-0.053	0.029	
rs4775373	T	0.36	0.36	0.013	0.002	0.048	0.024	
rs4845364	A	0.50	0.49	-0.015	0.002	0.043	0.023	
rs4937842	G	0.63	0.61	-0.013	0.002	0.040	0.026	
rs4973576	C	0.30	0.30	-0.015	0.002	0.046	0.025	
rs55700114	G	0.71	0.69	-0.014	0.002	-0.012	0.024	
rs55909997	G	0.65	0.66	-0.014	0.002	-0.032	0.024	
rs56103247	C	0.94	0.94	0.030	0.005	-0.002	0.062	
rs56858768	G	0.70	0.70	-0.015	0.002	-0.007	0.026	
rs57585211	T	0.83	0.83	-0.017	0.003	-0.003	0.031	
rs6131281	C	0.60	0.60	0.016	0.002	-0.034	0.023	
rs6141814	C	0.61	0.63	-0.014	0.002	0.008	0.024	
rs62379379	G	0.93	0.92	-0.026	0.004	-0.053	0.045	
rs62641636	A	0.69	0.70	0.014	0.002	0.001	0.025	
rs6472942	T	0.57	0.56	-0.013	0.002	-0.030	0.023	
rs6673341	T	0.47	0.47	-0.015	0.002	-0.012	0.024	
rs66852340	C	0.78	0.78	-0.018	0.003	-0.026	0.028	
rs6721975	T	0.23	0.28	-0.017	0.003	-0.030	0.029	
rs6797840	A	0.46	0.47	-0.016	0.002	0.017	0.024	
rs6825241	C	0.54	0.54	-0.017	0.002	-0.049	0.023	

rs6850494	A	0.62	0.61	-0.014	0.002	0.030	0.024	
rs6905544	A	0.40	0.40	-0.019	0.002	-0.035	0.024	
rs6973656	A	0.60	0.61	-0.014	0.002	-0.022	0.023	
rs6996198	C	0.84	0.85	-0.016	0.003	-0.033	0.032	
rs7089973	C	0.62	0.63	-0.013	0.002	0.020	0.024	
rs7157001	A	0.74	0.74	-0.014	0.003	-0.007	0.030	
rs71658797	T	0.88	0.89	-0.020	0.003	-0.038	0.036	
rs7184800	G	0.70	0.69	0.017	0.002	0.004	0.025	
rs7189927	T	0.36	0.36	0.015	0.002	-0.010	0.024	
rs7248205	C	0.40	0.41	0.014	0.002	0.004	0.024	
rs72671494	T	0.86	0.86	-0.017	0.003	0.004	0.036	
rs72781699	G	0.80	0.80	-0.019	0.003	-0.015	0.028	
rs72828890	C	0.87	0.87	0.019	0.003	-0.035	0.037	
rs72834698	G	0.86	0.86	0.023	0.003	-0.058	0.034	
rs749671	G	0.63	0.62	0.016	0.002	-0.036	0.024	
rs7564130	T	0.64	0.64	-0.015	0.002	-0.006	0.024	
rs7693082	G	0.30	0.30	0.015	0.002	0.028	0.025	
rs7693703	G	0.91	0.91	0.023	0.004	0.060	0.042	
rs7700107	A	0.86	0.87	-0.021	0.003	-0.119	0.034	
rs7716447	A	0.64	0.71	-0.013	0.002	-0.032	0.028	
rs77215114	A	0.93	0.92	0.024	0.004	0.059	0.050	
rs7834121	G	0.50	0.49	-0.014	0.002	-0.039	0.009	rs7843666
rs7991062	C	0.66	0.65	-0.018	0.002	-0.049	0.025	
rs801733	A	0.64	0.65	0.017	0.002	0.044	0.024	
rs8043253	C	0.57	0.57	-0.012	0.002	-0.058	0.023	

rs8756	C	0.48	0.48	-0.014	0.002	-0.029	0.025	
rs9471333	C	0.45	0.44	0.013	0.002	-0.012	0.023	
rs9563168	G	0.79	0.79	0.018	0.003	0.011	0.028	
rs9569734	A	0.84	0.85	0.019	0.003	-0.063	0.033	
rs9718104	T	0.94	0.94	-0.041	0.005	0.013	0.061	
rs973734	C	0.15	0.15	0.017	0.003	0.002	0.033	
rs9834970	T	0.50	0.51	0.013	0.002	-0.012	0.023	
rs984409	G	0.36	0.36	-0.015	0.002	-0.025	0.024	
rs9867121	C	0.82	0.82	0.020	0.003	-0.002	0.030	
rs9902312	T	0.68	0.67	0.015	0.002	-0.027	0.024	
rs9964724	C	0.32	0.32	0.018	0.002	0.015	0.025	

Supplementary Table 12. Summary data for the genetic association between television watching and Parkinson's disease.

SNP	effect_allele	eaf.exposure	eaf.outcome	beta.exposure	se.exposure	beta.outcome	se.outcome	proxy
rs10041724	T	0.81	0.80	0.018	0.003	-0.087	0.028	
rs10054327	G	0.58	0.59	0.017	0.002	0.006	0.017	
rs10145592	C	0.41	0.40	-0.015	0.002	-0.002	0.018	
rs10189857	A	0.57	0.58	-0.021	0.002	0.036	0.017	
rs1022785	G	0.14	0.15	0.018	0.003	0.000	0.031	
rs10234444	G	0.82	0.83	0.016	0.003	-0.009	0.030	
rs10246289	A	0.11	0.11	0.019	0.003	0.019	0.036	
rs1031423	T	0.22	0.21	-0.019	0.003	0.044	0.025	
rs10427502	G	0.62	0.63	0.014	0.002	0.012	0.020	
rs10737620	T	0.27	0.27	0.014	0.002	0.008	0.021	
rs10771746	C	0.72	0.72	-0.014	0.002	0.044	0.020	
rs10772643	C	0.11	0.11	0.025	0.004	-0.002	0.037	
rs10876864	G	0.43	0.42	-0.013	0.002	0.011	0.018	
rs10890123	C	0.77	0.79	0.014	0.003	-0.016	0.024	
rs10932837	C	0.49	0.49	-0.013	0.002	0.006	0.022	
rs10940659	A	0.53	0.53	-0.013	0.002	-0.004	0.021	
rs10994943	T	0.58	0.60	0.013	0.002	-0.020	0.018	
rs11020045	A	0.67	0.68	-0.013	0.002	-0.003	0.021	rs1792360
rs11130793	C	0.60	0.63	0.013	0.002	0.004	0.021	
rs111901094	G	0.82	0.11	-0.017	0.003	-0.045	0.029	
rs11201422	T	0.67	0.68	0.013	0.002	-0.016	0.024	
rs11218575	C	0.57	0.58	0.015	0.002	0.013	0.019	
rs11245482	T	0.61	0.62	-0.013	0.002	-0.024	0.019	

rs114328297	T	0.78	0.79	0.014	0.003	-0.029	0.028	
rs114600294	G	0.79	0.80	-0.016	0.003	0.012	0.031	
rs1156541	C	0.22	0.23	0.015	0.003	-0.001	0.027	
rs11654952	T	0.86	0.86	-0.017	0.003	0.002	0.025	
rs11657730	C	0.64	0.64	0.013	0.002	0.014	0.024	
rs11689199	A	0.60	0.60	0.019	0.002	-0.019	0.018	
rs11714337	G	0.57	0.56	0.014	0.002	-0.018	0.023	
rs11763734	A	0.51	0.52	-0.013	0.002	0.016	0.019	
rs11810109	A	0.70	0.69	0.016	0.002	-0.047	0.020	
rs12105701	C	0.40	0.40	-0.013	0.002	-0.007	0.023	
rs12272012	G	0.96	0.96	-0.030	0.005	0.040	0.059	
rs12289262	C	0.73	0.73	-0.014	0.002	0.022	0.026	
rs1243182	C	0.69	0.67	-0.019	0.002	-0.010	0.019	
rs12491503	G	0.67	0.67	-0.014	0.002	0.019	0.018	
rs12541615	T	0.82	0.82	-0.018	0.003	0.010	0.025	
rs12554512	T	0.58	0.60	0.021	0.002	0.016	0.023	
rs12725114	G	0.80	0.80	0.015	0.003	0.015	0.027	
rs1278847	C	0.69	0.70	0.016	0.002	-0.003	0.020	
rs13029509	G	0.52	0.53	-0.018	0.002	0.006	0.022	
rs13107325	C	0.93	0.92	-0.029	0.004	0.031	0.032	
rs138256022	C	0.96	0.96	-0.031	0.006	-0.001	0.066	
rs1421334	A	0.45	0.44	0.017	0.002	0.021	0.020	
rs1451533	G	0.72	0.74	-0.016	0.002	-0.035	0.025	
rs17207890	G	0.66	0.65	0.016	0.002	0.021	0.019	
rs17379561	A	0.86	0.86	-0.026	0.003	-0.001	0.026	

rs17512836	T	0.97	0.97	0.042	0.007	0.060	0.049	
rs17727474	C	0.83	0.82	0.018	0.003	-0.024	0.035	
rs17789218	T	0.76	0.75	0.019	0.003	-0.011	0.026	
rs2034768	A	0.49	0.48	0.015	0.002	-0.001	0.019	
rs2045147	A	0.45	0.46	0.013	0.002	0.007	0.022	
rs2073869	C	0.83	0.83	0.019	0.003	-0.056	0.026	
rs2164744	T	0.64	0.61	-0.013	0.002	0.022	0.018	
rs2173650	G	0.85	0.84	0.018	0.003	0.048	0.031	
rs2184364	A	0.78	0.81	0.016	0.003	-0.006	0.025	rs12201987
rs2447098	C	0.48	0.48	-0.015	0.002	-0.026	0.019	
rs2460	G	0.74	0.75	-0.015	0.003	0.010	0.026	
rs2616830	G	0.46	0.44	0.017	0.002	-0.004	0.022	
rs262890	A	0.70	0.71	-0.019	0.002	-0.001	0.025	
rs2717559	A	0.56	0.55	0.012	0.002	-0.015	0.018	
rs2787374	T	0.41	0.43	0.013	0.002	-0.011	0.018	
rs303753	G	0.65	0.65	-0.015	0.002	-0.002	0.018	
rs34864022	A	0.93	0.94	-0.026	0.004	-0.010	0.048	
rs35574015	T	0.71	0.70	-0.013	0.002	0.015	0.019	
rs374722	G	0.15	0.15	0.025	0.003	0.032	0.033	
rs3754970	T	0.50	0.52	-0.015	0.002	-0.016	0.018	
rs3796386	G	0.57	0.58	-0.026	0.002	-0.013	0.017	
rs405797	T	0.25	0.26	-0.015	0.003	-0.016	0.026	rs178224
rs42210	G	0.29	0.29	-0.014	0.002	0.016	0.025	
rs4334769	G	0.47	0.48	0.012	0.002	0.018	0.022	
rs4382592	T	0.30	0.32	0.014	0.002	-0.032	0.024	

rs4523073	A	0.59	0.60	-0.014	0.002	0.003	0.023	
rs4577309	A	0.47	0.46	0.016	0.002	0.003	0.019	
rs4675246	G	0.80	0.80	-0.015	0.003	0.009	0.028	
rs4775373	T	0.36	0.36	0.013	0.002	-0.036	0.023	
rs4845364	A	0.50	0.49	-0.015	0.002	0.001	0.018	
rs4937842	G	0.63	0.62	-0.013	0.002	0.058	0.018	
rs4973576	C	0.30	0.30	-0.015	0.002	0.007	0.024	
rs55700114	G	0.71	0.69	-0.014	0.002	0.017	0.019	
rs55909997	G	0.65	0.66	-0.014	0.002	0.020	0.020	rs2335077
rs56103247	C	0.94	0.95	0.030	0.005	0.037	0.045	
rs56858768	G	0.70	0.71	-0.015	0.002	0.012	0.025	
rs57585211	T	0.83	0.83	-0.017	0.003	-0.008	0.030	
rs6131281	C	0.60	0.59	0.016	0.002	-0.020	0.023	
rs6141814	C	0.61	0.63	-0.014	0.002	0.037	0.023	
rs62379379	G	0.93	0.93	-0.026	0.004	0.025	0.040	
rs62641636	A	0.69	0.68	0.014	0.002	0.036	0.019	
rs6472942	T	0.57	0.56	-0.013	0.002	0.018	0.019	
rs6673341	T	0.47	0.47	-0.015	0.002	0.017	0.018	
rs66852340	C	0.78	0.77	-0.018	0.003	0.005	0.023	rs7668784
rs6797840	A	0.46	0.46	-0.016	0.002	0.015	0.018	
rs6825241	C	0.54	0.55	-0.017	0.002	0.004	0.020	
rs6850494	A	0.62	0.61	-0.014	0.002	0.000	0.019	
rs6905544	A	0.40	0.40	-0.019	0.002	0.046	0.023	
rs6973656	A	0.60	0.60	-0.014	0.002	-0.008	0.018	
rs6996198	C	0.84	0.84	-0.016	0.003	-0.041	0.024	

rs7089973	C	0.62	0.64	-0.013	0.002	0.039	0.019	
rs7157001	A	0.74	0.75	-0.014	0.003	0.021	0.028	
rs71658797	T	0.88	0.89	-0.020	0.003	0.005	0.032	
rs7184800	G	0.70	0.68	0.017	0.002	0.006	0.024	
rs7189927	T	0.36	0.33	0.015	0.002	-0.065	0.018	
rs7248205	C	0.40	0.41	0.014	0.002	0.029	0.018	
rs72671494	T	0.86	0.87	-0.017	0.003	0.029	0.034	
rs72781699	G	0.80	0.80	-0.019	0.003	0.047	0.023	
rs72828890	C	0.87	0.88	0.019	0.003	-0.024	0.037	
rs72834698	G	0.86	0.86	0.023	0.003	-0.005	0.026	
rs749671	G	0.63	0.59	0.016	0.002	-0.084	0.018	
rs7564130	T	0.64	0.63	-0.015	0.002	0.010	0.023	
rs7693082	G	0.30	0.30	0.015	0.002	-0.006	0.021	
rs7693703	G	0.91	0.91	0.023	0.004	0.036	0.040	
rs7700107	A	0.86	0.86	-0.021	0.003	0.077	0.026	
rs7716447	A	0.64	0.63	-0.013	0.002	0.001	0.017	rs16867703
rs77215114	A	0.93	0.93	0.024	0.004	0.009	0.047	
rs7834121	G	0.50	0.48	-0.014	0.002	0.010	0.022	rs7821826
rs7991062	C	0.66	0.67	-0.018	0.002	-0.016	0.024	rs7335993
rs801733	A	0.64	0.65	0.017	0.002	-0.019	0.019	
rs8043253	C	0.57	0.57	-0.012	0.002	0.012	0.018	
rs8756	C	0.48	0.47	-0.014	0.002	0.030	0.017	
rs9471333	C	0.45	0.44	0.013	0.002	0.010	0.022	
rs9563168	G	0.79	0.78	0.018	0.003	0.002	0.024	
rs9569734	A	0.84	0.88	0.019	0.003	0.040	0.035	rs4886037

rs9718104	T	0.94	0.94	-0.041	0.005	-0.047	0.048	
rs973734	C	0.15	0.15	0.017	0.003	0.032	0.031	
rs9834970	T	0.50	0.50	0.013	0.002	0.009	0.017	
rs984409	G	0.36	0.36	-0.015	0.002	-0.003	0.020	
rs9867121	C	0.82	0.81	0.020	0.003	-0.031	0.029	
rs9902312	T	0.68	0.68	0.015	0.002	0.026	0.023	
rs9964724	C	0.32	0.31	0.018	0.002	0.007	0.024	

Supplementary Table 13. Summary data for the genetic association between television watching and cognitive performance.

SNP	effect_allele	eaf.exposure	eaf.outcome	beta.exposure	se.exposure	beta.outcome	se.outcome	proxy
rs10041724	T	0.81	0.80	0.018	0.003	-0.012	0.004	
rs10054327	G	0.58	0.55	0.017	0.002	0.004	0.003	
rs10145592	C	0.41	0.39	-0.015	0.002	0.005	0.003	
rs10189857	A	0.57	0.58	-0.021	0.002	0.023	0.003	
rs1022785	G	0.14	0.15	0.018	0.003	-0.019	0.004	
rs10234444	G	0.82	0.80	0.016	0.003	-0.001	0.004	
rs10246289	A	0.11	0.12	0.019	0.003	-0.006	0.004	
rs1031423	T	0.22	0.21	-0.019	0.003	0.004	0.003	
rs10427502	G	0.62	0.65	0.014	0.002	-0.015	0.003	
rs10737620	T	0.27	0.30	0.014	0.002	-0.013	0.003	
rs10771746	C	0.72	0.73	-0.014	0.002	0.006	0.003	
rs10772643	C	0.11	0.11	0.025	0.004	0.000	0.005	
rs10876864	G	0.43	0.42	-0.013	0.002	0.017	0.003	
rs10890123	C	0.77	0.75	0.014	0.003	-0.006	0.003	
rs10932837	C	0.49	0.51	-0.013	0.002	0.000	0.003	
rs10940659	A	0.53	0.53	-0.013	0.002	0.007	0.003	
rs10994943	T	0.58	0.60	0.013	0.002	0.001	0.003	
rs11020045	A	0.67	0.67	-0.013	0.002	-0.002	0.003	rs1674086
rs11130793	C	0.60	0.63	0.013	0.002	-0.009	0.003	
rs111901094	G	0.82	0.88	-0.017	0.003	0.007	0.004	rs10415849
rs11201422	T	0.67	0.67	0.013	0.002	-0.001	0.003	
rs11218575	C	0.57	0.54	0.015	0.002	-0.001	0.003	
rs11245482	T	0.61	0.58	-0.013	0.002	0.003	0.003	

rs114328297	T	0.78	0.78	0.014	0.003	-0.007	0.003	
rs114600294	G	0.79	0.78	-0.016	0.003	-0.002	0.004	
rs1156541	C	0.22	0.22	0.015	0.003	-0.003	0.003	
rs11654952	T	0.86	0.89	-0.017	0.003	-0.001	0.004	
rs11657730	C	0.64	0.66	0.013	0.002	-0.011	0.003	
rs11689199	A	0.60	0.59	0.019	0.002	-0.022	0.003	
rs11714337	G	0.57	0.55	0.014	0.002	-0.016	0.003	
rs11763734	A	0.51	0.51	-0.013	0.002	0.001	0.003	
rs11810109	A	0.70	0.72	0.016	0.002	-0.017	0.003	
rs12105701	C	0.40	0.40	-0.013	0.002	0.012	0.003	
rs12272012	G	0.96	0.95	-0.030	0.005	0.011	0.007	
rs12289262	C	0.73	0.73	-0.014	0.002	0.011	0.003	
rs1243182	C	0.69	0.67	-0.019	0.002	0.009	0.003	
rs12476388	C	0.71	0.70	0.013	0.002	0.003	0.003	rs35094031
rs12491503	G	0.67	0.67	-0.014	0.002	0.006	0.003	
rs12541615	T	0.82	0.81	-0.018	0.003	0.018	0.004	
rs12554512	T	0.58	0.56	0.021	0.002	-0.027	0.003	
rs12725114	G	0.80	0.78	0.015	0.003	0.005	0.004	
rs1278847	C	0.69	0.70	0.016	0.002	-0.022	0.003	
rs13029509	G	0.52	0.53	-0.018	0.002	0.006	0.003	
rs13107325	C	0.93	0.91	-0.029	0.004	0.054	0.005	
rs138256022	C	0.96	0.96	-0.031	0.006	0.002	0.007	
rs1421334	A	0.45	0.41	0.017	0.002	-0.015	0.003	
rs1451533	G	0.72	0.77	-0.016	0.002	-0.002	0.003	
rs17207890	G	0.66	0.65	0.016	0.002	-0.017	0.003	

rs17379561	A	0.86	0.88	-0.026	0.003	0.005	0.004	
rs17512836	T	0.97	0.97	0.042	0.007	-0.006	0.009	
rs17727474	C	0.83	0.82	0.018	0.003	-0.007	0.004	
rs17789218	T	0.76	0.76	0.019	0.003	-0.005	0.003	
rs2034768	A	0.49	0.46	0.015	0.002	-0.007	0.003	
rs2045147	A	0.45	0.44	0.013	0.002	0.001	0.003	
rs2073869	C	0.83	0.84	0.019	0.003	-0.012	0.004	
rs2092829	G	0.71	0.74	0.014	0.002	0.011	0.003	rs2413032
rs2164744	T	0.64	0.61	-0.013	0.002	-0.004	0.003	
rs2173650	G	0.85	0.83	0.018	0.003	0.007	0.004	
rs2184364	A	0.78	0.82	0.016	0.003	0.001	0.004	rs12210048
rs2447098	C	0.48	0.48	-0.015	0.002	0.004	0.003	
rs2460	G	0.74	0.75	-0.015	0.003	0.009	0.003	
rs2616830	G	0.46	0.40	0.017	0.002	-0.002	0.003	
rs262890	A	0.70	0.71	-0.019	0.002	0.016	0.003	
rs2717559	A	0.56	0.55	0.012	0.002	-0.005	0.003	
rs2787374	T	0.41	0.44	0.013	0.002	0.004	0.003	
rs303753	G	0.65	0.68	-0.015	0.002	0.009	0.003	
rs34864022	A	0.93	0.93	-0.026	0.004	0.010	0.006	
rs35574015	T	0.71	0.71	-0.013	0.002	0.002	0.003	
rs374722	G	0.15	0.15	0.025	0.003	-0.003	0.004	
rs3754970	T	0.50	0.51	-0.015	0.002	0.018	0.003	
rs3796386	G	0.57	0.54	-0.026	0.002	0.025	0.003	
rs405797	T	0.25	0.27	-0.015	0.003	0.019	0.003	rs1268446
rs42210	G	0.29	0.29	-0.014	0.002	0.004	0.003	

rs4334769	G	0.47	0.51	0.012	0.002	-0.001	0.003	
rs4382592	T	0.30	0.30	0.014	0.002	-0.014	0.003	
rs4523073	A	0.59	0.58	-0.014	0.002	0.008	0.003	
rs4577309	A	0.47	0.46	0.016	0.002	0.001	0.003	
rs4675246	G	0.80	0.81	-0.015	0.003	0.009	0.004	
rs4775373	T	0.36	0.37	0.013	0.002	-0.007	0.003	
rs4845364	A	0.50	0.51	-0.015	0.002	0.012	0.003	
rs4937842	G	0.63	0.60	-0.013	0.002	-0.002	0.003	
rs4973576	C	0.30	0.34	-0.015	0.002	-0.002	0.003	
rs55700114	G	0.71	0.72	-0.014	0.002	0.009	0.003	
rs55909997	G	0.65	0.65	-0.014	0.002	0.004	0.003	rs12408634
rs56103247	C	0.94	0.95	0.030	0.005	-0.004	0.006	
rs56858768	G	0.70	0.70	-0.015	0.002	0.005	0.003	
rs57585211	T	0.83	0.84	-0.017	0.003	0.018	0.004	
rs6131281	C	0.60	0.57	0.016	0.002	-0.011	0.003	
rs6141814	C	0.61	0.65	-0.014	0.002	0.011	0.003	
rs62379379	G	0.93	0.93	-0.026	0.004	0.021	0.006	
rs62641636	A	0.69	0.73	0.014	0.002	-0.016	0.003	
rs6472942	T	0.57	0.55	-0.013	0.002	-0.001	0.003	
rs6673341	T	0.47	0.46	-0.015	0.002	0.006	0.003	
rs66852340	C	0.78	0.78	-0.018	0.003	0.011	0.003	rs6829279
rs6797840	A	0.46	0.48	-0.016	0.002	0.008	0.003	
rs6825241	C	0.54	0.54	-0.017	0.002	0.016	0.003	
rs6850494	A	0.62	0.61	-0.014	0.002	0.008	0.003	
rs6905544	A	0.40	0.42	-0.019	0.002	0.029	0.003	

rs6973656	A	0.60	0.54	-0.014	0.002	0.005	0.003	
rs6996198	C	0.84	0.81	-0.016	0.003	-0.003	0.004	
rs7089973	C	0.62	0.61	-0.013	0.002	0.003	0.003	
rs7157001	A	0.74	0.77	-0.014	0.003	0.006	0.003	
rs71658797	T	0.88	0.90	-0.020	0.003	-0.005	0.004	
rs7184800	G	0.70	0.68	0.017	0.002	-0.025	0.003	
rs7189927	T	0.36	0.32	0.015	0.002	-0.024	0.003	
rs7248205	C	0.40	0.43	0.014	0.002	0.006	0.003	
rs72671494	T	0.86	0.86	-0.017	0.003	0.001	0.004	
rs72781699	G	0.80	0.79	-0.019	0.003	-0.001	0.004	
rs72828890	C	0.87	0.88	0.019	0.003	-0.011	0.004	
rs72834698	G	0.86	0.84	0.023	0.003	0.002	0.004	
rs749671	G	0.63	0.62	0.016	0.002	0.005	0.003	
rs7564130	T	0.64	0.62	-0.015	0.002	0.003	0.003	
rs7693082	G	0.30	0.31	0.015	0.002	-0.011	0.003	
rs7693703	G	0.91	0.91	0.023	0.004	-0.007	0.005	
rs7700107	A	0.86	0.89	-0.021	0.003	0.025	0.004	
rs7716447	A	0.64	0.62	-0.013	0.002	0.011	0.003	rs4382197
rs77215114	A	0.93	0.94	0.024	0.004	-0.011	0.006	
rs7834121	G	0.50	0.52	-0.014	0.002	0.006	0.003	rs6997839
rs7991062	C	0.66	0.65	-0.018	0.002	0.001	0.003	rs7335993
rs801733	A	0.64	0.64	0.017	0.002	-0.003	0.003	
rs8043253	C	0.57	0.56	-0.012	0.002	-0.006	0.003	
rs8756	C	0.48	0.45	-0.014	0.002	0.005	0.003	
rs9471333	C	0.45	0.42	0.013	0.002	-0.008	0.003	

rs9563168	G	0.79	0.77	0.018	0.003	-0.002	0.004	
rs9569734	A	0.84	0.88	0.019	0.003	-0.003	0.004	rs4886037
rs9718104	T	0.94	0.94	-0.041	0.005	-0.007	0.006	
rs973734	C	0.15	0.12	0.017	0.003	-0.002	0.004	
rs9834970	T	0.50	0.45	0.013	0.002	-0.004	0.003	
rs984409	G	0.36	0.37	-0.015	0.002	0.008	0.003	
rs9867121	C	0.82	0.83	0.020	0.003	0.003	0.004	
rs9902312	T	0.68	0.66	0.015	0.002	0.001	0.003	
rs9964724	C	0.32	0.34	0.018	0.002	-0.007	0.003	

Supplementary Table 14. Summary data for the genetic association between computer use and cognitive performance.

SNP	effect_allele	eaf.exposure	eaf.outcome	beta.exposure	se.exposure	beta.outcome	se.outcome	proxy
rs10208088	C	0.42	0.43	0.013	0.002	0.000	0.003	
rs10754920	C	0.11	0.15	0.020	0.004	0.007	0.004	
rs113851275	G	0.89	0.89	-0.021	0.004	-0.016	0.005	
rs11749912	A	0.42	0.43	0.014	0.002	0.020	0.003	
rs12145677	G	0.70	0.70	-0.017	0.002	-0.022	0.003	
rs12603813	T	0.75	0.71	0.014	0.003	0.008	0.003	
rs12706626	G	0.62	0.59	-0.013	0.002	-0.007	0.003	
rs13262595	A	0.44	0.44	-0.016	0.002	-0.020	0.003	
rs136553	C	0.62	0.61	-0.015	0.002	-0.016	0.003	
rs1448355	C	0.38	0.38	-0.015	0.002	-0.008	0.003	
rs1469249	G	0.79	0.78	0.016	0.003	0.010	0.004	
rs162894	T	0.33	0.33	0.013	0.002	0.001	0.003	
rs166835	C	0.44	0.43	0.013	0.002	0.004	0.003	
rs198262	C	0.04	0.04	0.030	0.006	0.006	0.007	
rs2068625	T	0.30	0.32	-0.016	0.002	-0.011	0.003	
rs206965	T	0.21	0.25	0.016	0.003	0.009	0.004	
rs2220599	C	0.63	0.64	-0.016	0.002	0.003	0.003	
rs2345941	A	0.55	0.55	0.015	0.002	0.019	0.003	
rs2734849	A	0.49	0.50	-0.014	0.002	-0.009	0.003	
rs2748985	T	0.45	0.47	-0.015	0.002	0.006	0.003	
rs35933007	G	0.77	0.80	-0.015	0.003	-0.008	0.003	
rs3730399	A	0.93	0.95	0.025	0.005	0.013	0.006	
rs3944151	A	0.28	0.28	0.014	0.003	0.011	0.003	

rs4702	G	0.44	0.41	0.012	0.002	-0.003	0.003	
rs4977839	G	0.58	0.56	-0.020	0.002	-0.026	0.003	
rs55772938	A	0.70	0.70	-0.015	0.002	-0.014	0.003	
rs6129084	A	0.36	0.35	-0.014	0.002	-0.010	0.003	
rs6498759	T	0.31	0.31	0.013	0.002	-0.002	0.003	
rs66643547	C	0.65	0.66	-0.015	0.002	-0.011	0.003	
rs6744254	C	0.47	0.50	-0.016	0.002	-0.016	0.003	
rs6774533	C	0.29	0.31	-0.015	0.003	-0.006	0.003	
rs6857629	G	0.76	0.77	0.015	0.003	0.009	0.003	
rs6935828	C	0.45	0.43	-0.012	0.002	-0.008	0.003	
rs7020477	A	0.73	0.73	0.014	0.003	0.000	0.003	
rs7209653	T	0.70	0.69	0.016	0.002	0.005	0.003	
rs7288455	A	0.44	0.40	0.013	0.002	0.013	0.003	
rs73578186	C	0.68	0.67	0.015	0.002	0.009	0.003	
rs78082503	G	0.79	0.81	-0.015	0.003	-0.001	0.003	
rs784256	G	0.19	0.17	0.019	0.003	0.012	0.004	
rs9372625	G	0.62	0.59	-0.018	0.002	-0.026	0.003	
rs9477970	T	0.81	0.85	-0.016	0.003	-0.014	0.004	

Supplementary Table 15. The information of cohorts used in the genome-wide association study for stroke from MEGASTROKE consortium.

Cohort	AS	AIS	LAS	CES	SVS	Controls
CHARGE	4,348	3,028		602		80,613
METASTROKE	10,307	10,307	1,817	1,859	1,349	19,326
SIGN	7,743	7,743	1,253	2,135	1,534	17,970
DECODE1	5,520	4,483	512	1,346	615	2,552,131
EPIC-CVD	4,347	2,226				7,897
Young Lacunar DNA	1,403	1,268			1,012	970
SIFAP	981	981	184	170	104	1,824
INTERSTROKE EUR	911	826	188	208	243	863
HVH1	681	577	62	92	175	1,331
Glasgow	599	599	72	105	137	1,775
CADISP	555	555	67	211	31	9,259
Barcelona	520	520	119	215		315
FINLAND	501	501	99	187	62	1,813
SAHLSIS	298	298		35	85	596
MDC	202	202				4,295
HVH2	124	103		28	39	570
ICH	1,545					1,481

AS, all-cause stroke; AIS, all-cause ischemic stroke; LAS, large-artery atherosclerotic stroke; CES, cardioembolic stroke; SVS, small-vessel stroke.

Supplementary Table 16. The information of cohorts used in the genome-wide association study for intracerebral hemorrhage from ISGC consortium.

Cohort	Cases	Controls
GOCHA	316	457
GERFHS I, II	797	539
ISGC European Centers	482	485
HM-ICH	109	79
JUHSS	131	163
Lund Stroke Register	130	153
VHH-ICH	112	90

Supplementary Table 17. The information of cohorts used in the genome-wide association study for Alzheimer's disease from IGAP consortium.

Consortium		AD cases	Controls
ADGC	ACT	532	1,571
	ADC1	1,549	512
	ADC2	727	156
	ADC3	894	586
	ADC4	304	377
	ADC5	286	505
	ADC6	213	338
	ADNI	268	173
	BIOCARD	6	112
	CHAP	27	144
	EAS	9	141
	GenADA	666	712
	MAYO	658	1,046
	MIRAGE	491	738
	MTC	256	189
	NIALOAD	1,798	1,568
	NBB	80	48
	OHSU	132	153
	PFIZER	696	762
	RMAYO	13	233
	ROSMAP	295	769
	ROSMAP2	59	217
	TARC1	323	181
	TGEN2	668	365
	UKS	596	170
	UMCWRMSSM	1,177	1,126
	UPITT	1,255	829
	WASHU	339	187
	WASHU2	38	94
	WHICAP	73	560
CHARGE	AGES	95	2,708
	ASPS	277	169
	CHS	450	1,702
	FHS	330	3,910
	ROTTERDAM	985	4,985
EADI		2,240	6,631
GERAD	MRC	1,008	873
	ARUK	939	82
	BONN	551	37

	WASHU	423	156
	NIMH	127	-
	UCL:PRION	82	-
	UCL:LASER	47	-
	1958BC	-	5,342
	KORA	-	434
	HNR	-	353
	MAYO1	-	-
TOTALS		21,982	41,944

Supplementary Table 18. The information of cohorts used in the genome-wide association study for Parkinson's disease from PDGene consortium.

Cohort	Cases	Controls
IPDGC-DC	604	4916
IPDGC-FR	985	1984
IPDGC-GE	667	937
IPDGC-NE	744	2019
IPDGC-NIA	937	1896
IPDGC-UK	1705	5200
23andMe.v2	3261	29499
23andMe.v3	866	32538
Ash Jewish	268	178
HIHG	574	619
NGRC	1956	1982
PGPD	828	852
CHARGE-CHS	107	3164
CHARGE-FHS	60	3889
CHARGE-RSI	146	5609
Discovery	13708	95282
IPDGC-FR	553	474
IPDGC-GE	1044	871
IPDGC-GK	944	877
IPDGC-NIA	2407	2782
IPDGC-UK	405	547
Replication	5353	5551

Reference

1. Willer CJ, Schmidt EM, Sengupta S, et al. Discovery and refinement of loci associated with lipid levels. Nat. Genet. 2013;45(11):1274-1283
2. Dupuis J, Langenberg C, Prokopenko I, et al. New genetic loci implicated in fasting glucose homeostasis and their impact on type 2 diabetes risk. Nat. Genet. 2010;42(2):105-16
3. Wheeler E, Leong A, Liu CT, et al. Impact of common genetic determinants of Hemoglobin A1c on type 2 diabetes risk and diagnosis in ancestrally diverse populations: A transethnic genome-wide meta-analysis. PLoS Med. 2017;14(9):e1002383
4. Walford GA, Gustafsson S, Rybin D, et al. Genome-wide association study of the modified stumvoll insulin sensitivity index identifies BCL2 and FAM19A2 as novel insulin sensitivity loci. Diabetes 2016;65(10):3200-11
5. Evangelou E, Warren HR, Mosen-Ansorena D, et al. Genetic analysis of over 1 million people identifies 535 new loci associated with blood pressure traits. Nat. Genet. 2018;50(10):1412-1425
6. Pulit SL, Stoneman C, Morris AP, et al. Meta-Analysis of genome-wide association studies for body fat distribution in 694 649 individuals of European ancestry. Hum. Mol. Genet. 2019;28(1):166-174
7. Liu M, Jiang Y, Wedow R, et al. Association studies of up to 1.2 million individuals yield new insights into the genetic etiology of tobacco and alcohol use. Nat. Genet. 2019;51(2):237-244
8. Doherty A, Smith-Byrne K, Ferreira T, et al. GWAS identifies 14 loci for device-measured physical activity and sleep duration. Nat. Commun. 2018;9(1):5257

Supplementary Table 19. Summary data for the genetic association between television watching and outcomes.

SNP	EA	EAF	GX	GX_SE	GY	GY_SE
**CES**						
rs10041724	T	0.81	0.018	0.003	0.024	0.025
rs10054327	G	0.58	0.017	0.002	-0.021	0.019
rs10145592	C	0.41	-0.015	0.002	-0.010	0.020
rs10189857	A	0.57	-0.020	0.002	0.015	0.019
rs1022785	G	0.14	0.018	0.003	0.032	0.028
rs10234444	G	0.82	0.016	0.003	0.026	0.026
rs10246289	A	0.11	0.019	0.003	0.025	0.030
rs1031423	T	0.22	-0.019	0.003	0.073	0.024
rs10427502	G	0.62	0.014	0.002	-0.019	0.020
rs10737620	T	0.27	0.014	0.002	-0.019	0.022
rs10771746	C	0.72	-0.014	0.002	-0.023	0.021
rs10772643	C	0.11	0.025	0.003	-0.018	0.031
rs10876864	G	0.43	-0.013	0.002	-0.013	0.019
rs10890123	C	0.77	0.014	0.003	0.006	0.024
rs10932837	C	0.49	-0.013	0.002	-0.026	0.019
rs10940659	A	0.53	-0.013	0.002	0.010	0.019
rs10994943	T	0.58	0.013	0.002	-0.018	0.019
rs11020045	A	0.67	-0.013	0.002	-0.060	0.020
rs11130793	C	0.60	0.013	0.002	0.015	0.020
rs11201422	T	0.67	0.013	0.002	0.013	0.021
rs11218575	C	0.57	0.015	0.002	0.029	0.020
rs11245482	T	0.61	-0.013	0.002	-0.014	0.019
rs114328297	T	0.78	0.014	0.003	-0.017	0.025
rs114600294	G	0.79	-0.016	0.003	0.039	0.031
rs1156541	C	0.22	0.014	0.003	0.025	0.023
rs11654952	T	0.86	-0.017	0.003	-0.044	0.029
rs11657730	C	0.64	0.013	0.002	-0.026	0.023
rs11689199	A	0.60	0.018	0.002	-0.026	0.020
rs11714337	G	0.57	0.014	0.002	-0.002	0.020
rs11763734	A	0.51	-0.012	0.002	0.009	0.018
rs11810109	A	0.70	0.016	0.002	0.009	0.021
rs12105701	C	0.40	-0.013	0.002	-0.024	0.020
rs12272012	G	0.96	-0.030	0.005	0.082	0.053
rs12289262	C	0.73	-0.014	0.002	0.012	0.022
rs1243182	C	0.69	-0.019	0.002	0.022	0.021
rs12476388	C	0.71	0.013	0.002	-0.021	0.022
rs12491503	G	0.67	-0.014	0.002	-0.020	0.021
rs12541615	T	0.82	-0.018	0.003	-0.022	0.025
rs12554512	T	0.58	0.021	0.002	0.005	0.020
rs12725114	G	0.80	0.015	0.003	-0.022	0.024
rs1278847	C	0.69	0.016	0.002	-0.008	0.022
rs13029509	G	0.52	-0.018	0.002	0.014	0.019

rs13107325	C	0.93	-0.029	0.004	0.030	0.044
rs138256022	C	0.96	-0.031	0.006	0.054	0.057
rs1421334	A	0.45	0.017	0.002	0.014	0.020
rs1451533	G	0.72	-0.016	0.002	-0.001	0.022
rs17207890	G	0.66	0.016	0.002	0.007	0.020
rs17379561	A	0.86	-0.025	0.003	-0.006	0.027
rs17512836	T	0.97	0.042	0.007	0.040	0.061
rs17727474	C	0.83	0.018	0.003	0.004	0.025
rs17789218	T	0.76	0.019	0.003	-0.046	0.024
rs2034768	A	0.49	0.015	0.002	0.003	0.019
rs2045147	A	0.45	0.013	0.002	0.002	0.019
rs2073869	C	0.83	0.019	0.003	-0.027	0.025
rs2092829	G	0.71	0.014	0.002	-0.036	0.021
rs2164744	T	0.64	-0.013	0.002	0.014	0.020
rs2173650	G	0.85	0.018	0.003	-0.032	0.027
rs2184364	A	0.78	0.016	0.003	-0.037	0.026
rs2447098	C	0.48	-0.015	0.002	0.010	0.019
rs2460	G	0.74	-0.015	0.002	0.009	0.023
rs2584597	T	0.66	0.015	0.002	0.023	0.022
rs2616830	G	0.46	0.016	0.002	-0.002	0.019
rs262890	A	0.70	-0.019	0.002	-0.034	0.021
rs2717559	A	0.56	0.012	0.002	0.012	0.020
rs2787374	T	0.41	0.012	0.002	0.038	0.019
rs303753	G	0.65	-0.014	0.002	-0.028	0.020
rs34864022	A	0.93	-0.026	0.004	-0.019	0.037
rs35574015	T	0.71	-0.013	0.002	0.036	0.021
rs374722	G	0.15	0.024	0.003	-0.009	0.027
rs3754970	T	0.50	-0.015	0.002	0.042	0.019
rs3796386	G	0.57	-0.026	0.002	-0.012	0.020
rs405797	T	0.25	-0.015	0.003	-0.020	0.023
rs42210	G	0.29	-0.014	0.002	-0.017	0.023
rs4334769	G	0.47	0.012	0.002	0.015	0.019
rs4382592	T	0.30	0.014	0.002	0.023	0.021
rs4523073	A	0.59	-0.014	0.002	0.009	0.020
rs4577309	A	0.47	0.016	0.002	-0.022	0.019
rs4675246	G	0.80	-0.015	0.003	-0.015	0.024
rs4775373	T	0.36	0.013	0.002	0.021	0.020
rs4845364	A	0.50	-0.015	0.002	0.005	0.019
rs4937842	G	0.63	-0.013	0.002	-0.026	0.021
rs4973576	C	0.30	-0.015	0.002	0.023	0.021
rs55700114	G	0.71	-0.014	0.002	-0.006	0.021
rs55909997	G	0.65	-0.014	0.002	0.008	0.020
rs56103247	C	0.94	0.030	0.005	0.084	0.051
rs56858768	G	0.70	-0.015	0.002	0.012	0.021
rs57585211	T	0.83	-0.017	0.003	-0.026	0.026
rs6131281	C	0.60	0.016	0.002	-0.061	0.019
rs6141814	C	0.61	-0.014	0.002	0.034	0.020

rs62379379	G	0.93	-0.026	0.004	-0.027	0.037
rs62641636	A	0.69	0.014	0.002	0.033	0.022
rs6472942	T	0.57	-0.013	0.002	0.005	0.019
rs6673341	T	0.47	-0.015	0.002	0.023	0.019
rs66852340	C	0.78	-0.018	0.003	0.008	0.024
rs6721975	T	0.23	-0.017	0.003	-0.101	0.024
rs6797840	A	0.46	-0.016	0.002	0.004	0.020
rs6825241	C	0.54	-0.017	0.002	-0.005	0.019
rs6850494	A	0.62	-0.014	0.002	-0.034	0.020
rs6905544	A	0.40	-0.019	0.002	0.013	0.019
rs6973656	A	0.60	-0.013	0.002	-0.068	0.019
rs6996198	C	0.84	-0.016	0.003	-0.010	0.027
rs7089973	C	0.62	-0.013	0.002	0.022	0.020
rs7157001	A	0.74	-0.014	0.002	0.023	0.025
rs71658797	T	0.88	-0.020	0.003	0.019	0.031
rs7184800	G	0.70	0.017	0.002	-0.005	0.021
rs7189927	T	0.36	0.015	0.002	0.016	0.021
rs7248205	C	0.40	0.014	0.002	-0.061	0.021
rs72671494	T	0.86	-0.017	0.003	0.007	0.030
rs72781699	G	0.80	-0.019	0.003	0.000	0.024
rs72828890	C	0.87	0.019	0.003	-0.007	0.031
rs72834698	G	0.86	0.023	0.003	-0.036	0.028
rs749671	G	0.63	0.016	0.002	-0.019	0.020
rs7564130	T	0.64	-0.015	0.002	0.008	0.020
rs7693082	G	0.30	0.015	0.002	-0.014	0.020
rs7693703	G	0.91	0.023	0.004	0.077	0.035
rs7700107	A	0.86	-0.021	0.003	-0.013	0.029
rs7716447	A	0.64	-0.013	0.002	0.011	0.024
rs77215114	A	0.93	0.024	0.004	0.042	0.039
rs7991062	C	0.66	-0.018	0.002	0.008	0.021
rs801733	A	0.64	0.017	0.002	0.008	0.020
rs8043253	C	0.57	-0.012	0.002	-0.015	0.019
rs8756	C	0.48	-0.013	0.002	0.001	0.020
rs9471333	C	0.45	0.013	0.002	-0.010	0.019
rs9563168	G	0.79	0.018	0.003	-0.005	0.023
rs9569734	A	0.84	0.019	0.003	0.017	0.029
rs9718104	T	0.94	-0.041	0.005	-0.048	0.050
rs973734	C	0.15	0.017	0.003	0.007	0.027
rs9834970	T	0.50	0.013	0.002	0.012	0.019
rs984409	G	0.36	-0.015	0.002	0.030	0.020
rs9867121	C	0.82	0.019	0.003	-0.030	0.026
rs9902312	T	0.68	0.015	0.002	0.003	0.020
rs9964724	C	0.32	0.018	0.002	-0.015	0.020
**LAS**						
rs10041724	T	0.81	0.018	0.003	0.021	0.032
rs10054327	G	0.58	0.017	0.002	-0.009	0.025
rs10145592	C	0.41	-0.015	0.002	0.015	0.025

rs10189857	A	0.57	-0.020	0.002	0.013	0.025
rs1022785	G	0.14	0.018	0.003	0.057	0.035
rs10234444	G	0.82	0.016	0.003	0.074	0.034
rs10246289	A	0.11	0.019	0.003	0.087	0.038
rs1031423	T	0.22	-0.019	0.003	0.049	0.030
rs10427502	G	0.62	0.014	0.002	0.056	0.026
rs10737620	T	0.27	0.014	0.002	-0.019	0.028
rs10771746	C	0.72	-0.014	0.002	0.028	0.027
rs10772643	C	0.11	0.025	0.003	0.080	0.039
rs10876864	G	0.43	-0.013	0.002	-0.021	0.025
rs10890123	C	0.77	0.014	0.003	-0.023	0.030
rs10932837	C	0.49	-0.013	0.002	0.014	0.025
rs10940659	A	0.53	-0.013	0.002	0.023	0.024
rs10994943	T	0.58	0.013	0.002	0.037	0.025
rs11020045	A	0.67	-0.013	0.002	-0.092	0.026
rs11130793	C	0.60	0.013	0.002	0.012	0.026
rs11201422	T	0.67	0.013	0.002	0.028	0.029
rs11218575	C	0.57	0.015	0.002	-0.040	0.026
rs11245482	T	0.61	-0.013	0.002	-0.024	0.025
rs114328297	T	0.78	0.014	0.003	-0.017	0.032
rs114600294	G	0.79	-0.016	0.003	-0.011	0.040
rs1156541	C	0.22	0.014	0.003	-0.041	0.030
rs11654952	T	0.86	-0.017	0.003	-0.107	0.037
rs11657730	C	0.64	0.013	0.002	-0.015	0.030
rs11689199	A	0.60	0.018	0.002	0.003	0.026
rs11714337	G	0.57	0.014	0.002	0.024	0.026
rs11763734	A	0.51	-0.012	0.002	-0.030	0.025
rs11810109	A	0.70	0.016	0.002	0.036	0.027
rs12105701	C	0.40	-0.013	0.002	0.002	0.027
rs12272012	G	0.96	-0.030	0.005	-0.099	0.061
rs12289262	C	0.73	-0.014	0.002	-0.008	0.029
rs1243182	C	0.69	-0.019	0.002	0.011	0.027
rs12476388	C	0.71	0.013	0.002	-0.018	0.027
rs12491503	G	0.67	-0.014	0.002	0.009	0.027
rs12541615	T	0.82	-0.018	0.003	0.018	0.031
rs12554512	T	0.58	0.021	0.002	0.002	0.026
rs12725114	G	0.80	0.015	0.003	-0.010	0.030
rs1278847	C	0.69	0.016	0.002	-0.008	0.029
rs13029509	G	0.52	-0.018	0.002	-0.058	0.025
rs13107325	C	0.93	-0.029	0.004	-0.021	0.053
rs138256022	C	0.96	-0.031	0.006	0.010	0.072
rs1421334	A	0.45	0.017	0.002	-0.051	0.026
rs1451533	G	0.72	-0.016	0.002	0.009	0.029
rs17207890	G	0.66	0.016	0.002	0.017	0.026
rs17379561	A	0.86	-0.025	0.003	-0.006	0.034
rs17512836	T	0.97	0.042	0.007	0.103	0.087
rs17727474	C	0.83	0.018	0.003	-0.018	0.033

rs17789218	T	0.76	0.019	0.003	-0.041	0.031
rs2034768	A	0.49	0.015	0.002	-0.038	0.025
rs2045147	A	0.45	0.013	0.002	-0.010	0.025
rs2073869	C	0.83	0.019	0.003	0.007	0.032
rs2092829	G	0.71	0.014	0.002	-0.027	0.027
rs2164744	T	0.64	-0.013	0.002	-0.001	0.025
rs2173650	G	0.85	0.018	0.003	-0.051	0.035
rs2184364	A	0.78	0.016	0.003	0.002	0.032
rs2447098	C	0.48	-0.015	0.002	0.017	0.025
rs2460	G	0.74	-0.015	0.002	-0.044	0.029
rs2584597	T	0.66	0.015	0.002	0.005	0.028
rs2616830	G	0.46	0.016	0.002	-0.044	0.025
rs262890	A	0.70	-0.019	0.002	-0.004	0.030
rs2717559	A	0.56	0.012	0.002	-0.052	0.027
rs2787374	T	0.41	0.012	0.002	0.037	0.025
rs303753	G	0.65	-0.014	0.002	0.005	0.026
rs34864022	A	0.93	-0.026	0.004	-0.015	0.049
rs35574015	T	0.71	-0.013	0.002	-0.035	0.027
rs374722	G	0.15	0.024	0.003	0.040	0.034
rs3754970	T	0.50	-0.015	0.002	-0.025	0.025
rs3796386	G	0.57	-0.026	0.002	-0.058	0.026
rs405797	T	0.25	-0.015	0.003	-0.046	0.030
rs42210	G	0.29	-0.014	0.002	0.012	0.029
rs4334769	G	0.47	0.012	0.002	-0.017	0.026
rs4382592	T	0.30	0.014	0.002	0.010	0.027
rs4523073	A	0.59	-0.014	0.002	-0.037	0.026
rs4577309	A	0.47	0.016	0.002	0.020	0.025
rs4675246	G	0.80	-0.015	0.003	0.014	0.031
rs4775373	T	0.36	0.013	0.002	0.003	0.026
rs4845364	A	0.50	-0.015	0.002	0.010	0.024
rs4937842	G	0.63	-0.013	0.002	0.037	0.028
rs4973576	C	0.30	-0.015	0.002	0.004	0.027
rs55700114	G	0.71	-0.014	0.002	-0.006	0.026
rs55909997	G	0.65	-0.014	0.002	-0.014	0.026
rs56103247	C	0.94	0.030	0.005	0.019	0.070
rs56858768	G	0.70	-0.015	0.002	-0.036	0.028
rs57585211	T	0.83	-0.017	0.003	-0.012	0.034
rs6131281	C	0.60	0.016	0.002	-0.013	0.025
rs6141814	C	0.61	-0.014	0.002	-0.007	0.026
rs62379379	G	0.93	-0.026	0.004	-0.073	0.048
rs62641636	A	0.69	0.014	0.002	0.022	0.027
rs6472942	T	0.57	-0.013	0.002	0.008	0.025
rs6673341	T	0.47	-0.015	0.002	-0.029	0.025
rs66852340	C	0.78	-0.018	0.003	-0.043	0.030
rs6721975	T	0.23	-0.017	0.003	0.033	0.031
rs6797840	A	0.46	-0.016	0.002	-0.033	0.025
rs6825241	C	0.54	-0.017	0.002	-0.014	0.025

rs6850494	A	0.62	-0.014	0.002	-0.037	0.025
rs6905544	A	0.40	-0.019	0.002	0.007	0.025
rs6973656	A	0.60	-0.013	0.002	0.002	0.025
rs6996198	C	0.84	-0.016	0.003	0.003	0.034
rs7089973	C	0.62	-0.013	0.002	0.010	0.026
rs7157001	A	0.74	-0.014	0.002	0.055	0.034
rs71658797	T	0.88	-0.020	0.003	0.007	0.040
rs7184800	G	0.70	0.017	0.002	-0.002	0.027
rs7189927	T	0.36	0.015	0.002	-0.012	0.026
rs7248205	C	0.40	0.014	0.002	-0.068	0.027
rs72671494	T	0.86	-0.017	0.003	-0.016	0.038
rs72781699	G	0.80	-0.019	0.003	-0.053	0.030
rs72828890	C	0.87	0.019	0.003	-0.056	0.040
rs72834698	G	0.86	0.023	0.003	-0.022	0.037
rs749671	G	0.63	0.016	0.002	0.013	0.027
rs7564130	T	0.64	-0.015	0.002	0.007	0.026
rs7693082	G	0.30	0.015	0.002	-0.055	0.028
rs7693703	G	0.91	0.023	0.004	-0.042	0.043
rs7700107	A	0.86	-0.021	0.003	-0.053	0.036
rs7716447	A	0.64	-0.013	0.002	0.046	0.031
rs77215114	A	0.93	0.024	0.004	-0.054	0.051
rs7991062	C	0.66	-0.018	0.002	0.024	0.027
rs801733	A	0.64	0.017	0.002	-0.002	0.026
rs8043253	C	0.57	-0.012	0.002	-0.022	0.025
rs8756	C	0.48	-0.013	0.002	-0.066	0.027
rs9471333	C	0.45	0.013	0.002	0.006	0.025
rs9563168	G	0.79	0.018	0.003	0.011	0.030
rs9569734	A	0.84	0.019	0.003	-0.029	0.036
rs9718104	T	0.94	-0.041	0.005	-0.210	0.064
rs973734	C	0.15	0.017	0.003	0.020	0.035
rs9834970	T	0.50	0.013	0.002	0.003	0.025
rs984409	G	0.36	-0.015	0.002	-0.039	0.026
rs9867121	C	0.82	0.019	0.003	-0.043	0.032
rs9902312	T	0.68	0.015	0.002	0.020	0.026
rs9964724	C	0.32	0.018	0.002	0.013	0.027
**ICH**						
rs10041724	T	0.81	0.018	0.003	0.205	0.064
rs10189857	A	0.57	-0.020	0.002	-0.163	0.052
rs6141814	C	0.61	-0.014	0.002	0.123	0.053
rs11763734	A	0.51	-0.012	0.002	-0.113	0.051
rs255292	C	0.40	-0.012	0.002	-0.108	0.052
rs12272012	G	0.96	-0.030	0.005	0.274	0.134
rs10427502	G	0.62	0.014	0.002	-0.109	0.054
rs17727474	C	0.83	0.018	0.003	-0.129	0.067
rs6124476	T	0.59	0.012	0.002	0.099	0.052
rs6996198	C	0.84	-0.016	0.003	-0.128	0.069
rs2460	G	0.74	-0.015	0.002	-0.109	0.059

rs11201422	T	0.67	0.013	0.002	-0.094	0.055
rs262890	A	0.70	-0.019	0.002	-0.098	0.058
rs11125515	C	0.73	-0.014	0.002	-0.095	0.058
rs35574015	T	0.71	-0.013	0.002	0.084	0.057
rs11179838	C	0.64	0.013	0.002	-0.080	0.054
rs7788008	G	0.57	0.013	0.002	-0.075	0.051
rs3796386	G	0.57	-0.026	0.002	-0.076	0.052
rs55700114	G	0.71	-0.014	0.002	-0.080	0.055
rs328277	C	0.40	0.012	0.002	-0.074	0.052
rs17207890	G	0.66	0.016	0.002	-0.072	0.053
rs12491503	G	0.67	-0.014	0.002	-0.070	0.054
rs12632778	T	0.95	-0.028	0.005	0.162	0.124
rs7700107	A	0.86	-0.021	0.003	0.093	0.072
rs2787374	T	0.41	0.012	0.002	0.067	0.052
rs9563168	G	0.79	0.018	0.003	0.080	0.062
rs114328297	T	0.78	0.014	0.003	-0.082	0.065
rs6905544	A	0.40	-0.019	0.002	-0.066	0.052
rs6973656	A	0.60	-0.013	0.002	-0.064	0.052
rs4334769	G	0.47	0.012	0.002	-0.059	0.052
rs6031440	A	0.40	0.012	0.002	-0.060	0.053
rs7693082	G	0.30	0.015	0.002	0.061	0.055
rs10940659	A	0.53	-0.013	0.002	-0.057	0.052
rs7564130	T	0.64	-0.015	0.002	-0.057	0.053
rs11689199	A	0.60	0.018	0.002	0.056	0.052
rs10739499	C	0.34	0.014	0.002	0.057	0.055
rs66852340	C	0.78	-0.018	0.003	-0.064	0.063
rs2173650	G	0.85	0.018	0.003	-0.072	0.073
rs17512836	T	0.97	0.042	0.007	0.150	0.155
rs13029509	G	0.52	-0.018	0.002	0.050	0.052
rs10947452	T	0.35	0.014	0.002	0.052	0.055
rs2336895	G	0.36	0.012	0.002	0.049	0.054
rs10791546	C	0.59	0.012	0.002	0.046	0.052
rs10054327	G	0.58	0.017	0.002	-0.044	0.051
rs17379561	A	0.86	-0.025	0.003	0.063	0.075
rs11714337	G	0.57	0.014	0.002	-0.044	0.052
rs2291681	G	0.58	0.013	0.002	0.044	0.053
rs78394231	T	0.90	-0.021	0.004	-0.069	0.086
rs9834970	T	0.50	0.013	0.002	0.042	0.052
rs8043253	C	0.57	-0.012	0.002	0.042	0.052
rs11810109	A	0.70	0.016	0.002	0.045	0.057
rs1352890	C	0.48	-0.012	0.002	0.041	0.052
rs10145592	C	0.41	-0.015	0.002	-0.041	0.053
rs263771	C	0.77	-0.017	0.003	0.048	0.062
rs71658797	T	0.88	-0.020	0.003	-0.065	0.084
rs12342024	G	0.89	-0.021	0.003	0.066	0.085
rs7089973	C	0.62	-0.013	0.002	-0.041	0.054
rs9471333	C	0.45	0.013	0.002	0.039	0.051

rs9528023	C	0.72	0.013	0.002	0.043	0.058
rs4845364	A	0.50	-0.015	0.002	-0.038	0.052
rs62379379	G	0.93	-0.026	0.004	-0.071	0.101
rs749671	G	0.63	0.016	0.002	0.036	0.052
rs7609	A	0.23	0.015	0.003	-0.042	0.062
rs62332760	G	0.61	-0.014	0.002	-0.036	0.054
rs10246289	A	0.11	0.019	0.003	0.054	0.080
rs12725114	G	0.80	0.015	0.003	-0.043	0.063
rs2616830	G	0.46	0.016	0.002	0.033	0.050
rs801733	A	0.64	0.017	0.002	0.034	0.054
rs10890123	C	0.77	0.014	0.003	0.040	0.063
rs4577309	A	0.47	0.016	0.002	-0.031	0.051
rs72781699	G	0.80	-0.019	0.003	0.039	0.064
rs12105701	C	0.40	-0.013	0.002	-0.032	0.052
rs1156541	C	0.22	0.014	0.003	0.036	0.061
rs2034768	A	0.49	0.015	0.002	0.030	0.051
rs12476388	C	0.71	0.013	0.002	0.033	0.058
rs374722	G	0.15	0.024	0.003	0.039	0.071
rs1022785	G	0.14	0.018	0.003	0.038	0.073
rs6797840	A	0.46	-0.016	0.002	-0.027	0.052
rs77215114	A	0.93	0.024	0.004	0.052	0.101
rs984409	G	0.36	-0.015	0.002	0.025	0.053
rs1243182	C	0.69	-0.019	0.002	0.024	0.054
rs12289262	C	0.73	-0.014	0.002	-0.025	0.058
rs34864022	A	0.93	-0.026	0.004	0.044	0.111
rs11245482	T	0.61	-0.013	0.002	0.021	0.053
rs973734	C	0.15	0.017	0.003	-0.027	0.072
rs11130793	C	0.60	0.013	0.002	0.020	0.053
rs2694151	C	0.06	0.026	0.005	-0.040	0.109
rs112632642	G	0.93	-0.024	0.004	0.038	0.106
rs10737620	T	0.27	0.014	0.002	-0.020	0.057
rs10771746	C	0.72	-0.014	0.002	-0.019	0.057
rs10057545	G	0.56	-0.014	0.002	-0.016	0.051
rs56858768	G	0.70	-0.015	0.002	-0.018	0.057
rs7184800	G	0.70	0.017	0.002	0.017	0.055
rs2447098	C	0.48	-0.015	0.002	0.016	0.053
rs11218575	C	0.57	0.015	0.002	-0.016	0.052
rs17789218	T	0.76	0.019	0.003	0.018	0.060
rs9902312	T	0.68	0.015	0.002	-0.015	0.054
rs11896615	G	0.85	0.017	0.003	0.019	0.071
rs9569734	A	0.84	0.019	0.003	-0.020	0.073
rs6850494	A	0.62	-0.014	0.002	0.014	0.053
rs10772643	C	0.11	0.025	0.003	0.021	0.083
rs72725224	A	0.98	-0.046	0.008	-0.049	0.195
rs6825241	C	0.54	-0.017	0.002	-0.013	0.051
rs12554512	T	0.58	0.021	0.002	-0.013	0.054
rs9964724	C	0.32	0.018	0.002	-0.013	0.055

rs11020045	A	0.67	-0.013	0.002	-0.013	0.055
rs8756	C	0.48	-0.013	0.002	0.011	0.051
rs74996610	C	0.95	-0.031	0.005	-0.029	0.135
rs2791574	T	0.66	-0.013	0.002	0.011	0.054
rs6131281	C	0.60	0.016	0.002	-0.011	0.053
rs11629120	T	0.59	-0.013	0.002	-0.009	0.052
rs2971640	G	0.51	-0.013	0.002	0.009	0.051
rs2045147	A	0.45	0.013	0.002	-0.009	0.052
rs4775373	T	0.36	0.013	0.002	-0.008	0.053
rs57585211	T	0.83	-0.017	0.003	0.008	0.070
rs56398417	C	0.69	0.014	0.002	-0.006	0.057
rs12475168	G	0.87	-0.018	0.003	0.006	0.072
rs10234444	G	0.82	0.016	0.003	-0.005	0.070
rs631130	T	0.37	-0.014	0.002	-0.004	0.059
rs3137	G	0.85	0.017	0.003	-0.004	0.072
rs4675246	G	0.80	-0.015	0.003	0.003	0.066
rs1913808	G	0.71	0.015	0.002	-0.002	0.057
rs2164744	T	0.64	-0.013	0.002	0.002	0.052
rs1031423	T	0.22	-0.019	0.003	0.002	0.062
rs4382592	T	0.30	0.014	0.002	0.001	0.056
rs4523073	A	0.59	-0.014	0.002	0.001	0.053
rs6125906	C	0.91	-0.021	0.004	0.000	0.090
**nonlobar ICH**						
rs114328297	T	0.78	0.014	0.003	-0.178	0.076
rs17207890	G	0.66	0.016	0.002	-0.140	0.063
rs10041724	T	0.81	0.018	0.003	0.169	0.076
rs6141814	C	0.61	-0.014	0.002	0.117	0.063
rs3796386	G	0.57	-0.026	0.002	-0.112	0.062
rs2787374	T	0.41	0.012	0.002	0.111	0.061
rs34864022	A	0.93	-0.026	0.004	0.246	0.142
rs2173650	G	0.85	0.018	0.003	-0.146	0.085
rs17727474	C	0.83	0.018	0.003	-0.130	0.080
rs6124476	T	0.59	0.012	0.002	0.094	0.062
rs12272012	G	0.96	-0.030	0.005	0.242	0.158
rs7788008	G	0.57	0.013	0.002	-0.093	0.061
rs2336895	G	0.36	0.012	0.002	0.097	0.064
rs11763734	A	0.51	-0.012	0.002	-0.093	0.061
rs17512836	T	0.97	0.042	0.007	0.279	0.191
rs12491503	G	0.67	-0.014	0.002	-0.089	0.063
rs1352890	C	0.48	-0.012	0.002	0.086	0.062
rs255292	C	0.40	-0.012	0.002	-0.084	0.062
rs4577309	A	0.47	0.016	0.002	-0.082	0.060
rs2460	G	0.74	-0.015	0.002	-0.094	0.070
rs11125515	C	0.73	-0.014	0.002	-0.090	0.069
rs35574015	T	0.71	-0.013	0.002	0.085	0.068
rs7564130	T	0.64	-0.015	0.002	-0.078	0.063
rs7693082	G	0.30	0.015	0.002	0.078	0.066

rs4334769	G	0.47	0.012	0.002	-0.072	0.062
rs374722	G	0.15	0.024	0.003	0.097	0.084
rs66852340	C	0.78	-0.018	0.003	-0.084	0.074
rs10189857	A	0.57	-0.020	0.002	-0.069	0.061
rs749671	G	0.63	0.016	0.002	0.070	0.063
rs10054327	G	0.58	0.017	0.002	-0.067	0.061
rs10246289	A	0.11	0.019	0.003	0.103	0.094
rs6905544	A	0.40	-0.019	0.002	-0.067	0.062
rs2971640	G	0.51	-0.013	0.002	0.066	0.061
rs78394231	T	0.90	-0.021	0.004	-0.109	0.101
rs10739499	C	0.34	0.014	0.002	0.069	0.066
rs10791546	C	0.59	0.012	0.002	0.065	0.062
rs17379561	A	0.86	-0.025	0.003	0.090	0.089
rs10427502	G	0.62	0.014	0.002	-0.064	0.064
rs7700107	A	0.86	-0.021	0.003	0.084	0.085
rs2791574	T	0.66	-0.013	0.002	0.063	0.065
rs9563168	G	0.79	0.018	0.003	0.071	0.075
rs4675246	G	0.80	-0.015	0.003	0.075	0.079
rs11245482	T	0.61	-0.013	0.002	0.059	0.063
rs9528023	C	0.72	0.013	0.002	0.064	0.070
rs10057545	G	0.56	-0.014	0.002	-0.052	0.061
rs11689199	A	0.60	0.018	0.002	0.053	0.062
rs12554512	T	0.58	0.021	0.002	0.055	0.064
rs11917871	T	0.42	-0.017	0.002	-0.053	0.063
rs984409	G	0.36	-0.015	0.002	-0.052	0.064
rs328277	C	0.40	0.012	0.002	-0.050	0.062
rs1913808	G	0.71	0.015	0.002	-0.054	0.067
rs12476388	C	0.71	0.013	0.002	0.055	0.069
rs7089973	C	0.62	-0.013	0.002	-0.051	0.064
rs56398417	C	0.69	0.014	0.002	0.054	0.068
rs10737620	T	0.27	0.014	0.002	-0.052	0.067
rs7609	A	0.23	0.015	0.003	-0.053	0.074
rs10890123	C	0.77	0.014	0.003	0.053	0.076
rs55700114	G	0.71	-0.014	0.002	-0.046	0.066
rs6973656	A	0.60	-0.013	0.002	-0.043	0.062
rs12289262	C	0.73	-0.014	0.002	-0.047	0.069
rs11179838	C	0.64	0.013	0.002	-0.044	0.064
rs13029509	G	0.52	-0.018	0.002	0.041	0.062
rs12632778	T	0.95	-0.028	0.005	0.097	0.148
rs11810109	A	0.70	0.016	0.002	0.042	0.067
rs6996198	C	0.84	-0.016	0.003	-0.051	0.083
rs71658797	T	0.88	-0.020	0.003	-0.060	0.099
rs9569734	A	0.84	0.019	0.003	-0.052	0.086
rs4845364	A	0.50	-0.015	0.002	-0.037	0.062
rs262890	A	0.70	-0.019	0.002	-0.040	0.068
rs6031440	A	0.40	0.012	0.002	-0.037	0.062
rs12342024	G	0.89	-0.021	0.003	0.058	0.099

rs10940659	A	0.53	-0.013	0.002	-0.035	0.062
rs7184800	G	0.70	0.017	0.002	-0.037	0.065
rs8043253	C	0.57	-0.012	0.002	0.034	0.062
rs11629120	T	0.59	-0.013	0.002	-0.033	0.062
rs1156541	C	0.22	0.014	0.003	0.038	0.073
rs12105701	C	0.40	-0.013	0.002	-0.030	0.062
rs1031423	T	0.22	-0.019	0.003	0.034	0.074
rs9902312	T	0.68	0.015	0.002	-0.030	0.064
rs2694151	C	0.06	0.026	0.005	0.058	0.127
rs77215114	A	0.93	0.024	0.004	0.055	0.120
rs11201422	T	0.67	0.013	0.002	-0.030	0.066
rs72725224	A	0.98	-0.046	0.008	0.084	0.236
rs72781699	G	0.80	-0.019	0.003	-0.026	0.075
rs2447098	C	0.48	-0.015	0.002	0.022	0.063
rs9471333	C	0.45	0.013	0.002	-0.021	0.062
rs6797840	A	0.46	-0.016	0.002	-0.021	0.061
rs10145592	C	0.41	-0.015	0.002	-0.021	0.063
rs10947452	T	0.35	0.014	0.002	0.021	0.065
rs62332760	G	0.61	-0.014	0.002	-0.020	0.064
rs973734	C	0.15	0.017	0.003	-0.026	0.085
rs2045147	A	0.45	0.013	0.002	-0.019	0.062
rs112632642	G	0.93	-0.024	0.004	0.037	0.125
rs4382592	T	0.30	0.014	0.002	0.020	0.067
rs10772643	C	0.11	0.025	0.003	0.029	0.099
rs12475168	G	0.87	-0.018	0.003	-0.023	0.085
rs801733	A	0.64	0.017	0.002	0.017	0.064
rs10234444	G	0.82	0.016	0.003	0.021	0.084
rs11714337	G	0.57	0.014	0.002	-0.015	0.062
rs74996610	C	0.95	-0.031	0.005	0.035	0.163
rs6125906	C	0.91	-0.021	0.004	-0.024	0.112
rs2164744	T	0.64	-0.013	0.002	0.013	0.062
rs3137	G	0.85	0.017	0.003	-0.017	0.085
rs10771746	C	0.72	-0.014	0.002	0.014	0.069
rs2616830	G	0.46	0.016	0.002	-0.012	0.061
rs9834970	T	0.50	0.013	0.002	0.012	0.061
rs1022785	G	0.14	0.018	0.003	-0.017	0.088
rs2034768	A	0.49	0.015	0.002	-0.012	0.061
rs9964724	C	0.32	0.018	0.002	-0.013	0.066
rs12725114	G	0.80	0.015	0.003	0.014	0.075
rs62379379	G	0.93	-0.026	0.004	-0.022	0.122
rs6825241	C	0.54	-0.017	0.002	0.011	0.061
rs263771	C	0.77	-0.017	0.003	-0.011	0.073
rs11218575	C	0.57	0.015	0.002	0.010	0.062
rs57585211	T	0.83	-0.017	0.003	-0.013	0.083
rs11020045	A	0.67	-0.013	0.002	-0.010	0.065
rs4775373	T	0.36	0.013	0.002	0.008	0.063
rs2291681	G	0.58	0.013	0.002	-0.008	0.062

rs1243182	C	0.69	-0.019	0.002	-0.007	0.064
rs17789218	T	0.76	0.019	0.003	0.007	0.070
rs11896615	G	0.85	0.017	0.003	0.005	0.084
rs6850494	A	0.62	-0.014	0.002	0.003	0.063
rs8756	C	0.48	-0.013	0.002	0.002	0.060
rs11130793	C	0.60	0.013	0.002	-0.002	0.063
rs56858768	G	0.70	-0.015	0.002	0.001	0.068
**lobar ICH**						
rs10189857	A	0.57	-0.020	0.002	-0.241	0.067
rs11201422	T	0.67	0.013	0.002	-0.172	0.071
rs10041724	T	0.81	0.018	0.003	0.192	0.083
rs262890	A	0.70	-0.019	0.002	-0.162	0.075
rs11763734	A	0.51	-0.012	0.002	-0.146	0.068
rs10427502	G	0.62	0.014	0.002	-0.148	0.069
rs2460	G	0.74	-0.015	0.002	-0.151	0.077
rs6996198	C	0.84	-0.016	0.003	-0.173	0.088
rs55700114	G	0.71	-0.014	0.002	-0.134	0.072
rs11125515	C	0.73	-0.014	0.002	-0.134	0.075
rs6031440	A	0.40	0.012	0.002	-0.121	0.069
rs6973656	A	0.60	-0.013	0.002	-0.117	0.068
rs255292	C	0.40	-0.012	0.002	-0.116	0.069
rs9471333	C	0.45	0.013	0.002	0.113	0.067
rs11179838	C	0.64	0.013	0.002	-0.117	0.070
rs7184800	G	0.70	0.017	0.002	0.119	0.072
rs2034768	A	0.49	0.015	0.002	0.103	0.067
rs56398417	C	0.69	0.014	0.002	-0.114	0.074
rs12554512	T	0.58	0.021	0.002	-0.107	0.070
rs6141814	C	0.61	-0.014	0.002	0.105	0.070
rs17727474	C	0.83	0.018	0.003	-0.130	0.088
rs2291681	G	0.58	0.013	0.002	0.101	0.069
rs72781699	G	0.80	-0.019	0.003	0.123	0.084
rs12725114	G	0.80	0.015	0.003	-0.119	0.082
rs12272012	G	0.96	-0.030	0.005	0.261	0.182
rs2616830	G	0.46	0.016	0.002	0.091	0.065
rs11714337	G	0.57	0.014	0.002	-0.095	0.068
rs1913808	G	0.71	0.015	0.002	0.102	0.074
rs62379379	G	0.93	-0.026	0.004	-0.178	0.132
rs2694151	C	0.06	0.026	0.005	-0.191	0.144
rs4334769	G	0.47	0.012	0.002	-0.087	0.067
rs263771	C	0.77	-0.017	0.003	0.103	0.082
rs6124476	T	0.59	0.012	0.002	0.084	0.068
rs7700107	A	0.86	-0.021	0.003	0.117	0.095
rs328277	C	0.40	0.012	0.002	-0.083	0.069
rs9569734	A	0.84	0.019	0.003	0.115	0.097
rs66852340	C	0.78	-0.018	0.003	-0.096	0.082
rs10145592	C	0.41	-0.015	0.002	-0.080	0.069
rs3754970	T	0.50	-0.015	0.002	0.078	0.067

rs9834970	T	0.50	0.013	0.002	0.078	0.068
rs13029509	G	0.52	-0.018	0.002	0.076	0.068
rs12632778	T	0.95	-0.028	0.005	0.174	0.160
rs34864022	A	0.93	-0.026	0.004	-0.148	0.139
rs7788008	G	0.57	0.013	0.002	-0.069	0.067
rs10947452	T	0.35	0.014	0.002	0.070	0.072
rs1243182	C	0.69	-0.019	0.002	0.066	0.070
rs10772643	C	0.11	0.025	0.003	-0.103	0.110
rs3796386	G	0.57	-0.026	0.002	-0.061	0.068
rs10771746	C	0.72	-0.014	0.002	-0.066	0.074
rs984409	G	0.36	-0.015	0.002	0.061	0.069
rs71658797	T	0.88	-0.020	0.003	-0.096	0.111
rs7089973	C	0.62	-0.013	0.002	-0.061	0.072
rs9563168	G	0.79	0.018	0.003	0.070	0.082
rs114328297	T	0.78	0.014	0.003	0.073	0.086
rs78394231	T	0.90	-0.021	0.004	-0.097	0.115
rs12342024	G	0.89	-0.021	0.003	0.093	0.111
rs1022785	G	0.14	0.018	0.003	0.080	0.096
rs4775373	T	0.36	0.013	0.002	-0.059	0.071
rs11081851	G	0.44	-0.013	0.002	0.057	0.069
rs2971640	G	0.51	-0.013	0.002	-0.054	0.067
rs11689199	A	0.60	0.018	0.002	0.055	0.068
rs77215114	A	0.93	0.024	0.004	0.108	0.137
rs62332760	G	0.61	-0.014	0.002	-0.055	0.070
rs10739499	C	0.34	0.014	0.002	0.056	0.072
rs6905544	A	0.40	-0.019	0.002	-0.052	0.067
rs11896615	G	0.85	0.017	0.003	0.071	0.093
rs12475168	G	0.87	-0.018	0.003	0.071	0.096
rs56858768	G	0.70	-0.015	0.002	-0.055	0.075
rs74996610	C	0.95	-0.031	0.005	-0.120	0.176
rs35574015	T	0.71	-0.013	0.002	0.049	0.074
rs10246289	A	0.11	0.019	0.003	-0.067	0.105
rs7693082	G	0.30	0.015	0.002	0.044	0.071
rs11629120	T	0.59	-0.013	0.002	0.042	0.068
rs7564130	T	0.64	-0.015	0.002	-0.036	0.069
rs72725224	A	0.98	-0.046	0.008	-0.128	0.247
rs10057545	G	0.56	-0.014	0.002	0.034	0.067
rs10940659	A	0.53	-0.013	0.002	-0.034	0.067
rs1156541	C	0.22	0.014	0.003	0.039	0.079
rs8043253	C	0.57	-0.012	0.002	0.034	0.068
rs2791574	T	0.66	-0.013	0.002	-0.035	0.071
rs6850494	A	0.62	-0.014	0.002	0.032	0.068
rs12491503	G	0.67	-0.014	0.002	-0.033	0.071
rs2045147	A	0.45	0.013	0.002	-0.031	0.068
rs9528023	C	0.72	0.013	0.002	0.035	0.076
rs3137	G	0.85	0.017	0.003	0.042	0.094
rs17512836	T	0.97	0.042	0.007	0.085	0.195

rs11130793	C	0.60	0.013	0.002	0.029	0.070
rs4382592	T	0.30	0.014	0.002	-0.029	0.074
rs4845364	A	0.50	-0.015	0.002	-0.026	0.068
rs2336895	G	0.36	0.012	0.002	0.025	0.070
rs55909997	G	0.65	-0.014	0.002	-0.025	0.070
rs11810109	A	0.70	0.016	0.002	0.026	0.074
rs10890123	C	0.77	0.014	0.003	-0.028	0.082
rs4577309	A	0.47	0.016	0.002	0.023	0.068
rs11218575	C	0.57	0.015	0.002	-0.022	0.068
rs2173650	G	0.85	0.018	0.003	-0.031	0.096
rs9902312	T	0.68	0.015	0.002	0.023	0.071
rs11020045	A	0.67	-0.013	0.002	0.023	0.073
rs2447098	C	0.48	-0.015	0.002	0.021	0.069
rs10234444	G	0.82	0.016	0.003	-0.028	0.091
rs801733	A	0.64	0.017	0.002	0.021	0.071
rs10054327	G	0.58	0.017	0.002	-0.017	0.067
rs631130	T	0.37	-0.014	0.002	-0.020	0.076
rs11245482	T	0.61	-0.013	0.002	0.018	0.070
rs7609	A	0.23	0.015	0.003	-0.020	0.081
rs112632642	G	0.93	-0.024	0.004	0.032	0.137
rs17207890	G	0.66	0.016	0.002	0.015	0.070
rs17789218	T	0.76	0.019	0.003	0.015	0.079
rs2787374	T	0.41	0.012	0.002	0.013	0.069
rs374722	G	0.15	0.024	0.003	0.018	0.093
rs973734	C	0.15	0.017	0.003	0.017	0.095
rs57585211	T	0.83	-0.017	0.003	0.016	0.092
rs12289262	C	0.73	-0.014	0.002	-0.011	0.076
rs749671	G	0.63	0.016	0.002	-0.008	0.069
rs10737620	T	0.27	0.014	0.002	0.009	0.075
rs10791546	C	0.59	0.012	0.002	0.007	0.068
rs6131281	C	0.60	0.016	0.002	-0.007	0.070
rs12105701	C	0.40	-0.013	0.002	-0.007	0.069
rs6125906	C	0.91	-0.021	0.004	0.009	0.119
rs17379561	A	0.86	-0.025	0.003	-0.007	0.098
rs9964724	C	0.32	0.018	0.002	-0.005	0.073
rs8756	C	0.48	-0.013	0.002	0.005	0.066
rs1031423	T	0.22	-0.019	0.003	-0.004	0.081
rs6825241	C	0.54	-0.017	0.002	0.003	0.067
rs12476388	C	0.71	0.013	0.002	0.003	0.077
rs6797840	A	0.46	-0.016	0.002	-0.003	0.067
rs1352890	C	0.48	-0.012	0.002	-0.002	0.068
rs2164744	T	0.64	-0.013	0.002	0.002	0.069
**WMH**						
rs11917871	T	0.42	-0.017	0.002	0.037	0.013
rs4973576	C	0.30	-0.015	0.002	0.039	0.014
rs17512836	T	0.97	0.042	0.007	-0.103	0.039
rs6905544	A	0.40	-0.019	0.002	-0.031	0.013

rs6141814	C	0.61	-0.014	0.002	0.029	0.013
rs1243182	C	0.69	-0.019	0.002	0.030	0.013
rs2173650	G	0.85	0.018	0.003	0.039	0.018
rs7700107	A	0.86	-0.021	0.003	-0.038	0.018
rs10737620	T	0.27	0.014	0.002	0.028	0.014
rs6124476	T	0.59	0.012	0.002	0.025	0.013
rs11689199	A	0.60	0.018	0.002	0.025	0.013
rs12491503	G	0.67	-0.014	0.002	-0.026	0.013
rs6131281	C	0.60	0.016	0.002	-0.024	0.013
rs74996610	C	0.95	-0.031	0.005	-0.057	0.031
rs34864022	A	0.93	-0.026	0.004	0.045	0.026
rs2971640	G	0.51	-0.013	0.002	0.022	0.012
rs3754970	T	0.50	-0.015	0.002	-0.022	0.012
rs11896615	G	0.85	0.017	0.003	0.031	0.018
rs2164744	T	0.64	-0.013	0.002	0.022	0.013
rs3137	G	0.85	0.017	0.003	0.028	0.017
rs17789218	T	0.76	0.019	0.003	-0.024	0.014
rs4523073	A	0.59	-0.014	0.002	0.020	0.013
rs10427502	G	0.62	0.014	0.002	0.021	0.013
rs3796386	G	0.57	-0.026	0.002	-0.020	0.013
rs255292	C	0.40	-0.012	0.002	-0.020	0.013
rs7693703	G	0.91	0.023	0.004	-0.033	0.022
rs7184800	G	0.70	0.017	0.002	0.020	0.013
rs1022785	G	0.14	0.018	0.003	0.027	0.018
rs11629120	T	0.59	-0.013	0.002	0.019	0.013
rs6125906	C	0.91	-0.021	0.004	-0.031	0.021
rs11130793	C	0.60	0.013	0.002	0.019	0.013
rs10994943	T	0.58	0.013	0.002	0.018	0.013
rs74802478	G	0.82	0.017	0.003	0.023	0.016
rs4937842	G	0.63	-0.013	0.002	-0.019	0.013
rs9834970	T	0.50	0.013	0.002	0.017	0.012
rs12632778	T	0.95	-0.028	0.005	0.040	0.029
rs9563168	G	0.79	0.018	0.003	0.020	0.015
rs648044	A	0.40	-0.014	0.002	-0.017	0.013
rs77215114	A	0.93	0.024	0.004	-0.033	0.025
rs4845364	A	0.50	-0.015	0.002	-0.017	0.012
rs56858768	G	0.70	-0.015	0.002	0.018	0.014
rs11081851	G	0.44	-0.013	0.002	-0.016	0.012
rs10145592	C	0.41	-0.015	0.002	-0.016	0.013
rs7609	A	0.23	0.015	0.003	-0.019	0.015
rs4382592	T	0.30	0.014	0.002	0.017	0.014
rs11714337	G	0.57	0.014	0.002	-0.015	0.012
rs11179838	C	0.64	0.013	0.002	0.015	0.013
rs9528023	C	0.72	0.013	0.002	0.016	0.014
rs42210	G	0.29	-0.014	0.002	0.016	0.014
rs6797840	A	0.46	-0.016	0.002	0.014	0.013
rs1913808	G	0.71	0.015	0.002	-0.015	0.014

rs262890	A	0.70	-0.019	0.002	0.015	0.014
rs55700114	G	0.71	-0.014	0.002	0.015	0.014
rs2336895	G	0.36	0.012	0.002	-0.014	0.013
rs12541615	T	0.82	-0.018	0.003	-0.016	0.016
rs9902312	T	0.68	0.015	0.002	0.014	0.013
rs7693082	G	0.30	0.015	0.002	0.014	0.014
rs72671494	T	0.86	-0.017	0.003	0.018	0.018
rs72828890	C	0.87	0.019	0.003	-0.019	0.019
rs34811474	G	0.77	0.015	0.003	-0.014	0.015
rs4675246	G	0.80	-0.015	0.003	-0.015	0.015
rs10772643	C	0.11	0.025	0.003	0.019	0.020
rs6850494	A	0.62	-0.014	0.002	-0.012	0.013
rs10876864	G	0.43	-0.013	0.002	-0.012	0.013
rs2717559	A	0.56	0.012	0.002	0.012	0.013
rs11245482	T	0.61	-0.013	0.002	0.012	0.013
rs10041724	T	0.81	0.018	0.003	0.014	0.016
rs303753	G	0.65	-0.014	0.002	-0.012	0.013
rs59210467	C	0.69	-0.013	0.002	-0.012	0.014
rs984409	G	0.36	-0.015	0.002	-0.011	0.013
rs9867121	C	0.82	0.019	0.003	-0.014	0.016
rs13107325	C	0.93	-0.029	0.004	-0.020	0.024
rs7157001	A	0.74	-0.014	0.002	0.012	0.014
rs6472942	T	0.57	-0.013	0.002	0.010	0.013
rs1421334	A	0.45	0.017	0.002	0.010	0.013
rs10189857	A	0.57	-0.020	0.002	-0.010	0.013
rs10739499	C	0.34	0.014	0.002	-0.010	0.013
rs12272012	G	0.96	-0.030	0.005	-0.023	0.029
rs1352890	C	0.48	-0.012	0.002	0.010	0.012
rs10932837	C	0.49	-0.013	0.002	-0.009	0.012
rs10246289	A	0.11	0.019	0.003	0.015	0.020
rs17727474	C	0.83	0.018	0.003	-0.012	0.017
rs11125515	C	0.73	-0.014	0.002	-0.010	0.014
rs17379561	A	0.86	-0.025	0.003	-0.012	0.018
rs2447098	C	0.48	-0.015	0.002	0.009	0.013
rs62379379	G	0.93	-0.026	0.004	-0.016	0.024
rs138256022	C	0.96	-0.031	0.006	-0.022	0.032
rs62332760	G	0.61	-0.014	0.002	0.008	0.013
rs11218575	C	0.57	0.015	0.002	-0.008	0.013
rs11654952	T	0.86	-0.017	0.003	0.011	0.018
rs801733	A	0.64	0.017	0.002	0.008	0.013
rs11657730	C	0.64	0.013	0.002	0.008	0.013
rs62641636	A	0.69	0.014	0.002	0.008	0.013
rs10771746	C	0.72	-0.014	0.002	0.008	0.014
rs2787374	T	0.41	0.012	0.002	-0.007	0.013
rs114328297	T	0.78	0.014	0.003	0.009	0.015
rs112632642	G	0.93	-0.024	0.004	-0.014	0.026
rs7248205	C	0.40	0.014	0.002	0.007	0.013

rs183566425	A	0.98	0.046	0.008	0.027	0.050
rs78394231	T	0.90	-0.021	0.004	0.011	0.021
rs6031440	A	0.40	0.012	0.002	0.007	0.013
rs631130	T	0.37	-0.014	0.002	-0.007	0.014
rs263771	C	0.77	-0.017	0.003	-0.008	0.015
rs10890123	C	0.77	0.014	0.003	0.008	0.015
rs328277	C	0.40	0.012	0.002	-0.007	0.013
rs114600294	G	0.79	-0.016	0.003	0.008	0.016
rs13029509	G	0.52	-0.018	0.002	-0.006	0.012
rs749671	G	0.63	0.016	0.002	0.006	0.013
rs4334769	G	0.47	0.012	0.002	-0.005	0.012
rs9718104	T	0.94	-0.041	0.005	0.012	0.027
rs12289262	C	0.73	-0.014	0.002	-0.006	0.014
rs148544378	C	0.98	0.053	0.008	-0.020	0.046
rs10947452	T	0.35	0.014	0.002	-0.005	0.013
rs1156541	C	0.22	0.014	0.003	0.006	0.015
rs11201422	T	0.67	0.013	0.002	0.005	0.013
rs6973656	A	0.60	-0.013	0.002	0.005	0.013
rs374722	G	0.15	0.024	0.003	0.007	0.018
rs6996198	C	0.84	-0.016	0.003	-0.006	0.017
rs1451533	G	0.72	-0.016	0.002	0.005	0.014
rs1278847	C	0.69	0.016	0.002	-0.005	0.014
rs72834698	G	0.86	0.023	0.003	0.006	0.018
rs35574015	T	0.71	-0.013	0.002	0.005	0.014
rs17207890	G	0.66	0.016	0.002	0.004	0.013
rs2045147	A	0.45	0.013	0.002	0.004	0.013
rs7189927	T	0.36	0.015	0.002	0.004	0.013
rs10054327	G	0.58	0.017	0.002	0.004	0.013
rs10940659	A	0.53	-0.013	0.002	-0.004	0.013
rs12554512	T	0.58	0.021	0.002	-0.004	0.013
rs7089973	C	0.62	-0.013	0.002	-0.004	0.013
rs71658797	T	0.88	-0.020	0.003	-0.006	0.019
rs6825241	C	0.54	-0.017	0.002	0.004	0.012
rs6673341	T	0.47	-0.015	0.002	0.003	0.012
rs2616830	G	0.46	0.016	0.002	-0.003	0.012
rs12105701	C	0.40	-0.013	0.002	0.003	0.013
rs11810109	A	0.70	0.016	0.002	-0.003	0.014
rs56103247	C	0.94	0.030	0.005	0.006	0.027
rs7564130	T	0.64	-0.015	0.002	0.003	0.013
rs1031423	T	0.22	-0.019	0.003	-0.003	0.015
rs7788008	G	0.57	0.013	0.002	-0.003	0.012
rs72781699	G	0.80	-0.019	0.003	-0.003	0.015
rs10791546	C	0.59	0.012	0.002	0.002	0.013
rs57585211	T	0.83	-0.017	0.003	-0.003	0.016
rs8043253	C	0.57	-0.012	0.002	0.002	0.012
rs1790135	C	0.24	-0.014	0.003	-0.002	0.014
rs11763734	A	0.51	-0.012	0.002	-0.002	0.012

rs56398417	C	0.69	0.014	0.002	0.002	0.013
rs2034768	A	0.49	0.015	0.002	-0.002	0.012
rs72725224	A	0.98	-0.046	0.008	0.007	0.047
rs12725114	G	0.80	0.015	0.003	0.002	0.016
rs4577309	A	0.47	0.016	0.002	0.002	0.012
rs10234444	G	0.82	0.016	0.003	0.002	0.016
rs12342024	G	0.89	-0.021	0.003	0.002	0.019
rs9964724	C	0.32	0.018	0.002	0.002	0.013
rs10057545	G	0.56	-0.014	0.002	-0.001	0.012
rs4775373	T	0.36	0.013	0.002	-0.001	0.013
rs8756	C	0.48	-0.013	0.002	-0.001	0.012
rs9471333	C	0.45	0.013	0.002	-0.001	0.013
rs2694151	C	0.06	0.026	0.005	0.002	0.027
rs2460	G	0.74	-0.015	0.002	0.001	0.014
rs2291681	G	0.58	0.013	0.002	-0.001	0.013
rs141425667	G	0.93	-0.024	0.004	-0.002	0.025
rs2073869	C	0.83	0.019	0.003	0.001	0.016
rs973734	C	0.15	0.017	0.003	0.001	0.017
rs2791574	T	0.66	-0.013	0.002	0.000	0.013
**AD**						
rs10041724	T	0.81	0.018	0.003	-0.005	0.018
rs10054327	G	0.58	0.017	0.002	0.024	0.014
rs10145592	C	0.41	-0.015	0.002	-0.008	0.015
rs10189857	A	0.57	-0.020	0.002	-0.017	0.015
rs1022785	G	0.14	0.018	0.003	0.025	0.020
rs10234444	G	0.82	0.016	0.003	-0.018	0.019
rs10246289	A	0.11	0.019	0.003	-0.007	0.022
rs1031423	T	0.22	-0.019	0.003	-0.008	0.018
rs10427502	G	0.62	0.014	0.002	0.005	0.015
rs10737620	T	0.27	0.014	0.002	0.004	0.016
rs10771746	C	0.72	-0.014	0.002	0.006	0.016
rs10772643	C	0.11	0.025	0.003	-0.008	0.024
rs10876864	G	0.43	-0.013	0.002	0.002	0.014
rs10890123	C	0.77	0.014	0.003	0.028	0.018
rs10932837	C	0.49	-0.013	0.002	-0.019	0.014
rs10940659	A	0.53	-0.013	0.002	0.010	0.014
rs10994943	T	0.58	0.013	0.002	0.005	0.015
rs11020045	A	0.67	-0.013	0.002	0.007	0.015
rs11130793	C	0.60	0.013	0.002	0.011	0.015
rs11201422	T	0.67	0.013	0.002	-0.005	0.015
rs11218575	C	0.57	0.015	0.002	-0.009	0.015
rs11245482	T	0.61	-0.013	0.002	0.002	0.015
rs114328297	T	0.78	0.014	0.003	0.022	0.018
rs114600294	G	0.79	-0.016	0.003	-0.010	0.024
rs1156541	C	0.22	0.014	0.003	-0.030	0.017
rs11654952	T	0.86	-0.017	0.003	-0.064	0.023
rs11657730	C	0.64	0.013	0.002	0.016	0.018

rs11689199	A	0.60	0.018	0.002	0.027	0.015
rs11714337	G	0.57	0.014	0.002	0.008	0.014
rs11763734	A	0.51	-0.012	0.002	0.008	0.014
rs11810109	A	0.70	0.016	0.002	-0.003	0.015
rs12105701	C	0.40	-0.013	0.002	0.014	0.015
rs12272012	G	0.96	-0.030	0.005	0.014	0.036
rs12289262	C	0.73	-0.014	0.002	-0.032	0.016
rs1243182	C	0.69	-0.019	0.002	0.023	0.015
rs12476388	C	0.71	0.013	0.002	-0.010	0.016
rs12491503	G	0.67	-0.014	0.002	0.011	0.015
rs12541615	T	0.82	-0.018	0.003	0.040	0.019
rs12554512	T	0.58	0.021	0.002	0.030	0.016
rs12725114	G	0.80	0.015	0.003	-0.039	0.018
rs1278847	C	0.69	0.016	0.002	0.010	0.016
rs13029509	G	0.52	-0.018	0.002	-0.001	0.014
rs13107325	C	0.93	-0.029	0.004	-0.020	0.027
rs138256022	C	0.96	-0.031	0.006	0.007	0.040
rs141184308	A	0.98	0.043	0.008	0.092	0.080
rs1421334	A	0.45	0.017	0.002	-0.012	0.015
rs1451533	G	0.72	-0.016	0.002	-0.023	0.017
rs17207890	G	0.66	0.016	0.002	0.028	0.015
rs17379561	A	0.86	-0.025	0.003	-0.012	0.021
rs17512836	T	0.97	0.042	0.007	-0.003	0.042
rs17727474	C	0.83	0.018	0.003	0.003	0.019
rs17789218	T	0.76	0.019	0.003	0.007	0.018
rs2034768	A	0.49	0.015	0.002	-0.002	0.014
rs2045147	A	0.45	0.013	0.002	0.015	0.014
rs2073869	C	0.83	0.019	0.003	0.006	0.019
rs2092829	G	0.71	0.014	0.002	0.000	0.016
rs2164744	T	0.64	-0.013	0.002	-0.004	0.015
rs2173650	G	0.85	0.018	0.003	-0.019	0.020
rs2184364	A	0.78	0.016	0.003	0.026	0.018
rs2447098	C	0.48	-0.015	0.002	0.016	0.015
rs2460	G	0.74	-0.015	0.002	-0.003	0.017
rs2584597	T	0.66	0.015	0.002	-0.016	0.016
rs2616830	G	0.46	0.016	0.002	0.016	0.015
rs262890	A	0.70	-0.019	0.002	-0.003	0.016
rs2717559	A	0.56	0.012	0.002	-0.006	0.015
rs2787374	T	0.41	0.012	0.002	-0.029	0.014
rs303753	G	0.65	-0.014	0.002	0.002	0.015
rs34864022	A	0.93	-0.026	0.004	-0.066	0.032
rs35574015	T	0.71	-0.013	0.002	-0.007	0.016
rs374722	G	0.15	0.024	0.003	0.019	0.020
rs3754970	T	0.50	-0.015	0.002	0.013	0.014
rs3796386	G	0.57	-0.026	0.002	0.004	0.015
rs405797	T	0.25	-0.015	0.003	-0.001	0.016
rs42210	G	0.29	-0.014	0.002	-0.007	0.016

rs4334769	G	0.47	0.012	0.002	-0.002	0.014
rs4382592	T	0.30	0.014	0.002	-0.001	0.015
rs4523073	A	0.59	-0.014	0.002	0.003	0.015
rs4577309	A	0.47	0.016	0.002	0.014	0.014
rs4675246	G	0.80	-0.015	0.003	0.006	0.018
rs4775373	T	0.36	0.013	0.002	-0.016	0.015
rs4845364	A	0.50	-0.015	0.002	-0.002	0.014
rs4937842	G	0.63	-0.013	0.002	0.014	0.015
rs4973576	C	0.30	-0.015	0.002	-0.027	0.016
rs55700114	G	0.71	-0.014	0.002	0.018	0.015
rs55909997	G	0.65	-0.014	0.002	0.008	0.015
rs56103247	C	0.94	0.030	0.005	-0.037	0.037
rs56858768	G	0.70	-0.015	0.002	0.001	0.016
rs57585211	T	0.83	-0.017	0.003	-0.012	0.019
rs6131281	C	0.60	0.016	0.002	-0.005	0.015
rs6141814	C	0.61	-0.014	0.002	-0.002	0.015
rs62379379	G	0.93	-0.026	0.004	-0.011	0.029
rs62641636	A	0.69	0.014	0.002	0.025	0.016
rs6472942	T	0.57	-0.013	0.002	-0.008	0.015
rs6673341	T	0.47	-0.015	0.002	-0.003	0.014
rs66852340	C	0.78	-0.018	0.003	-0.027	0.018
rs6721975	T	0.23	-0.017	0.003	-0.017	0.018
rs6797840	A	0.46	-0.016	0.002	0.020	0.014
rs6825241	C	0.54	-0.017	0.002	0.017	0.015
rs6850494	A	0.62	-0.014	0.002	0.003	0.015
rs6905544	A	0.40	-0.019	0.002	-0.029	0.015
rs6973656	A	0.60	-0.013	0.002	-0.018	0.015
rs6996198	C	0.84	-0.016	0.003	-0.004	0.020
rs7089973	C	0.62	-0.013	0.002	0.034	0.015
rs7157001	A	0.74	-0.014	0.002	-0.007	0.020
rs71658797	T	0.88	-0.020	0.003	0.019	0.024
rs7184800	G	0.70	0.017	0.002	0.002	0.015
rs7189927	T	0.36	0.015	0.002	-0.024	0.016
rs7248205	C	0.40	0.014	0.002	-0.032	0.015
rs72671494	T	0.86	-0.017	0.003	-0.041	0.024
rs72781699	G	0.80	-0.019	0.003	-0.020	0.018
rs72828890	C	0.87	0.019	0.003	0.012	0.025
rs72834698	G	0.86	0.023	0.003	0.029	0.022
rs749671	G	0.63	0.016	0.002	-0.021	0.015
rs7564130	T	0.64	-0.015	0.002	-0.018	0.015
rs7693082	G	0.30	0.015	0.002	0.016	0.016
rs7693703	G	0.91	0.023	0.004	0.007	0.025
rs7700107	A	0.86	-0.021	0.003	-0.038	0.020
rs7716447	A	0.64	-0.013	0.002	0.028	0.020
rs77215114	A	0.93	0.024	0.004	0.014	0.032
rs7991062	C	0.66	-0.018	0.002	0.012	0.015
rs801733	A	0.64	0.017	0.002	-0.023	0.015

rs8043253	C	0.57	-0.012	0.002	0.014	0.015
rs8756	C	0.48	-0.013	0.002	-0.011	0.014
rs9471333	C	0.45	0.013	0.002	0.025	0.014
rs9563168	G	0.79	0.018	0.003	-0.014	0.017
rs9569734	A	0.84	0.019	0.003	0.013	0.020
rs9718104	T	0.94	-0.041	0.005	0.020	0.035
rs973734	C	0.15	0.017	0.003	0.028	0.020
rs9834970	T	0.50	0.013	0.002	0.002	0.014
rs984409	G	0.36	-0.015	0.002	0.028	0.015
rs9867121	C	0.82	0.019	0.003	-0.019	0.019
rs9902312	T	0.68	0.015	0.002	-0.008	0.015
rs9964724	C	0.32	0.018	0.002	-0.005	0.016
**MS**						
rs10041724	T	0.81	0.018	0.003	0.008	0.015
rs10145592	C	0.41	-0.015	0.002	0.016	0.039
rs10189857	A	0.57	-0.020	0.002	-0.007	0.014
rs1022785	G	0.14	0.018	0.003	-0.004	0.003
rs10234444	G	0.82	0.016	0.003	0.021	0.052
rs10246289	A	0.11	0.019	0.003	-0.017	1.966
rs1031423	T	0.22	-0.019	0.003	0.032	0.027
rs10427502	G	0.62	0.014	0.002	0.007	0.014
rs10737620	T	0.27	0.014	0.002	0.009	0.035
rs10771746	C	0.72	-0.014	0.002	-0.003	0.003
rs10772643	C	0.11	0.025	0.003	-0.028	0.048
rs10876864	G	0.43	-0.013	0.002	-0.011	0.405
rs10890123	C	0.77	0.014	0.003	-0.026	0.030
rs10932837	C	0.49	-0.013	0.002	0.009	0.052
rs10940659	A	0.53	-0.013	0.002	0.013	0.069
rs10994943	T	0.58	0.013	0.002	0.018	0.030
rs11020045	A	0.67	-0.013	0.002	-0.010	0.080
rs11130793	C	0.60	0.013	0.002	-0.005	0.008
rs11201422	T	0.67	0.013	0.002	0.005	0.006
rs11218575	C	0.57	0.015	0.002	-0.002	0.002
rs11245482	T	0.61	-0.013	0.002	-0.015	0.044
rs1156541	C	0.22	0.014	0.003	0.008	0.018
rs11654952	T	0.86	-0.017	0.003	0.062	0.031
rs11657730	C	0.64	0.013	0.002	-0.013	0.026
rs11689199	A	0.60	0.018	0.002	-0.016	0.039
rs11714337	G	0.57	0.014	0.002	0.076	0.017
rs11763734	A	0.51	-0.012	0.002	0.012	0.105
rs11810109	A	0.70	0.016	0.002	0.015	0.051
rs12105701	C	0.40	-0.013	0.002	0.001	0.001
rs12272012	G	0.96	-0.030	0.005	-0.063	0.061
rs12289262	C	0.73	-0.014	0.002	-0.030	0.024
rs1243182	C	0.69	-0.019	0.002	-0.040	0.020
rs12476388	C	0.71	0.013	0.002	0.019	0.042
rs12491503	G	0.67	-0.014	0.002	0.023	0.026

rs12541615	T	0.82	-0.018	0.003	0.011	0.032
rs12554512	T	0.58	0.021	0.002	-0.007	0.014
rs12725114	G	0.80	0.015	0.003	0.008	0.015
rs13029509	G	0.52	-0.018	0.002	-0.008	0.023
rs13107325	C	0.93	-0.029	0.004	-0.114	0.041
rs1421334	A	0.45	0.017	0.002	0.002	0.001
rs1451533	G	0.72	-0.016	0.002	-0.002	0.002
rs17207890	G	0.66	0.016	0.002	0.004	0.004
rs17379561	A	0.86	-0.025	0.003	0.024	0.046
rs17512836	T	0.97	0.042	0.007	-0.021	0.051
rs17727474	C	0.83	0.018	0.003	0.015	0.571
rs17789218	T	0.76	0.019	0.003	-0.033	0.025
rs2034768	A	0.49	0.015	0.002	0.011	0.224
rs2045147	A	0.45	0.013	0.002	-0.042	0.018
rs2073869	C	0.83	0.019	0.003	-0.004	0.004
rs2092829	G	0.71	0.014	0.002	0.060	0.020
rs2164744	T	0.64	-0.013	0.002	-0.004	0.005
rs2173650	G	0.85	0.018	0.003	0.004	0.004
rs2184364	A	0.78	0.016	0.003	-0.016	0.092
rs2447098	C	0.48	-0.015	0.002	0.071	0.018
rs2460	G	0.74	-0.015	0.002	0.016	0.065
rs2584597	T	0.66	0.015	0.002	-0.037	0.023
rs2616830	G	0.46	0.016	0.002	0.000	0.000
rs262890	A	0.70	-0.019	0.002	-0.011	0.142
rs2717559	A	0.56	0.012	0.002	0.018	0.039
rs2787374	T	0.41	0.012	0.002	0.003	0.002
rs303753	G	0.65	-0.014	0.002	-0.008	0.024
rs34864022	A	0.93	-0.026	0.004	-0.032	0.082
rs35574015	T	0.71	-0.013	0.002	0.028	0.025
rs374722	G	0.15	0.024	0.003	0.048	0.026
rs3754970	T	0.50	-0.015	0.002	0.008	0.030
rs3796386	G	0.57	-0.026	0.002	-0.007	0.014
rs42210	G	0.29	-0.014	0.002	0.035	0.023
rs4334769	G	0.47	0.012	0.002	-0.018	0.029
rs4382592	T	0.30	0.014	0.002	0.039	0.021
rs4523073	A	0.59	-0.014	0.002	-0.017	0.033
rs4577309	A	0.47	0.016	0.002	0.035	0.019
rs4675246	G	0.80	-0.015	0.003	-0.015	0.221
rs4775373	T	0.36	0.013	0.002	-0.007	0.015
rs4845364	A	0.50	-0.015	0.002	0.001	0.000
rs4937842	G	0.63	-0.013	0.002	0.002	0.002
rs4973576	C	0.30	-0.015	0.002	0.017	0.045
rs55700114	G	0.71	-0.014	0.002	0.005	0.008
rs55909997	G	0.65	-0.014	0.002	-0.025	0.024
rs56103247	C	0.94	0.030	0.005	0.034	0.436
rs56858768	G	0.70	-0.015	0.002	-0.030	0.023
rs57585211	T	0.83	-0.017	0.003	0.032	0.030

rs6131281	C	0.60	0.016	0.002	0.001	0.001
rs6141814	C	0.61	-0.014	0.002	-0.022	0.025
rs62379379	G	0.93	-0.026	0.004	-0.014	0.030
rs62641636	A	0.69	0.014	0.002	-0.066	0.019
rs6472942	T	0.57	-0.013	0.002	-0.020	0.026
rs6673341	T	0.47	-0.015	0.002	0.019	0.028
rs66852340	C	0.78	-0.018	0.003	0.012	0.089
rs6721975	T	0.23	-0.017	0.003	-0.018	0.352
rs6797840	A	0.46	-0.016	0.002	0.015	0.047
rs6825241	C	0.54	-0.017	0.002	-0.019	0.027
rs6850494	A	0.62	-0.014	0.002	-0.002	0.002
rs6905544	A	0.40	-0.019	0.002	-0.027	0.021
rs6973656	A	0.60	-0.013	0.002	-0.023	0.024
rs6996198	C	0.84	-0.016	0.003	0.008	0.013
rs7089973	C	0.62	-0.013	0.002	-0.030	0.022
rs7157001	A	0.74	-0.014	0.002	0.022	0.156
rs71658797	T	0.88	-0.020	0.003	-0.090	0.029
rs7184800	G	0.70	0.017	0.002	-0.002	0.001
rs7189927	T	0.36	0.015	0.002	0.003	0.003
rs7248205	C	0.40	0.014	0.002	-0.051	0.020
rs72671494	T	0.86	-0.017	0.003	-0.045	0.063
rs72781699	G	0.80	-0.019	0.003	-0.021	0.040
rs72828890	C	0.87	0.019	0.003	-0.017	0.159
rs72834698	G	0.86	0.023	0.003	-0.006	0.006
rs749671	G	0.63	0.016	0.002	-0.047	0.018
rs7564130	T	0.64	-0.015	0.002	0.006	0.009
rs7693082	G	0.30	0.015	0.002	-0.014	0.095
rs7693703	G	0.91	0.023	0.004	0.012	0.024
rs7700107	A	0.86	-0.021	0.003	0.003	0.002
rs7716447	A	0.64	-0.013	0.002	0.045	0.021
rs7991062	C	0.66	-0.018	0.002	0.020	0.031
rs801733	A	0.64	0.017	0.002	-0.038	0.019
rs8043253	C	0.57	-0.012	0.002	-0.007	0.014
rs8756	C	0.48	-0.013	0.002	0.006	0.010
rs9471333	C	0.45	0.013	0.002	-0.018	0.030
rs9563168	G	0.79	0.018	0.003	0.016	0.072
rs9569734	A	0.84	0.019	0.003	-0.034	0.030
rs9718104	T	0.94	-0.041	0.005	0.046	0.160
rs973734	C	0.15	0.017	0.003	0.002	0.001
rs9834970	T	0.50	0.013	0.002	-0.013	0.079
rs984409	G	0.36	-0.015	0.002	-0.011	0.137
rs9867121	C	0.82	0.019	0.003	0.002	0.001
rs9902312	T	0.68	0.015	0.002	0.003	0.003
rs9964724	C	0.32	0.018	0.002	-0.005	0.006
**SBP**						
rs10041724	T	0.81	0.018	0.003	0.046	0.038
rs10054327	G	0.58	0.017	0.002	-0.174	0.031

rs10145592	C	0.41	-0.015	0.002	-0.012	0.031
rs10189857	A	0.57	-0.020	0.002	0.024	0.031
rs1022785	G	0.14	0.018	0.003	-0.026	0.043
rs10234444	G	0.82	0.016	0.003	-0.039	0.041
rs10246289	A	0.11	0.019	0.003	0.022	0.047
rs1031423	T	0.22	-0.019	0.003	0.000	0.037
rs10427502	G	0.62	0.014	0.002	0.005	0.031
rs10737620	T	0.27	0.014	0.002	0.160	0.034
rs10771746	C	0.72	-0.014	0.002	0.031	0.033
rs10772643	C	0.11	0.025	0.003	0.220	0.048
rs10876864	G	0.43	-0.013	0.002	-0.117	0.030
rs10890123	C	0.77	0.014	0.003	0.058	0.036
rs10932837	C	0.49	-0.013	0.002	-0.088	0.030
rs10940659	A	0.53	-0.013	0.002	0.036	0.031
rs10994943	T	0.58	0.013	0.002	0.009	0.031
rs11130793	C	0.60	0.013	0.002	-0.023	0.031
rs11201422	T	0.67	0.013	0.002	0.048	0.032
rs11218575	C	0.57	0.015	0.002	-0.075	0.031
rs11245482	T	0.61	-0.013	0.002	-0.097	0.031
rs114328297	T	0.78	0.014	0.003	-0.042	0.037
rs1156541	C	0.22	0.014	0.003	-0.014	0.036
rs11654952	T	0.86	-0.017	0.003	-0.088	0.045
rs11657730	C	0.64	0.013	0.002	0.194	0.033
rs11689199	A	0.60	0.018	0.002	-0.070	0.031
rs11714337	G	0.57	0.014	0.002	0.065	0.031
rs11763734	A	0.51	-0.012	0.002	-0.060	0.030
rs11810109	A	0.70	0.016	0.002	-0.065	0.033
rs12105701	C	0.40	-0.013	0.002	0.014	0.031
rs12272012	G	0.96	-0.030	0.005	-0.155	0.075
rs12289262	C	0.73	-0.014	0.002	0.054	0.034
rs1243182	C	0.69	-0.019	0.002	-0.067	0.033
rs12491503	G	0.67	-0.014	0.002	-0.044	0.032
rs12541615	T	0.82	-0.018	0.003	-0.094	0.039
rs12554512	T	0.58	0.021	0.002	0.003	0.031
rs12725114	G	0.80	0.015	0.003	0.084	0.037
rs1278847	C	0.69	0.016	0.002	-0.060	0.033
rs13029509	G	0.52	-0.018	0.002	-0.107	0.030
rs13107325	C	0.93	-0.029	0.004	0.909	0.059
rs138256022	C	0.96	-0.031	0.006	-0.181	0.084
rs1421334	A	0.45	0.017	0.002	0.077	0.031
rs1451533	G	0.72	-0.016	0.002	-0.022	0.034
rs17207890	G	0.66	0.016	0.002	0.043	0.032
rs17379561	A	0.86	-0.025	0.003	0.070	0.043
rs17512836	T	0.97	0.042	0.007	-0.180	0.093
rs17727474	C	0.83	0.018	0.003	-0.069	0.041
rs17789218	T	0.76	0.019	0.003	0.117	0.036
rs2034768	A	0.49	0.015	0.002	-0.013	0.030

rs2045147	A	0.45	0.013	0.002	0.008	0.030
rs2073869	C	0.83	0.019	0.003	-0.101	0.040
rs2164744	T	0.64	-0.013	0.002	0.073	0.031
rs2173650	G	0.85	0.018	0.003	-0.046	0.043
rs2447098	C	0.48	-0.015	0.002	-0.056	0.030
rs2460	G	0.74	-0.015	0.002	-0.109	0.034
rs2616830	G	0.46	0.016	0.002	0.017	0.030
rs262890	A	0.70	-0.019	0.002	-0.016	0.033
rs2717559	A	0.56	0.012	0.002	-0.038	0.032
rs2787374	T	0.41	0.012	0.002	-0.041	0.030
rs303753	G	0.65	-0.014	0.002	-0.080	0.032
rs34864022	A	0.93	-0.026	0.004	0.089	0.062
rs35574015	T	0.71	-0.013	0.002	-0.070	0.033
rs374722	G	0.15	0.024	0.003	0.052	0.042
rs3754970	T	0.50	-0.015	0.002	-0.080	0.030
rs3796386	G	0.57	-0.026	0.002	-0.062	0.031
rs42210	G	0.29	-0.014	0.002	0.019	0.034
rs4334769	G	0.47	0.012	0.002	-0.057	0.030
rs4382592	T	0.30	0.014	0.002	-0.035	0.033
rs4523073	A	0.59	-0.014	0.002	0.060	0.031
rs4577309	A	0.47	0.016	0.002	0.167	0.030
rs4675246	G	0.80	-0.015	0.003	0.038	0.038
rs4775373	T	0.36	0.013	0.002	0.073	0.031
rs4845364	A	0.50	-0.015	0.002	0.035	0.030
rs4937842	G	0.63	-0.013	0.002	0.050	0.031
rs4973576	C	0.30	-0.015	0.002	0.083	0.033
rs55700114	G	0.71	-0.014	0.002	-0.002	0.033
rs56103247	C	0.94	0.030	0.005	-0.065	0.072
rs56858768	G	0.70	-0.015	0.002	-0.018	0.033
rs57585211	T	0.83	-0.017	0.003	-0.021	0.040
rs6131281	C	0.60	0.016	0.002	0.136	0.031
rs6141814	C	0.61	-0.014	0.002	-0.136	0.031
rs62379379	G	0.93	-0.026	0.004	-0.154	0.059
rs62641636	A	0.69	0.014	0.002	0.001	0.033
rs6472942	T	0.57	-0.013	0.002	0.251	0.031
rs6673341	T	0.47	-0.015	0.002	-0.121	0.031
rs6797840	A	0.46	-0.016	0.002	0.032	0.030
rs6825241	C	0.54	-0.017	0.002	-0.109	0.030
rs6850494	A	0.62	-0.014	0.002	0.001	0.031
rs6905544	A	0.40	-0.019	0.002	-0.122	0.031
rs6973656	A	0.60	-0.013	0.002	0.126	0.031
rs6996198	C	0.84	-0.016	0.003	-0.163	0.041
rs7089973	C	0.62	-0.013	0.002	-0.111	0.031
rs7157001	A	0.74	-0.014	0.002	-0.021	0.037
rs71658797	T	0.88	-0.020	0.003	0.206	0.047
rs7184800	G	0.70	0.017	0.002	0.111	0.033
rs7189927	T	0.36	0.015	0.002	0.020	0.032

rs7248205	C	0.40	0.014	0.002	-0.111	0.031
rs72671494	T	0.86	-0.017	0.003	0.147	0.046
rs72781699	G	0.80	-0.019	0.003	-0.050	0.038
rs72828890	C	0.87	0.019	0.003	0.065	0.048
rs72834698	G	0.86	0.023	0.003	0.082	0.044
rs749671	G	0.63	0.016	0.002	0.108	0.031
rs7564130	T	0.64	-0.015	0.002	-0.019	0.031
rs7693082	G	0.30	0.015	0.002	0.022	0.033
rs7693703	G	0.91	0.023	0.004	0.020	0.053
rs7700107	A	0.86	-0.021	0.003	-0.219	0.044
rs77215114	A	0.93	0.024	0.004	-0.047	0.062
rs801733	A	0.64	0.017	0.002	0.007	0.031
rs8043253	C	0.57	-0.012	0.002	-0.240	0.030
rs8756	C	0.48	-0.013	0.002	-0.239	0.030
rs9471333	C	0.45	0.013	0.002	-0.009	0.030
rs9563168	G	0.79	0.018	0.003	0.139	0.037
rs9718104	T	0.94	-0.041	0.005	-0.138	0.068
rs973734	C	0.15	0.017	0.003	0.053	0.042
rs9834970	T	0.50	0.013	0.002	0.095	0.030
rs984409	G	0.36	-0.015	0.002	-0.161	0.032
rs9867121	C	0.82	0.019	0.003	-0.043	0.040
rs9902312	T	0.68	0.015	0.002	0.077	0.032
rs9964724	C	0.32	0.018	0.002	0.041	0.032
**DBP**						
rs10041724	T	0.81	0.018	0.003	0.014	0.022
rs10054327	G	0.58	0.017	0.002	-0.098	0.018
rs10145592	C	0.41	-0.015	0.002	-0.025	0.018
rs10189857	A	0.57	-0.020	0.002	-0.028	0.018
rs1022785	G	0.14	0.018	0.003	-0.011	0.025
rs10234444	G	0.82	0.016	0.003	0.013	0.023
rs10246289	A	0.11	0.019	0.003	0.009	0.027
rs1031423	T	0.22	-0.019	0.003	-0.013	0.021
rs10427502	G	0.62	0.014	0.002	-0.056	0.018
rs10737620	T	0.27	0.014	0.002	0.091	0.019
rs10771746	C	0.72	-0.014	0.002	-0.026	0.019
rs10772643	C	0.11	0.025	0.003	0.116	0.028
rs10876864	G	0.43	-0.013	0.002	-0.084	0.018
rs10890123	C	0.77	0.014	0.003	0.024	0.021
rs10932837	C	0.49	-0.013	0.002	-0.046	0.017
rs10940659	A	0.53	-0.013	0.002	0.016	0.018
rs10994943	T	0.58	0.013	0.002	0.060	0.018
rs11130793	C	0.60	0.013	0.002	-0.031	0.018
rs11201422	T	0.67	0.013	0.002	0.024	0.019
rs11218575	C	0.57	0.015	0.002	-0.043	0.018
rs11245482	T	0.61	-0.013	0.002	-0.076	0.018
rs114328297	T	0.78	0.014	0.003	-0.010	0.021
rs1156541	C	0.22	0.014	0.003	-0.001	0.021

rs11654952	T	0.86	-0.017	0.003	-0.025	0.026
rs11657730	C	0.64	0.013	0.002	0.107	0.019
rs11689199	A	0.60	0.018	0.002	-0.044	0.018
rs11714337	G	0.57	0.014	0.002	0.023	0.018
rs11763734	A	0.51	-0.012	0.002	-0.020	0.017
rs11810109	A	0.70	0.016	0.002	-0.080	0.019
rs12105701	C	0.40	-0.013	0.002	0.002	0.018
rs12272012	G	0.96	-0.030	0.005	-0.082	0.043
rs12289262	C	0.73	-0.014	0.002	0.039	0.020
rs1243182	C	0.69	-0.019	0.002	-0.049	0.019
rs12491503	G	0.67	-0.014	0.002	-0.014	0.018
rs12541615	T	0.82	-0.018	0.003	-0.103	0.022
rs12554512	T	0.58	0.021	0.002	-0.001	0.018
rs12725114	G	0.80	0.015	0.003	0.041	0.022
rs1278847	C	0.69	0.016	0.002	0.000	0.019
rs13029509	G	0.52	-0.018	0.002	-0.037	0.017
rs13107325	C	0.93	-0.029	0.004	0.675	0.034
rs138256022	C	0.96	-0.031	0.006	-0.075	0.048
rs1421334	A	0.45	0.017	0.002	0.043	0.018
rs1451533	G	0.72	-0.016	0.002	-0.029	0.020
rs17207890	G	0.66	0.016	0.002	0.035	0.018
rs17379561	A	0.86	-0.025	0.003	0.034	0.025
rs17512836	T	0.97	0.042	0.007	-0.143	0.054
rs17727474	C	0.83	0.018	0.003	-0.030	0.023
rs17789218	T	0.76	0.019	0.003	0.125	0.021
rs2034768	A	0.49	0.015	0.002	0.006	0.017
rs2045147	A	0.45	0.013	0.002	-0.016	0.017
rs2073869	C	0.83	0.019	0.003	-0.030	0.023
rs2164744	T	0.64	-0.013	0.002	0.042	0.018
rs2173650	G	0.85	0.018	0.003	-0.028	0.025
rs2447098	C	0.48	-0.015	0.002	-0.020	0.018
rs2460	G	0.74	-0.015	0.002	-0.061	0.020
rs2616830	G	0.46	0.016	0.002	0.027	0.017
rs262890	A	0.70	-0.019	0.002	-0.019	0.019
rs2717559	A	0.56	0.012	0.002	-0.015	0.018
rs2787374	T	0.41	0.012	0.002	0.007	0.018
rs303753	G	0.65	-0.014	0.002	-0.062	0.018
rs34864022	A	0.93	-0.026	0.004	0.003	0.036
rs35574015	T	0.71	-0.013	0.002	-0.038	0.019
rs374722	G	0.15	0.024	0.003	0.035	0.024
rs3754970	T	0.50	-0.015	0.002	-0.027	0.017
rs3796386	G	0.57	-0.026	0.002	-0.061	0.018
rs42210	G	0.29	-0.014	0.002	-0.009	0.019
rs4334769	G	0.47	0.012	0.002	-0.022	0.017
rs4382592	T	0.30	0.014	0.002	-0.015	0.019
rs4523073	A	0.59	-0.014	0.002	0.015	0.018
rs4577309	A	0.47	0.016	0.002	0.093	0.017

rs4675246	G	0.80	-0.015	0.003	-0.014	0.022
rs4775373	T	0.36	0.013	0.002	0.050	0.018
rs4845364	A	0.50	-0.015	0.002	0.099	0.017
rs4937842	G	0.63	-0.013	0.002	0.019	0.018
rs4973576	C	0.30	-0.015	0.002	0.038	0.019
rs55700114	G	0.71	-0.014	0.002	-0.008	0.019
rs56103247	C	0.94	0.030	0.005	0.047	0.041
rs56858768	G	0.70	-0.015	0.002	-0.016	0.019
rs57585211	T	0.83	-0.017	0.003	-0.020	0.023
rs6131281	C	0.60	0.016	0.002	0.121	0.018
rs6141814	C	0.61	-0.014	0.002	-0.025	0.018
rs62379379	G	0.93	-0.026	0.004	-0.020	0.034
rs62641636	A	0.69	0.014	0.002	-0.002	0.019
rs6472942	T	0.57	-0.013	0.002	0.069	0.018
rs6673341	T	0.47	-0.015	0.002	-0.052	0.018
rs6797840	A	0.46	-0.016	0.002	0.004	0.018
rs6825241	C	0.54	-0.017	0.002	-0.078	0.017
rs6850494	A	0.62	-0.014	0.002	0.028	0.018
rs6905544	A	0.40	-0.019	0.002	-0.064	0.018
rs6973656	A	0.60	-0.013	0.002	0.040	0.018
rs6996198	C	0.84	-0.016	0.003	-0.060	0.024
rs7089973	C	0.62	-0.013	0.002	-0.063	0.018
rs7157001	A	0.74	-0.014	0.002	-0.020	0.021
rs71658797	T	0.88	-0.020	0.003	0.225	0.027
rs7184800	G	0.70	0.017	0.002	0.075	0.019
rs7189927	T	0.36	0.015	0.002	0.002	0.018
rs7248205	C	0.40	0.014	0.002	-0.025	0.018
rs72671494	T	0.86	-0.017	0.003	0.071	0.026
rs72781699	G	0.80	-0.019	0.003	-0.031	0.022
rs72828890	C	0.87	0.019	0.003	0.037	0.028
rs72834698	G	0.86	0.023	0.003	0.052	0.025
rs749671	G	0.63	0.016	0.002	0.088	0.018
rs7564130	T	0.64	-0.015	0.002	-0.027	0.018
rs7693082	G	0.30	0.015	0.002	0.006	0.019
rs7693703	G	0.91	0.023	0.004	0.031	0.030
rs7700107	A	0.86	-0.021	0.003	-0.121	0.026
rs77215114	A	0.93	0.024	0.004	-0.036	0.036
rs801733	A	0.64	0.017	0.002	-0.004	0.018
rs8043253	C	0.57	-0.012	0.002	-0.156	0.018
rs8756	C	0.48	-0.013	0.002	-0.067	0.017
rs9471333	C	0.45	0.013	0.002	-0.032	0.017
rs9563168	G	0.79	0.018	0.003	0.041	0.021
rs9718104	T	0.94	-0.041	0.005	-0.106	0.039
rs973734	C	0.15	0.017	0.003	-0.014	0.024
rs9834970	T	0.50	0.013	0.002	0.007	0.017
rs984409	G	0.36	-0.015	0.002	-0.073	0.018
rs9867121	C	0.82	0.019	0.003	0.020	0.023

rs9902312	T	0.68	0.015	0.002	0.017	0.019
rs9964724	C	0.32	0.018	0.002	0.002	0.019
**PP**						
rs10041724	T	0.81	0.018	0.003	0.023	0.026
rs10054327	G	0.58	0.017	0.002	-0.076	0.021
rs10145592	C	0.41	-0.015	0.002	0.021	0.021
rs10189857	A	0.57	-0.020	0.002	0.049	0.021
rs1022785	G	0.14	0.018	0.003	-0.018	0.029
rs10234444	G	0.82	0.016	0.003	-0.067	0.028
rs10246289	A	0.11	0.019	0.003	0.005	0.032
rs1031423	T	0.22	-0.019	0.003	0.014	0.025
rs10427502	G	0.62	0.014	0.002	0.057	0.021
rs10737620	T	0.27	0.014	0.002	0.074	0.023
rs10771746	C	0.72	-0.014	0.002	0.051	0.023
rs10772643	C	0.11	0.025	0.003	0.113	0.033
rs10876864	G	0.43	-0.013	0.002	-0.036	0.021
rs10890123	C	0.77	0.014	0.003	0.041	0.025
rs10932837	C	0.49	-0.013	0.002	-0.032	0.021
rs10940659	A	0.53	-0.013	0.002	0.018	0.021
rs10994943	T	0.58	0.013	0.002	-0.054	0.021
rs11130793	C	0.60	0.013	0.002	0.013	0.021
rs11201422	T	0.67	0.013	0.002	0.024	0.022
rs11218575	C	0.57	0.015	0.002	-0.035	0.021
rs11245482	T	0.61	-0.013	0.002	-0.017	0.021
rs114328297	T	0.78	0.014	0.003	-0.025	0.025
rs1156541	C	0.22	0.014	0.003	-0.012	0.025
rs11654952	T	0.86	-0.017	0.003	-0.065	0.031
rs11657730	C	0.64	0.013	0.002	0.093	0.023
rs11689199	A	0.60	0.018	0.002	-0.026	0.021
rs11714337	G	0.57	0.014	0.002	0.042	0.021
rs11763734	A	0.51	-0.012	0.002	-0.040	0.021
rs11810109	A	0.70	0.016	0.002	0.011	0.022
rs12105701	C	0.40	-0.013	0.002	0.011	0.021
rs12272012	G	0.96	-0.030	0.005	-0.088	0.051
rs12289262	C	0.73	-0.014	0.002	0.020	0.023
rs1243182	C	0.69	-0.019	0.002	-0.019	0.022
rs12491503	G	0.67	-0.014	0.002	-0.025	0.022
rs12541615	T	0.82	-0.018	0.003	0.004	0.027
rs12554512	T	0.58	0.021	0.002	0.007	0.021
rs12725114	G	0.80	0.015	0.003	0.038	0.025
rs1278847	C	0.69	0.016	0.002	-0.061	0.022
rs13029509	G	0.52	-0.018	0.002	-0.066	0.021
rs13107325	C	0.93	-0.029	0.004	0.253	0.040
rs138256022	C	0.96	-0.031	0.006	-0.125	0.057
rs1421334	A	0.45	0.017	0.002	0.034	0.021
rs1451533	G	0.72	-0.016	0.002	0.013	0.023
rs17207890	G	0.66	0.016	0.002	0.009	0.022

rs17379561	A	0.86	-0.025	0.003	0.050	0.029
rs17512836	T	0.97	0.042	0.007	-0.061	0.063
rs17727474	C	0.83	0.018	0.003	-0.037	0.028
rs17789218	T	0.76	0.019	0.003	-0.005	0.024
rs2034768	A	0.49	0.015	0.002	-0.015	0.021
rs2045147	A	0.45	0.013	0.002	0.022	0.021
rs2073869	C	0.83	0.019	0.003	-0.074	0.027
rs2164744	T	0.64	-0.013	0.002	0.033	0.021
rs2173650	G	0.85	0.018	0.003	-0.027	0.029
rs2447098	C	0.48	-0.015	0.002	-0.040	0.021
rs2460	G	0.74	-0.015	0.002	-0.047	0.024
rs2616830	G	0.46	0.016	0.002	-0.007	0.020
rs262890	A	0.70	-0.019	0.002	-0.006	0.022
rs2717559	A	0.56	0.012	0.002	-0.026	0.021
rs2787374	T	0.41	0.012	0.002	-0.044	0.021
rs303753	G	0.65	-0.014	0.002	-0.016	0.022
rs34864022	A	0.93	-0.026	0.004	0.066	0.042
rs35574015	T	0.71	-0.013	0.002	-0.038	0.023
rs374722	G	0.15	0.024	0.003	0.011	0.029
rs3754970	T	0.50	-0.015	0.002	-0.049	0.021
rs3796386	G	0.57	-0.026	0.002	-0.012	0.021
rs42210	G	0.29	-0.014	0.002	0.041	0.023
rs4334769	G	0.47	0.012	0.002	-0.034	0.021
rs4382592	T	0.30	0.014	0.002	-0.011	0.022
rs4523073	A	0.59	-0.014	0.002	0.052	0.021
rs4577309	A	0.47	0.016	0.002	0.069	0.021
rs4675246	G	0.80	-0.015	0.003	0.048	0.026
rs4775373	T	0.36	0.013	0.002	0.030	0.021
rs4845364	A	0.50	-0.015	0.002	-0.051	0.021
rs4937842	G	0.63	-0.013	0.002	0.027	0.021
rs4973576	C	0.30	-0.015	0.002	0.043	0.023
rs55700114	G	0.71	-0.014	0.002	0.015	0.022
rs56103247	C	0.94	0.030	0.005	-0.120	0.049
rs56858768	G	0.70	-0.015	0.002	-0.014	0.022
rs57585211	T	0.83	-0.017	0.003	-0.007	0.027
rs6131281	C	0.60	0.016	0.002	0.023	0.021
rs6141814	C	0.61	-0.014	0.002	-0.112	0.021
rs62379379	G	0.93	-0.026	0.004	-0.156	0.040
rs62641636	A	0.69	0.014	0.002	0.017	0.023
rs6472942	T	0.57	-0.013	0.002	0.181	0.021
rs6673341	T	0.47	-0.015	0.002	-0.073	0.021
rs6797840	A	0.46	-0.016	0.002	0.025	0.021
rs6825241	C	0.54	-0.017	0.002	-0.026	0.021
rs6850494	A	0.62	-0.014	0.002	-0.028	0.021
rs6905544	A	0.40	-0.019	0.002	-0.058	0.021
rs6973656	A	0.60	-0.013	0.002	0.088	0.021
rs6996198	C	0.84	-0.016	0.003	-0.112	0.028

rs7089973	C	0.62	-0.013	0.002	-0.055	0.021
rs7157001	A	0.74	-0.014	0.002	0.000	0.025
rs71658797	T	0.88	-0.020	0.003	-0.008	0.032
rs7184800	G	0.70	0.017	0.002	0.031	0.022
rs7189927	T	0.36	0.015	0.002	0.015	0.022
rs7248205	C	0.40	0.014	0.002	-0.090	0.021
rs72671494	T	0.86	-0.017	0.003	0.087	0.031
rs72781699	G	0.80	-0.019	0.003	-0.007	0.026
rs72828890	C	0.87	0.019	0.003	0.033	0.033
rs72834698	G	0.86	0.023	0.003	0.039	0.030
rs749671	G	0.63	0.016	0.002	0.009	0.021
rs7564130	T	0.64	-0.015	0.002	0.002	0.021
rs7693082	G	0.30	0.015	0.002	0.012	0.022
rs7693703	G	0.91	0.023	0.004	-0.011	0.036
rs7700107	A	0.86	-0.021	0.003	-0.094	0.030
rs77215114	A	0.93	0.024	0.004	0.020	0.042
rs801733	A	0.64	0.017	0.002	0.006	0.021
rs8043253	C	0.57	-0.012	0.002	-0.087	0.021
rs8756	C	0.48	-0.013	0.002	-0.179	0.020
rs9471333	C	0.45	0.013	0.002	0.028	0.021
rs9563168	G	0.79	0.018	0.003	0.093	0.025
rs9718104	T	0.94	-0.041	0.005	-0.046	0.046
rs973734	C	0.15	0.017	0.003	0.058	0.028
rs9834970	T	0.50	0.013	0.002	0.080	0.021
rs984409	G	0.36	-0.015	0.002	-0.086	0.021
rs9867121	C	0.82	0.019	0.003	-0.069	0.027
rs9902312	T	0.68	0.015	0.002	0.057	0.022
rs9964724	C	0.32	0.018	0.002	0.042	0.022
**HDL-C**						
rs7089973	C	0.62	-0.013	0.002	-0.014	0.005
rs11245482	T	0.61	-0.013	0.002	0.009	0.005
rs17727474	C	0.83	0.018	0.003	0.000	0.006
rs1243182	C	0.69	-0.019	0.002	0.008	0.005
rs2045147	A	0.45	0.013	0.002	-0.007	0.005
rs11201422	T	0.67	0.013	0.002	-0.008	0.005
rs11218575	C	0.57	0.015	0.002	-0.011	0.005
rs12289262	C	0.73	-0.014	0.002	0.004	0.005
rs4845364	A	0.50	-0.015	0.002	0.003	0.005
rs801733	A	0.64	0.017	0.002	-0.011	0.004
rs6673341	T	0.47	-0.015	0.002	-0.004	0.005
rs17207890	G	0.66	0.016	0.002	0.006	0.005
rs10876864	G	0.43	-0.013	0.002	0.005	0.005
rs8756	C	0.48	-0.013	0.002	-0.002	0.003
rs9563168	G	0.79	0.018	0.003	-0.014	0.006
rs9528023	C	0.72	0.013	0.002	-0.001	0.006
rs1022785	G	0.14	0.018	0.003	-0.010	0.007
rs8043253	C	0.57	-0.012	0.002	0.003	0.005

rs2460	G	0.74	-0.015	0.002	0.005	0.006
rs7189927	T	0.36	0.015	0.002	-0.012	0.004
rs749671	G	0.63	0.016	0.002	-0.007	0.004
rs2447098	C	0.48	-0.015	0.002	-0.008	0.005
rs11654952	T	0.86	-0.017	0.003	-0.015	0.007
rs2584597	T	0.66	0.015	0.002	-0.006	0.005
rs9902312	T	0.68	0.015	0.002	0.005	0.005
rs303753	G	0.65	-0.014	0.002	0.005	0.005
rs11081851	G	0.44	-0.013	0.002	0.005	0.005
rs9964724	C	0.32	0.018	0.002	0.000	0.005
rs1156541	C	0.22	0.014	0.003	-0.005	0.006
rs6124476	T	0.59	0.012	0.002	-0.006	0.005
rs11689199	A	0.60	0.018	0.002	-0.008	0.005
rs1451533	G	0.72	-0.016	0.002	0.006	0.005
rs2971640	G	0.51	-0.013	0.002	0.001	0.005
rs374722	G	0.15	0.024	0.003	0.003	0.007
rs1913808	G	0.71	0.015	0.002	0.000	0.005
rs263771	C	0.77	-0.017	0.003	-0.009	0.006
rs4675246	G	0.80	-0.015	0.003	-0.009	0.006
rs13029509	G	0.52	-0.018	0.002	0.017	0.005
rs10932837	C	0.49	-0.013	0.002	-0.003	0.005
rs12105701	C	0.40	-0.013	0.002	0.008	0.005
rs7564130	T	0.64	-0.015	0.002	0.000	0.005
rs11125515	C	0.73	-0.014	0.002	-0.006	0.005
rs10189857	A	0.57	-0.020	0.002	0.003	0.005
rs12632778	T	0.95	-0.028	0.005	0.012	0.012
rs12491503	G	0.67	-0.014	0.002	0.006	0.005
rs9834970	T	0.50	0.013	0.002	-0.004	0.005
rs3796386	G	0.57	-0.026	0.002	0.015	0.005
rs6797840	A	0.46	-0.016	0.002	0.003	0.005
rs13107325	C	0.93	-0.029	0.004	0.071	0.008
rs7693703	G	0.91	0.023	0.004	-0.002	0.008
rs4334769	G	0.47	0.012	0.002	0.001	0.005
rs6825241	C	0.54	-0.017	0.002	0.001	0.005
rs7700107	A	0.86	-0.021	0.003	0.018	0.007
rs77215114	A	0.93	0.024	0.004	0.002	0.009
rs10041724	T	0.81	0.018	0.003	-0.020	0.006
rs255292	C	0.40	-0.012	0.002	0.000	0.005
rs10940659	A	0.53	-0.013	0.002	0.009	0.005
rs1031423	T	0.22	-0.019	0.003	0.002	0.006
rs17789218	T	0.76	0.019	0.003	-0.004	0.004
rs6905544	A	0.40	-0.019	0.002	0.007	0.005
rs7788008	G	0.57	0.013	0.002	-0.012	0.005
rs11763734	A	0.51	-0.012	0.002	0.002	0.005
rs10234444	G	0.82	0.016	0.003	0.005	0.007
rs973734	C	0.15	0.017	0.003	0.000	0.007
rs3137	G	0.85	0.017	0.003	-0.011	0.007

rs1421334	A	0.45	0.017	0.002	-0.013	0.005
rs6996198	C	0.84	-0.016	0.003	0.004	0.007
rs6472942	T	0.57	-0.013	0.002	0.000	0.005
rs2787374	T	0.41	0.012	0.002	-0.003	0.005
rs4382592	T	0.30	0.014	0.002	0.004	0.005
rs2073869	C	0.83	0.019	0.003	0.000	0.006
rs2291681	G	0.58	0.013	0.002	-0.002	0.005
rs2616830	G	0.46	0.016	0.002	0.002	0.005
rs2791574	T	0.66	-0.013	0.002	0.009	0.005
rs7609	A	0.23	0.015	0.003	-0.007	0.006
rs10737620	T	0.27	0.014	0.002	0.004	0.005
rs17379561	A	0.86	-0.025	0.003	0.009	0.007
rs6031440	A	0.40	0.012	0.002	-0.005	0.005
rs631130	T	0.37	-0.014	0.002	-0.008	0.006
rs10739499	C	0.34	0.014	0.002	-0.001	0.005
rs10145592	C	0.41	-0.015	0.002	0.004	0.005
rs4937842	G	0.63	-0.013	0.002	0.001	0.005
**LDL-C**						
rs7089973	C	0.62	-0.013	0.002	-0.001	0.006
rs11245482	T	0.61	-0.013	0.002	0.003	0.006
rs17727474	C	0.83	0.018	0.003	0.017	0.007
rs1243182	C	0.69	-0.019	0.002	0.005	0.006
rs2045147	A	0.45	0.013	0.002	0.005	0.005
rs11201422	T	0.67	0.013	0.002	0.020	0.006
rs11218575	C	0.57	0.015	0.002	0.000	0.005
rs12289262	C	0.73	-0.014	0.002	-0.004	0.006
rs4845364	A	0.50	-0.015	0.002	-0.006	0.005
rs801733	A	0.64	0.017	0.002	0.002	0.004
rs6673341	T	0.47	-0.015	0.002	-0.001	0.005
rs17207890	G	0.66	0.016	0.002	0.013	0.006
rs10876864	G	0.43	-0.013	0.002	-0.001	0.005
rs8756	C	0.48	-0.013	0.002	0.001	0.004
rs9563168	G	0.79	0.018	0.003	0.007	0.006
rs9528023	C	0.72	0.013	0.002	0.000	0.006
rs1022785	G	0.14	0.018	0.003	0.002	0.007
rs8043253	C	0.57	-0.012	0.002	0.005	0.005
rs2460	G	0.74	-0.015	0.002	0.003	0.006
rs7189927	T	0.36	0.015	0.002	-0.007	0.004
rs749671	G	0.63	0.016	0.002	0.015	0.004
rs2447098	C	0.48	-0.015	0.002	-0.016	0.006
rs11654952	T	0.86	-0.017	0.003	0.008	0.008
rs2584597	T	0.66	0.015	0.002	0.000	0.005
rs9902312	T	0.68	0.015	0.002	-0.004	0.005
rs303753	G	0.65	-0.014	0.002	-0.008	0.006
rs11081851	G	0.44	-0.013	0.002	0.007	0.006
rs9964724	C	0.32	0.018	0.002	0.002	0.006
rs1156541	C	0.22	0.014	0.003	0.012	0.006

rs6124476	T	0.59	0.012	0.002	-0.001	0.005
rs11689199	A	0.60	0.018	0.002	-0.003	0.005
rs1451533	G	0.72	-0.016	0.002	-0.004	0.006
rs2971640	G	0.51	-0.013	0.002	0.007	0.005
rs374722	G	0.15	0.024	0.003	-0.002	0.007
rs1913808	G	0.71	0.015	0.002	-0.012	0.006
rs263771	C	0.77	-0.017	0.003	-0.002	0.007
rs4675246	G	0.80	-0.015	0.003	-0.001	0.007
rs13029509	G	0.52	-0.018	0.002	-0.004	0.005
rs10932837	C	0.49	-0.013	0.002	0.002	0.005
rs12105701	C	0.40	-0.013	0.002	-0.009	0.006
rs7564130	T	0.64	-0.015	0.002	0.009	0.005
rs11125515	C	0.73	-0.014	0.002	-0.005	0.006
rs10189857	A	0.57	-0.020	0.002	-0.002	0.005
rs12632778	T	0.95	-0.028	0.005	0.016	0.013
rs12491503	G	0.67	-0.014	0.002	0.001	0.005
rs9834970	T	0.50	0.013	0.002	-0.004	0.005
rs3796386	G	0.57	-0.026	0.002	0.004	0.005
rs6797840	A	0.46	-0.016	0.002	0.013	0.005
rs13107325	C	0.93	-0.029	0.004	0.016	0.008
rs7693703	G	0.91	0.023	0.004	-0.001	0.009
rs4334769	G	0.47	0.012	0.002	0.010	0.005
rs6825241	C	0.54	-0.017	0.002	0.005	0.005
rs7700107	A	0.86	-0.021	0.003	-0.006	0.008
rs77215114	A	0.93	0.024	0.004	0.015	0.009
rs10041724	T	0.81	0.018	0.003	0.002	0.007
rs255292	C	0.40	-0.012	0.002	0.004	0.005
rs10940659	A	0.53	-0.013	0.002	-0.013	0.005
rs1031423	T	0.22	-0.019	0.003	-0.005	0.007
rs17789218	T	0.76	0.019	0.003	0.024	0.004
rs6905544	A	0.40	-0.019	0.002	-0.012	0.005
rs7788008	G	0.57	0.013	0.002	0.005	0.005
rs11763734	A	0.51	-0.012	0.002	0.002	0.005
rs10234444	G	0.82	0.016	0.003	0.001	0.007
rs973734	C	0.15	0.017	0.003	0.013	0.007
rs3137	G	0.85	0.017	0.003	-0.001	0.007
rs1421334	A	0.45	0.017	0.002	0.005	0.005
rs6996198	C	0.84	-0.016	0.003	-0.007	0.007
rs6472942	T	0.57	-0.013	0.002	0.001	0.005
rs2787374	T	0.41	0.012	0.002	0.003	0.005
rs4382592	T	0.30	0.014	0.002	0.004	0.006
rs2073869	C	0.83	0.019	0.003	-0.001	0.007
rs2291681	G	0.58	0.013	0.002	-0.001	0.005
rs2616830	G	0.46	0.016	0.002	-0.007	0.005
rs2791574	T	0.66	-0.013	0.002	-0.006	0.006
rs7609	A	0.23	0.015	0.003	-0.004	0.006
rs10737620	T	0.27	0.014	0.002	-0.013	0.006

rs17379561	A	0.86	-0.025	0.003	-0.007	0.008
rs6031440	A	0.40	0.012	0.002	0.010	0.005
rs631130	T	0.37	-0.014	0.002	0.013	0.007
rs10739499	C	0.34	0.014	0.002	-0.014	0.005
rs10145592	C	0.41	-0.015	0.002	0.006	0.005
rs4937842	G	0.63	-0.013	0.002	0.003	0.005
**TC**						
rs7089973	C	0.62	-0.013	0.002	-0.005	0.006
rs11245482	T	0.61	-0.013	0.002	0.007	0.005
rs17727474	C	0.83	0.018	0.003	0.023	0.007
rs1243182	C	0.69	-0.019	0.002	0.001	0.005
rs2045147	A	0.45	0.013	0.002	0.004	0.005
rs11201422	T	0.67	0.013	0.002	0.013	0.006
rs11218575	C	0.57	0.015	0.002	-0.002	0.005
rs12289262	C	0.73	-0.014	0.002	-0.001	0.006
rs4845364	A	0.50	-0.015	0.002	-0.001	0.005
rs801733	A	0.64	0.017	0.002	-0.002	0.004
rs6673341	T	0.47	-0.015	0.002	-0.003	0.005
rs17207890	G	0.66	0.016	0.002	0.016	0.006
rs10876864	G	0.43	-0.013	0.002	0.002	0.005
rs8756	C	0.48	-0.013	0.002	-0.005	0.004
rs9563168	G	0.79	0.018	0.003	0.005	0.006
rs9528023	C	0.72	0.013	0.002	-0.001	0.006
rs1022785	G	0.14	0.018	0.003	0.003	0.007
rs8043253	C	0.57	-0.012	0.002	0.005	0.005
rs2460	G	0.74	-0.015	0.002	0.001	0.006
rs7189927	T	0.36	0.015	0.002	-0.013	0.004
rs749671	G	0.63	0.016	0.002	0.017	0.004
rs2447098	C	0.48	-0.015	0.002	-0.019	0.006
rs11654952	T	0.86	-0.017	0.003	0.000	0.008
rs2584597	T	0.66	0.015	0.002	-0.007	0.005
rs9902312	T	0.68	0.015	0.002	-0.002	0.005
rs303753	G	0.65	-0.014	0.002	-0.009	0.005
rs11081851	G	0.44	-0.013	0.002	0.006	0.005
rs9964724	C	0.32	0.018	0.002	0.001	0.006
rs1156541	C	0.22	0.014	0.003	0.005	0.006
rs6124476	T	0.59	0.012	0.002	-0.002	0.005
rs11689199	A	0.60	0.018	0.002	-0.007	0.005
rs1451533	G	0.72	-0.016	0.002	-0.002	0.006
rs2971640	G	0.51	-0.013	0.002	0.006	0.005
rs374722	G	0.15	0.024	0.003	0.004	0.007
rs1913808	G	0.71	0.015	0.002	-0.010	0.006
rs263771	C	0.77	-0.017	0.003	-0.001	0.007
rs4675246	G	0.80	-0.015	0.003	-0.002	0.007
rs13029509	G	0.52	-0.018	0.002	-0.002	0.005
rs10932837	C	0.49	-0.013	0.002	0.000	0.005
rs12105701	C	0.40	-0.013	0.002	-0.007	0.005

rs7564130	T	0.64	-0.015	0.002	0.011	0.005
rs11125515	C	0.73	-0.014	0.002	-0.003	0.006
rs10189857	A	0.57	-0.020	0.002	-0.004	0.005
rs12632778	T	0.95	-0.028	0.005	0.010	0.012
rs12491503	G	0.67	-0.014	0.002	-0.002	0.005
rs9834970	T	0.50	0.013	0.002	-0.006	0.005
rs3796386	G	0.57	-0.026	0.002	0.011	0.005
rs6797840	A	0.46	-0.016	0.002	0.007	0.005
rs13107325	C	0.93	-0.029	0.004	0.033	0.008
rs7693703	G	0.91	0.023	0.004	0.000	0.009
rs4334769	G	0.47	0.012	0.002	0.008	0.005
rs6825241	C	0.54	-0.017	0.002	0.004	0.005
rs7700107	A	0.86	-0.021	0.003	-0.001	0.008
rs77215114	A	0.93	0.024	0.004	0.011	0.009
rs10041724	T	0.81	0.018	0.003	-0.003	0.006
rs255292	C	0.40	-0.012	0.002	0.005	0.005
rs10940659	A	0.53	-0.013	0.002	-0.009	0.005
rs1031423	T	0.22	-0.019	0.003	-0.003	0.007
rs17789218	T	0.76	0.019	0.003	0.020	0.004
rs6905544	A	0.40	-0.019	0.002	-0.010	0.005
rs7788008	G	0.57	0.013	0.002	0.002	0.005
rs11763734	A	0.51	-0.012	0.002	0.004	0.005
rs10234444	G	0.82	0.016	0.003	0.003	0.007
rs973734	C	0.15	0.017	0.003	0.014	0.007
rs3137	G	0.85	0.017	0.003	0.003	0.007
rs1421334	A	0.45	0.017	0.002	0.001	0.005
rs6996198	C	0.84	-0.016	0.003	-0.005	0.007
rs6472942	T	0.57	-0.013	0.002	0.000	0.005
rs2787374	T	0.41	0.012	0.002	-0.006	0.005
rs4382592	T	0.30	0.014	0.002	0.006	0.006
rs2073869	C	0.83	0.019	0.003	-0.002	0.007
rs2291681	G	0.58	0.013	0.002	-0.006	0.005
rs2616830	G	0.46	0.016	0.002	-0.007	0.005
rs2791574	T	0.66	-0.013	0.002	-0.005	0.005
rs7609	A	0.23	0.015	0.003	-0.005	0.006
rs10737620	T	0.27	0.014	0.002	-0.010	0.006
rs17379561	A	0.86	-0.025	0.003	-0.004	0.007
rs6031440	A	0.40	0.012	0.002	0.009	0.005
rs631130	T	0.37	-0.014	0.002	0.008	0.007
rs10739499	C	0.34	0.014	0.002	-0.016	0.005
rs10145592	C	0.41	-0.015	0.002	0.010	0.005
rs4937842	G	0.63	-0.013	0.002	-0.001	0.005
**TG**						
rs7089973	C	0.62	-0.013	0.002	-0.002	0.005
rs11245482	T	0.61	-0.013	0.002	-0.002	0.005
rs17727474	C	0.83	0.018	0.003	0.009	0.006
rs1243182	C	0.69	-0.019	0.002	-0.007	0.005

rs2045147	A	0.45	0.013	0.002	0.007	0.005
rs11201422	T	0.67	0.013	0.002	0.004	0.005
rs11218575	C	0.57	0.015	0.002	0.008	0.005
rs12289262	C	0.73	-0.014	0.002	0.006	0.005
rs4845364	A	0.50	-0.015	0.002	-0.005	0.005
rs801733	A	0.64	0.017	0.002	0.007	0.004
rs6673341	T	0.47	-0.015	0.002	0.000	0.005
rs17207890	G	0.66	0.016	0.002	0.010	0.005
rs10876864	G	0.43	-0.013	0.002	-0.006	0.005
rs8756	C	0.48	-0.013	0.002	-0.003	0.003
rs9563168	G	0.79	0.018	0.003	0.014	0.006
rs9528023	C	0.72	0.013	0.002	-0.009	0.006
rs1022785	G	0.14	0.018	0.003	0.010	0.007
rs8043253	C	0.57	-0.012	0.002	-0.003	0.005
rs2460	G	0.74	-0.015	0.002	-0.012	0.006
rs7189927	T	0.36	0.015	0.002	-0.004	0.004
rs749671	G	0.63	0.016	0.002	0.021	0.003
rs2447098	C	0.48	-0.015	0.002	0.004	0.005
rs11654952	T	0.86	-0.017	0.003	0.007	0.007
rs2584597	T	0.66	0.015	0.002	0.005	0.005
rs9902312	T	0.68	0.015	0.002	0.002	0.005
rs303753	G	0.65	-0.014	0.002	-0.007	0.005
rs11081851	G	0.44	-0.013	0.002	0.000	0.005
rs9964724	C	0.32	0.018	0.002	0.007	0.005
rs1156541	C	0.22	0.014	0.003	0.005	0.006
rs6124476	T	0.59	0.012	0.002	0.008	0.005
rs11689199	A	0.60	0.018	0.002	-0.003	0.005
rs1451533	G	0.72	-0.016	0.002	-0.004	0.005
rs2971640	G	0.51	-0.013	0.002	0.002	0.005
rs374722	G	0.15	0.024	0.003	-0.002	0.006
rs1913808	G	0.71	0.015	0.002	0.003	0.005
rs263771	C	0.77	-0.017	0.003	0.006	0.006
rs4675246	G	0.80	-0.015	0.003	0.006	0.006
rs13029509	G	0.52	-0.018	0.002	-0.014	0.005
rs10932837	C	0.49	-0.013	0.002	0.006	0.005
rs12105701	C	0.40	-0.013	0.002	-0.003	0.005
rs7564130	T	0.64	-0.015	0.002	0.002	0.005
rs11125515	C	0.73	-0.014	0.002	0.006	0.005
rs10189857	A	0.57	-0.020	0.002	-0.004	0.005
rs12632778	T	0.95	-0.028	0.005	-0.003	0.011
rs12491503	G	0.67	-0.014	0.002	-0.005	0.005
rs9834970	T	0.50	0.013	0.002	0.001	0.005
rs3796386	G	0.57	-0.026	0.002	-0.002	0.005
rs6797840	A	0.46	-0.016	0.002	0.001	0.005
rs13107325	C	0.93	-0.029	0.004	-0.031	0.008
rs7693703	G	0.91	0.023	0.004	-0.001	0.008
rs4334769	G	0.47	0.012	0.002	-0.003	0.005

rs6825241	C	0.54	-0.017	0.002	-0.005	0.005
rs7700107	A	0.86	-0.021	0.003	-0.018	0.007
rs77215114	A	0.93	0.024	0.004	0.009	0.009
rs10041724	T	0.81	0.018	0.003	0.005	0.006
rs255292	C	0.40	-0.012	0.002	0.005	0.005
rs10940659	A	0.53	-0.013	0.002	-0.008	0.005
rs1031423	T	0.22	-0.019	0.003	0.003	0.006
rs17789218	T	0.76	0.019	0.003	0.006	0.004
rs6905544	A	0.40	-0.019	0.002	-0.006	0.005
rs7788008	G	0.57	0.013	0.002	0.007	0.005
rs11763734	A	0.51	-0.012	0.002	0.003	0.005
rs10234444	G	0.82	0.016	0.003	-0.003	0.007
rs973734	C	0.15	0.017	0.003	0.000	0.006
rs3137	G	0.85	0.017	0.003	0.013	0.006
rs1421334	A	0.45	0.017	0.002	-0.004	0.005
rs6996198	C	0.84	-0.016	0.003	-0.009	0.006
rs6472942	T	0.57	-0.013	0.002	-0.004	0.005
rs2787374	T	0.41	0.012	0.002	-0.011	0.005
rs4382592	T	0.30	0.014	0.002	0.008	0.005
rs2073869	C	0.83	0.019	0.003	-0.003	0.006
rs2291681	G	0.58	0.013	0.002	-0.001	0.005
rs2616830	G	0.46	0.016	0.002	-0.002	0.005
rs2791574	T	0.66	-0.013	0.002	-0.006	0.005
rs7609	A	0.23	0.015	0.003	0.002	0.006
rs10737620	T	0.27	0.014	0.002	-0.001	0.005
rs17379561	A	0.86	-0.025	0.003	-0.009	0.007
rs6031440	A	0.40	0.012	0.002	0.011	0.005
rs631130	T	0.37	-0.014	0.002	0.010	0.006
rs10739499	C	0.34	0.014	0.002	0.000	0.005
rs10145592	C	0.41	-0.015	0.002	0.001	0.005
rs4937842	G	0.63	-0.013	0.002	-0.001	0.005
**HbA1c**						
rs631130	T	0.37	-0.014	0.002	0.002	0.003
rs17379561	A	0.86	-0.025	0.003	0.003	0.003
rs4845364	A	0.50	-0.015	0.002	0.000	0.002
rs6673341	T	0.47	-0.015	0.002	-0.002	0.002
rs10737620	T	0.27	0.014	0.002	-0.001	0.002
rs12105701	C	0.40	-0.013	0.002	-0.001	0.002
rs7564130	T	0.64	-0.015	0.002	-0.002	0.002
rs11125515	C	0.73	-0.014	0.002	0.000	0.002
rs10189857	A	0.57	-0.020	0.002	-0.002	0.002
rs11689199	A	0.60	0.018	0.002	-0.001	0.002
rs1451533	G	0.72	-0.016	0.002	-0.001	0.002
rs2971640	G	0.51	-0.013	0.002	0.000	0.002
rs374722	G	0.15	0.024	0.003	-0.002	0.003
rs1913808	G	0.71	0.015	0.002	-0.002	0.002
rs263771	C	0.77	-0.017	0.003	0.002	0.002

rs4675246	G	0.80	-0.015	0.003	0.005	0.003
rs13029509	G	0.52	-0.018	0.002	-0.001	0.002
rs10932837	C	0.49	-0.013	0.002	0.002	0.002
rs9834970	T	0.50	0.013	0.002	-0.002	0.002
rs3796386	G	0.57	-0.026	0.002	0.002	0.002
rs11130793	C	0.60	0.013	0.002	-0.001	0.005
rs12632778	T	0.95	-0.028	0.005	0.001	0.004
rs12491503	G	0.67	-0.014	0.002	0.001	0.002
rs7700107	A	0.86	-0.021	0.003	-0.004	0.003
rs13107325	C	0.93	-0.029	0.004	0.000	0.003
rs7693703	G	0.91	0.023	0.004	0.004	0.003
rs4334769	G	0.47	0.012	0.002	0.001	0.002
rs6825241	C	0.54	-0.017	0.002	-0.003	0.002
rs10940659	A	0.53	-0.013	0.002	0.000	0.002
rs1031423	T	0.22	-0.019	0.003	-0.003	0.002
rs10041724	T	0.81	0.018	0.003	0.000	0.002
rs255292	C	0.40	-0.012	0.002	0.003	0.002
rs6905544	A	0.40	-0.019	0.002	0.000	0.002
rs17789218	T	0.76	0.019	0.003	0.000	0.002
rs10234444	G	0.82	0.016	0.003	-0.004	0.003
rs6973656	A	0.60	-0.013	0.002	0.000	0.002
rs973734	C	0.15	0.017	0.003	0.000	0.003
rs3137	G	0.85	0.017	0.003	-0.005	0.003
rs7788008	G	0.57	0.013	0.002	0.003	0.002
rs11763734	A	0.51	-0.012	0.002	0.002	0.002
rs1421334	A	0.45	0.017	0.002	-0.001	0.002
rs6996198	C	0.84	-0.016	0.003	0.002	0.002
rs6472942	T	0.57	-0.013	0.002	0.000	0.002
rs2616830	G	0.46	0.016	0.002	0.002	0.002
rs2291681	G	0.58	0.013	0.002	-0.001	0.002
rs2791574	T	0.66	-0.013	0.002	-0.001	0.002
rs2787374	T	0.41	0.012	0.002	0.001	0.002
rs10739499	C	0.34	0.014	0.002	0.001	0.002
rs4382592	T	0.30	0.014	0.002	0.003	0.002
rs2073869	C	0.83	0.019	0.003	0.002	0.002
rs1243182	C	0.69	-0.019	0.002	0.002	0.002
rs2045147	A	0.45	0.013	0.002	0.003	0.002
rs11201422	T	0.67	0.013	0.002	0.003	0.002
rs7089973	C	0.62	-0.013	0.002	-0.001	0.002
rs11245482	T	0.61	-0.013	0.002	0.000	0.002
rs17727474	C	0.83	0.018	0.003	-0.004	0.002
rs12289262	C	0.73	-0.014	0.002	-0.001	0.002
rs801733	A	0.64	0.017	0.002	-0.001	0.002
rs17207890	G	0.66	0.016	0.002	0.002	0.002
rs11218575	C	0.57	0.015	0.002	0.000	0.002
rs4937842	G	0.63	-0.013	0.002	0.001	0.002
rs10876864	G	0.43	-0.013	0.002	-0.003	0.002

rs8756	C	0.48	-0.013	0.002	-0.001	0.002
rs9563168	G	0.79	0.018	0.003	0.005	0.002
rs9528023	C	0.72	0.013	0.002	0.000	0.002
rs1022785	G	0.14	0.018	0.003	0.003	0.003
rs10145592	C	0.41	-0.015	0.002	-0.001	0.002
rs8043253	C	0.57	-0.012	0.002	0.000	0.002
rs2460	G	0.74	-0.015	0.002	0.000	0.002
rs7189927	T	0.36	0.015	0.002	0.004	0.002
rs749671	G	0.63	0.016	0.002	0.002	0.002
rs2447098	C	0.48	-0.015	0.002	-0.003	0.002
rs11654952	T	0.86	-0.017	0.003	0.001	0.003
rs2584597	T	0.66	0.015	0.002	0.004	0.002
rs9902312	T	0.68	0.015	0.002	0.003	0.002
rs303753	G	0.65	-0.014	0.002	-0.002	0.002
rs11081851	G	0.44	-0.013	0.002	-0.001	0.002
rs9964724	C	0.32	0.018	0.002	0.004	0.002
rs1156541	C	0.22	0.014	0.003	0.002	0.002
rs6124476	T	0.59	0.012	0.002	0.000	0.002
rs6031440	A	0.40	0.012	0.002	-0.001	0.002
rs7609	A	0.23	0.015	0.003	0.000	0.002
**ISI**						
rs631130	T	0.37	-0.014	0.002	-0.081	0.091
rs17379561	A	0.86	-0.025	0.003	0.010	0.097
rs4845364	A	0.50	-0.015	0.002	0.041	0.077
rs6673341	T	0.47	-0.015	0.002	-0.056	0.078
rs10737620	T	0.27	0.014	0.002	0.021	0.087
rs12105701	C	0.40	-0.013	0.002	0.019	0.087
rs7564130	T	0.64	-0.015	0.002	-0.120	0.077
rs11125515	C	0.73	-0.014	0.002	0.140	0.081
rs10189857	A	0.57	-0.020	0.002	0.093	0.075
rs11689199	A	0.60	0.018	0.002	-0.007	0.078
rs1451533	G	0.72	-0.016	0.002	0.035	0.084
rs2971640	G	0.51	-0.013	0.002	0.007	0.075
rs374722	G	0.15	0.024	0.003	-0.035	0.099
rs1913808	G	0.71	0.015	0.002	-0.110	0.077
rs263771	C	0.77	-0.017	0.003	-0.052	0.095
rs4675246	G	0.80	-0.015	0.003	0.044	0.093
rs13029509	G	0.52	-0.018	0.002	-0.094	0.077
rs10932837	C	0.49	-0.013	0.002	-0.004	0.076
rs9834970	T	0.50	0.013	0.002	-0.074	0.074
rs3796386	G	0.57	-0.026	0.002	0.065	0.079
rs12632778	T	0.95	-0.028	0.005	-0.160	0.180
rs12491503	G	0.67	-0.014	0.002	0.003	0.079
rs7700107	A	0.86	-0.021	0.003	-0.066	0.130
rs13107325	C	0.93	-0.029	0.004	-0.250	0.190
rs7693703	G	0.91	0.023	0.004	-0.073	0.130
rs4334769	G	0.47	0.012	0.002	0.110	0.079

rs6825241	C	0.54	-0.017	0.002	0.032	0.075
rs10940659	A	0.53	-0.013	0.002	-0.110	0.075
rs1031423	T	0.22	-0.019	0.003	0.170	0.110
rs10041724	T	0.81	0.018	0.003	0.070	0.095
rs255292	C	0.40	-0.012	0.002	0.070	0.074
rs6905544	A	0.40	-0.019	0.002	0.040	0.077
rs17789218	T	0.76	0.019	0.003	-0.006	0.084
rs10234444	G	0.82	0.016	0.003	0.024	0.120
rs6973656	A	0.60	-0.013	0.002	0.043	0.077
rs973734	C	0.15	0.017	0.003	-0.150	0.098
rs3137	G	0.85	0.017	0.003	-0.130	0.099
rs7788008	G	0.57	0.013	0.002	-0.014	0.076
rs11763734	A	0.51	-0.012	0.002	-0.120	0.074
rs1421334	A	0.45	0.017	0.002	0.009	0.073
rs6996198	C	0.84	-0.016	0.003	0.210	0.096
rs6472942	T	0.57	-0.013	0.002	-0.057	0.076
rs2616830	G	0.46	0.016	0.002	-0.100	0.083
rs2291681	G	0.58	0.013	0.002	0.093	0.086
rs2791574	T	0.66	-0.013	0.002	0.031	0.086
rs2787374	T	0.41	0.012	0.002	-0.077	0.082
rs10739499	C	0.34	0.014	0.002	-0.016	0.086
rs4382592	T	0.30	0.014	0.002	0.065	0.093
rs2073869	C	0.83	0.019	0.003	0.088	0.100
rs1243182	C	0.69	-0.019	0.002	0.170	0.078
rs2045147	A	0.45	0.013	0.002	-0.230	0.078
rs11201422	T	0.67	0.013	0.002	0.110	0.094
rs7089973	C	0.62	-0.013	0.002	-0.036	0.083
rs11245482	T	0.61	-0.013	0.002	0.033	0.083
rs17727474	C	0.83	0.018	0.003	0.033	0.099
rs12289262	C	0.73	-0.014	0.002	0.024	0.089
rs801733	A	0.64	0.017	0.002	0.094	0.076
rs17207890	G	0.66	0.016	0.002	-0.057	0.083
rs11218575	C	0.57	0.015	0.002	0.074	0.080
rs4937842	G	0.63	-0.013	0.002	0.200	0.077
rs10876864	G	0.43	-0.013	0.002	0.140	0.074
rs8756	C	0.48	-0.013	0.002	-0.060	0.078
rs9563168	G	0.79	0.018	0.003	0.002	0.089
rs9528023	C	0.72	0.013	0.002	0.120	0.091
rs1022785	G	0.14	0.018	0.003	-0.023	0.100
rs10145592	C	0.41	-0.015	0.002	0.055	0.079
rs8043253	C	0.57	-0.012	0.002	0.056	0.078
rs2460	G	0.74	-0.015	0.002	-0.019	0.095
rs7189927	T	0.36	0.015	0.002	0.051	0.077
rs749671	G	0.63	0.016	0.002	-0.045	0.076
rs2447098	C	0.48	-0.015	0.002	0.039	0.080
rs11654952	T	0.86	-0.017	0.003	0.120	0.110
rs2584597	T	0.66	0.015	0.002	-0.045	0.083

rs9902312	T	0.68	0.015	0.002	-0.067	0.080
rs303753	G	0.65	-0.014	0.002	0.043	0.081
rs11081851	G	0.44	-0.013	0.002	0.001	0.078
rs9964724	C	0.32	0.018	0.002	-0.029	0.081
rs1156541	C	0.22	0.014	0.003	0.096	0.089
rs6124476	T	0.59	0.012	0.002	-0.007	0.078
rs6031440	A	0.40	0.012	0.002	0.130	0.080
rs7609	A	0.23	0.015	0.003	-0.018	0.095
**BMI**						
rs10041724	T	0.81	0.018	0.003	0.006	0.002
rs10054327	G	0.58	0.017	0.002	0.003	0.002
rs10145592	C	0.41	-0.015	0.002	-0.006	0.002
rs10189857	A	0.57	-0.020	0.002	-0.007	0.002
rs1022785	G	0.14	0.018	0.003	0.008	0.002
rs10234444	G	0.82	0.016	0.003	0.005	0.002
rs10246289	A	0.11	0.019	0.003	0.005	0.003
rs1031423	T	0.22	-0.019	0.003	0.004	0.002
rs10427502	G	0.62	0.014	0.002	0.012	0.002
rs10737620	T	0.27	0.014	0.002	0.010	0.002
rs10771746	C	0.72	-0.014	0.002	-0.002	0.002
rs10772643	C	0.11	0.025	0.003	0.010	0.003
rs10876864	G	0.43	-0.013	0.002	-0.011	0.002
rs10890123	C	0.77	0.014	0.003	0.002	0.002
rs10932837	C	0.49	-0.013	0.002	0.002	0.002
rs10940659	A	0.53	-0.013	0.002	-0.007	0.002
rs10994943	T	0.58	0.013	0.002	0.003	0.002
rs11130793	C	0.60	0.013	0.002	0.008	0.002
rs11201422	T	0.67	0.013	0.002	0.008	0.002
rs11218575	C	0.57	0.015	0.002	0.003	0.002
rs11245482	T	0.61	-0.013	0.002	-0.010	0.002
rs114328297	T	0.78	0.014	0.003	-0.001	0.002
rs114600294	G	0.79	-0.016	0.003	-0.006	0.002
rs1156541	C	0.22	0.014	0.003	0.011	0.002
rs11654952	T	0.86	-0.017	0.003	-0.004	0.003
rs11657730	C	0.64	0.013	0.002	0.003	0.002
rs11689199	A	0.60	0.018	0.002	0.017	0.002
rs11714337	G	0.57	0.014	0.002	0.007	0.002
rs11763734	A	0.51	-0.012	0.002	-0.003	0.002
rs11810109	A	0.70	0.016	0.002	0.001	0.002
rs12105701	C	0.40	-0.013	0.002	-0.010	0.002
rs12272012	G	0.96	-0.030	0.005	-0.009	0.005
rs12289262	C	0.73	-0.014	0.002	-0.007	0.002
rs1243182	C	0.69	-0.019	0.002	-0.017	0.002
rs12491503	G	0.67	-0.014	0.002	-0.004	0.002
rs12541615	T	0.82	-0.018	0.003	-0.015	0.003
rs12554512	T	0.58	0.021	0.002	-0.010	0.002
rs12725114	G	0.80	0.015	0.003	0.012	0.002

rs1278847	C	0.69	0.016	0.002	-0.011	0.002
rs13029509	G	0.52	-0.018	0.002	-0.007	0.002
rs13107325	C	0.93	-0.029	0.004	-0.047	0.003
rs138256022	C	0.96	-0.031	0.006	0.002	0.005
rs1421334	A	0.45	0.017	0.002	0.014	0.002
rs1451533	G	0.72	-0.016	0.002	-0.016	0.002
rs17207890	G	0.66	0.016	0.002	0.001	0.002
rs17379561	A	0.86	-0.025	0.003	-0.018	0.002
rs17512836	T	0.97	0.042	0.007	0.016	0.006
rs17727474	C	0.83	0.018	0.003	0.000	0.002
rs17789218	T	0.76	0.019	0.003	-0.011	0.002
rs2034768	A	0.49	0.015	0.002	0.020	0.002
rs2045147	A	0.45	0.013	0.002	0.005	0.002
rs2073869	C	0.83	0.019	0.003	0.007	0.002
rs2164744	T	0.64	-0.013	0.002	-0.005	0.002
rs2173650	G	0.85	0.018	0.003	0.004	0.003
rs2447098	C	0.48	-0.015	0.002	-0.001	0.002
rs2460	G	0.74	-0.015	0.002	-0.011	0.002
rs2584597	T	0.66	0.015	0.002	0.013	0.004
rs2616830	G	0.46	0.016	0.002	0.001	0.002
rs262890	A	0.70	-0.019	0.002	-0.014	0.002
rs2717559	A	0.56	0.012	0.002	0.004	0.002
rs2787374	T	0.41	0.012	0.002	0.011	0.002
rs303753	G	0.65	-0.014	0.002	-0.020	0.002
rs34864022	A	0.93	-0.026	0.004	0.000	0.004
rs35574015	T	0.71	-0.013	0.002	0.008	0.002
rs374722	G	0.15	0.024	0.003	0.014	0.002
rs3754970	T	0.50	-0.015	0.002	-0.005	0.002
rs3796386	G	0.57	-0.026	0.002	-0.018	0.002
rs42210	G	0.29	-0.014	0.002	-0.003	0.002
rs4334769	G	0.47	0.012	0.002	0.002	0.002
rs4382592	T	0.30	0.014	0.002	0.009	0.002
rs4523073	A	0.59	-0.014	0.002	-0.003	0.002
rs4577309	A	0.47	0.016	0.002	0.002	0.002
rs4675246	G	0.80	-0.015	0.003	0.002	0.002
rs4775373	T	0.36	0.013	0.002	0.005	0.002
rs4845364	A	0.50	-0.015	0.002	-0.001	0.002
rs4937842	G	0.63	-0.013	0.002	-0.008	0.002
rs4973576	C	0.30	-0.015	0.002	0.002	0.002
rs55700114	G	0.71	-0.014	0.002	-0.005	0.002
rs56103247	C	0.94	0.030	0.005	0.023	0.004
rs56858768	G	0.70	-0.015	0.002	-0.017	0.002
rs57585211	T	0.83	-0.017	0.003	-0.005	0.003
rs6131281	C	0.60	0.016	0.002	0.007	0.002
rs6141814	C	0.61	-0.014	0.002	0.002	0.002
rs62379379	G	0.93	-0.026	0.004	-0.005	0.004
rs62641636	A	0.69	0.014	0.002	0.006	0.002

rs6472942	T	0.57	-0.013	0.002	0.017	0.002
rs6673341	T	0.47	-0.015	0.002	-0.008	0.002
rs6797840	A	0.46	-0.016	0.002	-0.013	0.002
rs6825241	C	0.54	-0.017	0.002	-0.004	0.002
rs6850494	A	0.62	-0.014	0.002	0.001	0.002
rs6905544	A	0.40	-0.019	0.002	-0.017	0.002
rs6973656	A	0.60	-0.013	0.002	-0.010	0.002
rs6996198	C	0.84	-0.016	0.003	-0.007	0.002
rs7089973	C	0.62	-0.013	0.002	0.000	0.002
rs7157001	A	0.74	-0.014	0.002	-0.007	0.002
rs71658797	T	0.88	-0.020	0.003	-0.037	0.003
rs7184800	G	0.70	0.017	0.002	-0.006	0.002
rs7189927	T	0.36	0.015	0.002	0.026	0.002
rs7248205	C	0.40	0.014	0.002	0.008	0.002
rs72671494	T	0.86	-0.017	0.003	-0.010	0.003
rs72781699	G	0.80	-0.019	0.003	0.000	0.002
rs72828890	C	0.87	0.019	0.003	0.011	0.003
rs72834698	G	0.86	0.023	0.003	0.015	0.003
rs749671	G	0.63	0.016	0.002	0.021	0.002
rs7564130	T	0.64	-0.015	0.002	-0.002	0.002
rs7693082	G	0.30	0.015	0.002	0.008	0.002
rs7693703	G	0.91	0.023	0.004	0.005	0.003
rs7700107	A	0.86	-0.021	0.003	-0.010	0.003
rs77215114	A	0.93	0.024	0.004	0.013	0.004
rs801733	A	0.64	0.017	0.002	0.011	0.002
rs8043253	C	0.57	-0.012	0.002	-0.005	0.002
rs8756	C	0.48	-0.013	0.002	0.005	0.002
rs9471333	C	0.45	0.013	0.002	0.021	0.002
rs9563168	G	0.79	0.018	0.003	0.008	0.002
rs9718104	T	0.94	-0.041	0.005	0.000	0.004
rs973734	C	0.15	0.017	0.003	0.004	0.002
rs9834970	T	0.50	0.013	0.002	0.007	0.002
rs984409	G	0.36	-0.015	0.002	-0.005	0.002
rs9867121	C	0.82	0.019	0.003	0.008	0.002
rs9902312	T	0.68	0.015	0.002	0.001	0.002
rs9964724	C	0.32	0.018	0.002	0.000	0.002
**WHR**						
rs10041724	T	0.81	0.018	0.003	0.007	0.002
rs10054327	G	0.58	0.017	0.002	0.007	0.002
rs10145592	C	0.41	-0.015	0.002	-0.005	0.002
rs10189857	A	0.57	-0.020	0.002	-0.005	0.002
rs1022785	G	0.14	0.018	0.003	0.010	0.003
rs10234444	G	0.82	0.016	0.003	0.006	0.002
rs10246289	A	0.11	0.019	0.003	0.006	0.003
rs1031423	T	0.22	-0.019	0.003	0.004	0.002
rs10427502	G	0.62	0.014	0.002	0.007	0.002
rs10737620	T	0.27	0.014	0.002	0.006	0.002

rs10771746	C	0.72	-0.014	0.002	0.009	0.002
rs10772643	C	0.11	0.025	0.003	0.008	0.003
rs10876864	G	0.43	-0.013	0.002	-0.007	0.002
rs10890123	C	0.77	0.014	0.003	0.002	0.002
rs10932837	C	0.49	-0.013	0.002	-0.001	0.002
rs10940659	A	0.53	-0.013	0.002	-0.009	0.002
rs10994943	T	0.58	0.013	0.002	0.002	0.002
rs11130793	C	0.60	0.013	0.002	0.004	0.002
rs11201422	T	0.67	0.013	0.002	0.000	0.002
rs11218575	C	0.57	0.015	0.002	0.001	0.002
rs11245482	T	0.61	-0.013	0.002	-0.002	0.002
rs114328297	T	0.78	0.014	0.003	-0.001	0.002
rs114600294	G	0.79	-0.016	0.003	-0.001	0.002
rs1156541	C	0.22	0.014	0.003	0.010	0.002
rs11654952	T	0.86	-0.017	0.003	-0.006	0.003
rs11657730	C	0.64	0.013	0.002	0.004	0.002
rs11689199	A	0.60	0.018	0.002	0.012	0.002
rs11714337	G	0.57	0.014	0.002	0.005	0.002
rs11763734	A	0.51	-0.012	0.002	-0.006	0.002
rs11810109	A	0.70	0.016	0.002	0.007	0.002
rs12105701	C	0.40	-0.013	0.002	-0.003	0.002
rs12272012	G	0.96	-0.030	0.005	-0.009	0.005
rs12289262	C	0.73	-0.014	0.002	-0.004	0.002
rs1243182	C	0.69	-0.019	0.002	-0.013	0.002
rs12491503	G	0.67	-0.014	0.002	-0.004	0.002
rs12541615	T	0.82	-0.018	0.003	-0.007	0.003
rs12554512	T	0.58	0.021	0.002	-0.004	0.002
rs12725114	G	0.80	0.015	0.003	0.010	0.002
rs1278847	C	0.69	0.016	0.002	-0.008	0.002
rs13029509	G	0.52	-0.018	0.002	-0.006	0.002
rs13107325	C	0.93	-0.029	0.004	0.003	0.003
rs138256022	C	0.96	-0.031	0.006	-0.005	0.005
rs1421334	A	0.45	0.017	0.002	0.011	0.002
rs1451533	G	0.72	-0.016	0.002	-0.006	0.002
rs17207890	G	0.66	0.016	0.002	0.004	0.002
rs17379561	A	0.86	-0.025	0.003	-0.018	0.003
rs17512836	T	0.97	0.042	0.007	0.019	0.006
rs17727474	C	0.83	0.018	0.003	0.004	0.002
rs17789218	T	0.76	0.019	0.003	0.007	0.002
rs2034768	A	0.49	0.015	0.002	0.012	0.002
rs2045147	A	0.45	0.013	0.002	0.005	0.002
rs2073869	C	0.83	0.019	0.003	0.007	0.002
rs2164744	T	0.64	-0.013	0.002	0.000	0.002
rs2173650	G	0.85	0.018	0.003	0.005	0.003
rs2447098	C	0.48	-0.015	0.002	0.001	0.002
rs2460	G	0.74	-0.015	0.002	-0.011	0.002
rs2584597	T	0.66	0.015	0.002	0.007	0.004

rs2616830	G	0.46	0.016	0.002	0.000	0.002
rs262890	A	0.70	-0.019	0.002	-0.008	0.002
rs2717559	A	0.56	0.012	0.002	0.001	0.002
rs2787374	T	0.41	0.012	0.002	0.000	0.002
rs303753	G	0.65	-0.014	0.002	-0.010	0.002
rs34864022	A	0.93	-0.026	0.004	-0.004	0.004
rs35574015	T	0.71	-0.013	0.002	0.002	0.002
rs374722	G	0.15	0.024	0.003	0.006	0.003
rs3754970	T	0.50	-0.015	0.002	-0.002	0.002
rs3796386	G	0.57	-0.026	0.002	-0.011	0.002
rs42210	G	0.29	-0.014	0.002	-0.004	0.002
rs4334769	G	0.47	0.012	0.002	0.000	0.002
rs4382592	T	0.30	0.014	0.002	0.012	0.002
rs4523073	A	0.59	-0.014	0.002	-0.007	0.002
rs4577309	A	0.47	0.016	0.002	0.003	0.002
rs4675246	G	0.80	-0.015	0.003	0.001	0.002
rs4775373	T	0.36	0.013	0.002	0.005	0.002
rs4845364	A	0.50	-0.015	0.002	0.002	0.002
rs4937842	G	0.63	-0.013	0.002	-0.004	0.002
rs4973576	C	0.30	-0.015	0.002	0.001	0.002
rs55700114	G	0.71	-0.014	0.002	-0.001	0.002
rs56103247	C	0.94	0.030	0.005	0.002	0.005
rs56858768	G	0.70	-0.015	0.002	-0.010	0.002
rs57585211	T	0.83	-0.017	0.003	-0.002	0.003
rs6131281	C	0.60	0.016	0.002	0.007	0.002
rs6141814	C	0.61	-0.014	0.002	0.001	0.002
rs62379379	G	0.93	-0.026	0.004	-0.004	0.004
rs62641636	A	0.69	0.014	0.002	0.009	0.002
rs6472942	T	0.57	-0.013	0.002	0.003	0.002
rs6673341	T	0.47	-0.015	0.002	-0.004	0.002
rs6797840	A	0.46	-0.016	0.002	-0.007	0.002
rs6825241	C	0.54	-0.017	0.002	-0.007	0.002
rs6850494	A	0.62	-0.014	0.002	-0.002	0.002
rs6905544	A	0.40	-0.019	0.002	-0.012	0.002
rs6973656	A	0.60	-0.013	0.002	0.010	0.002
rs6996198	C	0.84	-0.016	0.003	0.000	0.002
rs7089973	C	0.62	-0.013	0.002	-0.002	0.002
rs7157001	A	0.74	-0.014	0.002	-0.006	0.002
rs71658797	T	0.88	-0.020	0.003	-0.010	0.003
rs7184800	G	0.70	0.017	0.002	0.009	0.002
rs7189927	T	0.36	0.015	0.002	0.017	0.002
rs7248205	C	0.40	0.014	0.002	-0.003	0.002
rs72671494	T	0.86	-0.017	0.003	-0.009	0.003
rs72781699	G	0.80	-0.019	0.003	-0.008	0.002
rs72828890	C	0.87	0.019	0.003	0.006	0.003
rs72834698	G	0.86	0.023	0.003	0.004	0.003
rs749671	G	0.63	0.016	0.002	0.007	0.002

rs7564130	T	0.64	-0.015	0.002	0.001	0.002
rs7693082	G	0.30	0.015	0.002	0.000	0.002
rs7693703	G	0.91	0.023	0.004	0.001	0.003
rs7700107	A	0.86	-0.021	0.003	-0.012	0.003
rs77215114	A	0.93	0.024	0.004	0.004	0.004
rs801733	A	0.64	0.017	0.002	0.006	0.002
rs8043253	C	0.57	-0.012	0.002	0.003	0.002
rs8756	C	0.48	-0.013	0.002	-0.001	0.002
rs9471333	C	0.45	0.013	0.002	0.014	0.002
rs9563168	G	0.79	0.018	0.003	0.003	0.002
rs9718104	T	0.94	-0.041	0.005	-0.003	0.004
rs973734	C	0.15	0.017	0.003	-0.002	0.003
rs9834970	T	0.50	0.013	0.002	0.003	0.002
rs984409	G	0.36	-0.015	0.002	-0.010	0.002
rs9867121	C	0.82	0.019	0.003	0.004	0.003
rs9902312	T	0.68	0.015	0.002	-0.005	0.002
rs9964724	C	0.32	0.018	0.002	0.006	0.002
**Smoking**						
rs10041724	T	0.81	0.018	0.003	-0.002	0.005
rs10054327	G	0.58	0.017	0.002	0.001	0.004
rs10145592	C	0.41	-0.015	0.002	-0.001	0.004
rs10189857	A	0.57	-0.020	0.002	0.009	0.004
rs1022785	G	0.14	0.018	0.003	-0.002	0.005
rs10234444	G	0.82	0.016	0.003	0.002	0.005
rs10246289	A	0.11	0.019	0.003	0.001	0.006
rs1031423	T	0.22	-0.019	0.003	0.002	0.005
rs10427502	G	0.62	0.014	0.002	0.010	0.004
rs10737620	T	0.27	0.014	0.002	-0.004	0.004
rs10771746	C	0.72	-0.014	0.002	0.000	0.004
rs10772643	C	0.11	0.025	0.003	-0.002	0.006
rs10876864	G	0.43	-0.013	0.002	-0.001	0.004
rs10890123	C	0.77	0.014	0.003	0.000	0.005
rs10932837	C	0.49	-0.013	0.002	-0.002	0.004
rs10940659	A	0.53	-0.013	0.002	-0.008	0.004
rs10994943	T	0.58	0.013	0.002	0.005	0.004
rs11130793	C	0.60	0.013	0.002	-0.002	0.004
rs11201422	T	0.67	0.013	0.002	0.008	0.004
rs11218575	C	0.57	0.015	0.002	-0.001	0.004
rs11245482	T	0.61	-0.013	0.002	0.003	0.004
rs114328297	T	0.78	0.014	0.003	-0.014	0.005
rs114600294	G	0.79	-0.016	0.003	-0.008	0.005
rs1156541	C	0.22	0.014	0.003	-0.008	0.004
rs11654952	T	0.86	-0.017	0.003	-0.005	0.007
rs11657730	C	0.64	0.013	0.002	0.000	0.005
rs11689199	A	0.60	0.018	0.002	-0.006	0.004
rs11714337	G	0.57	0.014	0.002	-0.007	0.004
rs11763734	A	0.51	-0.012	0.002	-0.007	0.004

rs11810109	A	0.70	0.016	0.002	-0.008	0.004
rs12105701	C	0.40	-0.013	0.002	-0.002	0.004
rs12272012	G	0.96	-0.030	0.005	0.010	0.009
rs12289262	C	0.73	-0.014	0.002	0.003	0.004
rs1243182	C	0.69	-0.019	0.002	-0.004	0.004
rs12491503	G	0.67	-0.014	0.002	0.008	0.004
rs12541615	T	0.82	-0.018	0.003	-0.006	0.005
rs12554512	T	0.58	0.021	0.002	0.000	0.004
rs12725114	G	0.80	0.015	0.003	0.010	0.005
rs1278847	C	0.69	0.016	0.002	0.002	0.004
rs13029509	G	0.52	-0.018	0.002	0.006	0.004
rs13107325	C	0.93	-0.029	0.004	0.004	0.009
rs138256022	C	0.96	-0.031	0.006	0.006	0.009
rs1421334	A	0.45	0.017	0.002	0.000	0.004
rs1451533	G	0.72	-0.016	0.002	0.007	0.004
rs17207890	G	0.66	0.016	0.002	-0.006	0.005
rs17379561	A	0.86	-0.025	0.003	0.006	0.005
rs17512836	T	0.97	0.042	0.007	-0.004	0.013
rs17727474	C	0.83	0.018	0.003	0.000	0.005
rs17789218	T	0.76	0.019	0.003	0.009	0.005
rs2034768	A	0.49	0.015	0.002	-0.002	0.004
rs2045147	A	0.45	0.013	0.002	-0.011	0.004
rs2073869	C	0.83	0.019	0.003	-0.003	0.005
rs2164744	T	0.64	-0.013	0.002	0.008	0.004
rs2173650	G	0.85	0.018	0.003	0.004	0.005
rs2447098	C	0.48	-0.015	0.002	0.011	0.004
rs2460	G	0.74	-0.015	0.002	0.008	0.004
rs2616830	G	0.46	0.016	0.002	-0.003	0.004
rs262890	A	0.70	-0.019	0.002	0.003	0.004
rs2717559	A	0.56	0.012	0.002	-0.002	0.004
rs2787374	T	0.41	0.012	0.002	-0.005	0.004
rs303753	G	0.65	-0.014	0.002	0.007	0.004
rs34864022	A	0.93	-0.026	0.004	0.016	0.007
rs35574015	T	0.71	-0.013	0.002	-0.007	0.004
rs374722	G	0.15	0.024	0.003	-0.009	0.005
rs3754970	T	0.50	-0.015	0.002	0.002	0.004
rs3796386	G	0.57	-0.026	0.002	0.001	0.004
rs42210	G	0.29	-0.014	0.002	0.004	0.004
rs4334769	G	0.47	0.012	0.002	-0.005	0.004
rs4382592	T	0.30	0.014	0.002	-0.005	0.004
rs4523073	A	0.59	-0.014	0.002	0.011	0.004
rs4577309	A	0.47	0.016	0.002	-0.005	0.004
rs4675246	G	0.80	-0.015	0.003	0.000	0.005
rs4775373	T	0.36	0.013	0.002	-0.006	0.004
rs4845364	A	0.50	-0.015	0.002	0.006	0.004
rs4937842	G	0.63	-0.013	0.002	0.006	0.004
rs4973576	C	0.30	-0.015	0.002	0.003	0.005

rs55700114	G	0.71	-0.014	0.002	0.008	0.004
rs56103247	C	0.94	0.030	0.005	-0.013	0.009
rs56858768	G	0.70	-0.015	0.002	0.000	0.004
rs57585211	T	0.83	-0.017	0.003	-0.003	0.005
rs6131281	C	0.60	0.016	0.002	0.013	0.004
rs6141814	C	0.61	-0.014	0.002	0.009	0.004
rs62379379	G	0.93	-0.026	0.004	0.007	0.007
rs62641636	A	0.69	0.014	0.002	-0.012	0.005
rs6472942	T	0.57	-0.013	0.002	0.004	0.004
rs6673341	T	0.47	-0.015	0.002	-0.001	0.004
rs6797840	A	0.46	-0.016	0.002	-0.001	0.004
rs6825241	C	0.54	-0.017	0.002	-0.004	0.004
rs6850494	A	0.62	-0.014	0.002	0.007	0.004
rs6905544	A	0.40	-0.019	0.002	0.001	0.004
rs6973656	A	0.60	-0.013	0.002	-0.002	0.004
rs6996198	C	0.84	-0.016	0.003	-0.003	0.005
rs7089973	C	0.62	-0.013	0.002	0.003	0.004
rs7157001	A	0.74	-0.014	0.002	-0.008	0.004
rs71658797	T	0.88	-0.020	0.003	0.011	0.006
rs7184800	G	0.70	0.017	0.002	-0.004	0.004
rs7189927	T	0.36	0.015	0.002	-0.001	0.004
rs7248205	C	0.40	0.014	0.002	0.008	0.004
rs72671494	T	0.86	-0.017	0.003	0.013	0.005
rs72781699	G	0.80	-0.019	0.003	0.006	0.005
rs72828890	C	0.87	0.019	0.003	-0.002	0.006
rs72834698	G	0.86	0.023	0.003	-0.007	0.005
rs749671	G	0.63	0.016	0.002	-0.013	0.004
rs7564130	T	0.64	-0.015	0.002	0.002	0.004
rs7693082	G	0.30	0.015	0.002	-0.003	0.004
rs7693703	G	0.91	0.023	0.004	-0.011	0.006
rs7700107	A	0.86	-0.021	0.003	0.000	0.006
rs77215114	A	0.93	0.024	0.004	0.003	0.007
rs801733	A	0.64	0.017	0.002	-0.003	0.004
rs8043253	C	0.57	-0.012	0.002	-0.007	0.004
rs8756	C	0.48	-0.013	0.002	0.004	0.004
rs9471333	C	0.45	0.013	0.002	-0.001	0.004
rs9563168	G	0.79	0.018	0.003	0.006	0.005
rs9718104	T	0.94	-0.041	0.005	-0.005	0.008
rs973734	C	0.15	0.017	0.003	-0.012	0.005
rs9834970	T	0.50	0.013	0.002	-0.007	0.004
rs984409	G	0.36	-0.015	0.002	0.004	0.004
rs9867121	C	0.82	0.019	0.003	0.007	0.005
rs9902312	T	0.68	0.015	0.002	0.004	0.004
rs9964724	C	0.32	0.018	0.002	-0.009	0.004
**Drinking**						
rs10041724	T	0.81	0.018	0.003	0.004	0.004
rs10054327	G	0.58	0.017	0.002	0.002	0.003

rs10145592	C	0.41	-0.015	0.002	-0.010	0.003
rs10189857	A	0.57	-0.020	0.002	-0.007	0.003
rs1022785	G	0.14	0.018	0.003	-0.005	0.004
rs10234444	G	0.82	0.016	0.003	0.000	0.004
rs10246289	A	0.11	0.019	0.003	0.007	0.005
rs1031423	T	0.22	-0.019	0.003	-0.001	0.004
rs10427502	G	0.62	0.014	0.002	0.000	0.003
rs10737620	T	0.27	0.014	0.002	-0.001	0.003
rs10771746	C	0.72	-0.014	0.002	0.004	0.003
rs10772643	C	0.11	0.025	0.003	0.002	0.005
rs10876864	G	0.43	-0.013	0.002	-0.004	0.003
rs10890123	C	0.77	0.014	0.003	0.003	0.004
rs10932837	C	0.49	-0.013	0.002	-0.001	0.003
rs10940659	A	0.53	-0.013	0.002	-0.004	0.003
rs10994943	T	0.58	0.013	0.002	0.000	0.003
rs11130793	C	0.60	0.013	0.002	0.003	0.003
rs11201422	T	0.67	0.013	0.002	0.005	0.003
rs11218575	C	0.57	0.015	0.002	0.000	0.003
rs11245482	T	0.61	-0.013	0.002	-0.001	0.003
rs114328297	T	0.78	0.014	0.003	-0.007	0.004
rs114600294	G	0.79	-0.016	0.003	-0.005	0.004
rs1156541	C	0.22	0.014	0.003	-0.001	0.004
rs11654952	T	0.86	-0.017	0.003	0.000	0.005
rs11657730	C	0.64	0.013	0.002	0.002	0.003
rs11689199	A	0.60	0.018	0.002	0.000	0.003
rs11714337	G	0.57	0.014	0.002	0.003	0.003
rs11763734	A	0.51	-0.012	0.002	0.006	0.003
rs11810109	A	0.70	0.016	0.002	-0.003	0.003
rs12105701	C	0.40	-0.013	0.002	-0.002	0.003
rs12272012	G	0.96	-0.030	0.005	0.000	0.007
rs12289262	C	0.73	-0.014	0.002	0.000	0.003
rs1243182	C	0.69	-0.019	0.002	-0.003	0.003
rs12491503	G	0.67	-0.014	0.002	0.000	0.003
rs12541615	T	0.82	-0.018	0.003	0.001	0.004
rs12554512	T	0.58	0.021	0.002	0.001	0.003
rs12725114	G	0.80	0.015	0.003	-0.003	0.004
rs1278847	C	0.69	0.016	0.002	0.001	0.003
rs13029509	G	0.52	-0.018	0.002	0.005	0.003
rs13107325	C	0.93	-0.029	0.004	-0.035	0.007
rs138256022	C	0.96	-0.031	0.006	0.005	0.007
rs1421334	A	0.45	0.017	0.002	0.006	0.003
rs1451533	G	0.72	-0.016	0.002	-0.001	0.003
rs17207890	G	0.66	0.016	0.002	-0.008	0.003
rs17379561	A	0.86	-0.025	0.003	0.003	0.004
rs17512836	T	0.97	0.042	0.007	0.009	0.009
rs17727474	C	0.83	0.018	0.003	-0.002	0.004
rs17789218	T	0.76	0.019	0.003	0.003	0.004

rs2034768	A	0.49	0.015	0.002	-0.004	0.003
rs2045147	A	0.45	0.013	0.002	-0.002	0.003
rs2073869	C	0.83	0.019	0.003	0.003	0.004
rs2164744	T	0.64	-0.013	0.002	0.006	0.003
rs2173650	G	0.85	0.018	0.003	0.001	0.004
rs2447098	C	0.48	-0.015	0.002	0.001	0.003
rs2460	G	0.74	-0.015	0.002	-0.002	0.003
rs2616830	G	0.46	0.016	0.002	0.004	0.003
rs262890	A	0.70	-0.019	0.002	0.002	0.003
rs2717559	A	0.56	0.012	0.002	0.003	0.003
rs2787374	T	0.41	0.012	0.002	0.001	0.003
rs303753	G	0.65	-0.014	0.002	-0.004	0.003
rs34864022	A	0.93	-0.026	0.004	-0.013	0.006
rs35574015	T	0.71	-0.013	0.002	0.004	0.003
rs374722	G	0.15	0.024	0.003	0.009	0.004
rs3754970	T	0.50	-0.015	0.002	0.008	0.003
rs3796386	G	0.57	-0.026	0.002	-0.002	0.003
rs42210	G	0.29	-0.014	0.002	-0.001	0.003
rs4334769	G	0.47	0.012	0.002	0.006	0.003
rs4382592	T	0.30	0.014	0.002	0.004	0.003
rs4523073	A	0.59	-0.014	0.002	0.005	0.003
rs4577309	A	0.47	0.016	0.002	0.005	0.003
rs4675246	G	0.80	-0.015	0.003	0.006	0.004
rs4775373	T	0.36	0.013	0.002	-0.001	0.003
rs4845364	A	0.50	-0.015	0.002	0.004	0.003
rs4937842	G	0.63	-0.013	0.002	-0.001	0.003
rs4973576	C	0.30	-0.015	0.002	0.002	0.004
rs55700114	G	0.71	-0.014	0.002	0.001	0.003
rs56103247	C	0.94	0.030	0.005	0.006	0.007
rs56858768	G	0.70	-0.015	0.002	-0.002	0.003
rs57585211	T	0.83	-0.017	0.003	-0.007	0.004
rs6131281	C	0.60	0.016	0.002	-0.004	0.003
rs6141814	C	0.61	-0.014	0.002	0.005	0.003
rs62379379	G	0.93	-0.026	0.004	-0.003	0.006
rs62641636	A	0.69	0.014	0.002	-0.001	0.003
rs6472942	T	0.57	-0.013	0.002	-0.001	0.003
rs6673341	T	0.47	-0.015	0.002	0.004	0.003
rs6797840	A	0.46	-0.016	0.002	0.004	0.003
rs6825241	C	0.54	-0.017	0.002	-0.005	0.003
rs6850494	A	0.62	-0.014	0.002	0.002	0.003
rs6905544	A	0.40	-0.019	0.002	-0.009	0.003
rs6973656	A	0.60	-0.013	0.002	0.001	0.003
rs6996198	C	0.84	-0.016	0.003	-0.007	0.004
rs7089973	C	0.62	-0.013	0.002	-0.008	0.003
rs7157001	A	0.74	-0.014	0.002	0.001	0.003
rs71658797	T	0.88	-0.020	0.003	0.003	0.005
rs7184800	G	0.70	0.017	0.002	0.004	0.003

rs7189927	T	0.36	0.015	0.002	0.015	0.003
rs7248205	C	0.40	0.014	0.002	-0.003	0.003
rs72671494	T	0.86	-0.017	0.003	0.006	0.004
rs72781699	G	0.80	-0.019	0.003	-0.010	0.004
rs72828890	C	0.87	0.019	0.003	-0.007	0.005
rs72834698	G	0.86	0.023	0.003	0.001	0.004
rs749671	G	0.63	0.016	0.002	0.007	0.003
rs7564130	T	0.64	-0.015	0.002	0.004	0.003
rs7693082	G	0.30	0.015	0.002	-0.002	0.003
rs7693703	G	0.91	0.023	0.004	0.008	0.005
rs7700107	A	0.86	-0.021	0.003	0.007	0.005
rs77215114	A	0.93	0.024	0.004	0.002	0.005
rs801733	A	0.64	0.017	0.002	0.004	0.003
rs8043253	C	0.57	-0.012	0.002	-0.006	0.003
rs8756	C	0.48	-0.013	0.002	-0.001	0.003
rs9471333	C	0.45	0.013	0.002	-0.001	0.003
rs9563168	G	0.79	0.018	0.003	0.007	0.004
rs9718104	T	0.94	-0.041	0.005	-0.005	0.006
rs973734	C	0.15	0.017	0.003	-0.005	0.004
rs9834970	T	0.50	0.013	0.002	-0.001	0.003
rs984409	G	0.36	-0.015	0.002	0.000	0.003
rs9867121	C	0.82	0.019	0.003	-0.004	0.004
rs9902312	T	0.68	0.015	0.002	-0.002	0.003
rs9964724	C	0.32	0.018	0.002	-0.002	0.003
**Physical activity**						
rs10041724	T	0.81	0.018	0.003	0.004	0.005
rs10054327	G	0.58	0.017	0.002	-0.010	0.004
rs10145592	C	0.41	-0.015	0.002	0.006	0.004
rs10189857	A	0.57	-0.020	0.002	-0.001	0.004
rs1022785	G	0.14	0.018	0.003	-0.001	0.006
rs10234444	G	0.82	0.016	0.003	-0.004	0.006
rs10246289	A	0.11	0.019	0.003	0.000	0.007
rs1031423	T	0.22	-0.019	0.003	-0.012	0.005
rs10427502	G	0.62	0.014	0.002	-0.009	0.004
rs10737620	T	0.27	0.014	0.002	0.000	0.005
rs10771746	C	0.72	-0.014	0.002	0.005	0.005
rs10772643	C	0.11	0.025	0.003	-0.012	0.007
rs10876864	G	0.43	-0.013	0.002	-0.016	0.004
rs10890123	C	0.77	0.014	0.003	-0.006	0.005
rs10932837	C	0.49	-0.013	0.002	-0.008	0.004
rs10940659	A	0.53	-0.013	0.002	0.003	0.004
rs10994943	T	0.58	0.013	0.002	-0.005	0.004
rs11020045	A	0.67	-0.013	0.002	0.006	0.005
rs11130793	C	0.60	0.013	0.002	-0.004	0.004
rs11201422	T	0.67	0.013	0.002	-0.003	0.005
rs11218575	C	0.57	0.015	0.002	-0.006	0.004
rs11245482	T	0.61	-0.013	0.002	0.006	0.004

rs114328297	T	0.78	0.014	0.003	-0.001	0.005
rs114600294	G	0.79	-0.016	0.003	0.007	0.005
rs1156541	C	0.22	0.014	0.003	0.009	0.005
rs11654952	T	0.86	-0.017	0.003	0.009	0.006
rs11657730	C	0.64	0.013	0.002	-0.008	0.004
rs11689199	A	0.60	0.018	0.002	-0.010	0.004
rs11714337	G	0.57	0.014	0.002	0.006	0.004
rs11763734	A	0.51	-0.012	0.002	0.003	0.004
rs11810109	A	0.70	0.016	0.002	0.005	0.005
rs12105701	C	0.40	-0.013	0.002	0.001	0.004
rs12272012	G	0.96	-0.030	0.005	-0.012	0.010
rs12289262	C	0.73	-0.014	0.002	-0.010	0.005
rs1243182	C	0.69	-0.019	0.002	0.018	0.005
rs12476388	C	0.71	0.013	0.002	-0.005	0.005
rs12491503	G	0.67	-0.014	0.002	-0.001	0.005
rs12541615	T	0.82	-0.018	0.003	0.008	0.006
rs12554512	T	0.58	0.021	0.002	0.002	0.004
rs12725114	G	0.80	0.015	0.003	0.002	0.005
rs1278847	C	0.69	0.016	0.002	0.004	0.005
rs13029509	G	0.52	-0.018	0.002	0.005	0.004
rs13107325	C	0.93	-0.029	0.004	-0.010	0.008
rs138256022	C	0.96	-0.031	0.006	0.004	0.011
rs141184308	A	0.98	0.043	0.008	-0.023	0.015
rs1421334	A	0.45	0.017	0.002	-0.010	0.004
rs1451533	G	0.72	-0.016	0.002	0.008	0.005
rs17207890	G	0.66	0.016	0.002	0.001	0.005
rs17379561	A	0.86	-0.025	0.003	-0.005	0.006
rs17512836	T	0.97	0.042	0.007	-0.018	0.013
rs17727474	C	0.83	0.018	0.003	0.000	0.006
rs17789218	T	0.76	0.019	0.003	-0.006	0.005
rs2034768	A	0.49	0.015	0.002	-0.003	0.004
rs2045147	A	0.45	0.013	0.002	-0.001	0.004
rs2073869	C	0.83	0.019	0.003	0.007	0.006
rs2092829	G	0.71	0.014	0.002	0.000	0.005
rs2164744	T	0.64	-0.013	0.002	0.008	0.004
rs2173650	G	0.85	0.018	0.003	0.005	0.006
rs2184364	A	0.78	0.016	0.003	-0.001	0.005
rs2447098	C	0.48	-0.015	0.002	0.004	0.004
rs2460	G	0.74	-0.015	0.002	-0.001	0.005
rs2584597	T	0.66	0.015	0.002	-0.003	0.005
rs2616830	G	0.46	0.016	0.002	-0.008	0.004
rs262890	A	0.70	-0.019	0.002	-0.004	0.005
rs2717559	A	0.56	0.012	0.002	-0.005	0.004
rs2787374	T	0.41	0.012	0.002	-0.004	0.004
rs303753	G	0.65	-0.014	0.002	0.011	0.005
rs34864022	A	0.93	-0.026	0.004	-0.003	0.009
rs35574015	T	0.71	-0.013	0.002	0.008	0.005

rs374722	G	0.15	0.024	0.003	0.001	0.006
rs3754970	T	0.50	-0.015	0.002	-0.002	0.004
rs3796386	G	0.57	-0.026	0.002	0.002	0.004
rs405797	T	0.25	-0.015	0.003	-0.011	0.005
rs42210	G	0.29	-0.014	0.002	0.002	0.005
rs4334769	G	0.47	0.012	0.002	0.002	0.004
rs4382592	T	0.30	0.014	0.002	0.004	0.005
rs4523073	A	0.59	-0.014	0.002	0.010	0.004
rs4577309	A	0.47	0.016	0.002	-0.008	0.004
rs4675246	G	0.80	-0.015	0.003	0.006	0.005
rs4775373	T	0.36	0.013	0.002	-0.002	0.004
rs4845364	A	0.50	-0.015	0.002	-0.007	0.004
rs4937842	G	0.63	-0.013	0.002	0.008	0.004
rs4973576	C	0.30	-0.015	0.002	-0.002	0.005
rs55700114	G	0.71	-0.014	0.002	-0.002	0.005
rs55909997	G	0.65	-0.014	0.002	0.007	0.004
rs56103247	C	0.94	0.030	0.005	0.003	0.009
rs56858768	G	0.70	-0.015	0.002	-0.001	0.005
rs57585211	T	0.83	-0.017	0.003	0.000	0.006
rs6131281	C	0.60	0.016	0.002	-0.011	0.004
rs6141814	C	0.61	-0.014	0.002	-0.003	0.004
rs62379379	G	0.93	-0.026	0.004	-0.016	0.008
rs62641636	A	0.69	0.014	0.002	0.004	0.005
rs6472942	T	0.57	-0.013	0.002	-0.004	0.004
rs6673341	T	0.47	-0.015	0.002	0.002	0.004
rs66852340	C	0.78	-0.018	0.003	0.016	0.005
rs6721975	T	0.23	-0.017	0.003	0.007	0.005
rs6797840	A	0.46	-0.016	0.002	0.002	0.004
rs6825241	C	0.54	-0.017	0.002	0.000	0.004
rs6850494	A	0.62	-0.014	0.002	0.003	0.004
rs6905544	A	0.40	-0.019	0.002	0.004	0.004
rs6973656	A	0.60	-0.013	0.002	0.002	0.004
rs6996198	C	0.84	-0.016	0.003	0.001	0.006
rs7089973	C	0.62	-0.013	0.002	0.007	0.004
rs7157001	A	0.74	-0.014	0.002	-0.007	0.005
rs71658797	T	0.88	-0.020	0.003	0.019	0.007
rs7184800	G	0.70	0.017	0.002	-0.002	0.005
rs7189927	T	0.36	0.015	0.002	0.003	0.004
rs7248205	C	0.40	0.014	0.002	-0.007	0.004
rs72671494	T	0.86	-0.017	0.003	0.001	0.006
rs72781699	G	0.80	-0.019	0.003	-0.009	0.005
rs72828890	C	0.87	0.019	0.003	-0.005	0.007
rs72834698	G	0.86	0.023	0.003	-0.006	0.006
rs749671	G	0.63	0.016	0.002	0.004	0.004
rs7564130	T	0.64	-0.015	0.002	0.004	0.004
rs7693082	G	0.30	0.015	0.002	0.009	0.005
rs7693703	G	0.91	0.023	0.004	0.002	0.007

rs7700107	A	0.86	-0.021	0.003	-0.009	0.006
rs7716447	A	0.64	-0.013	0.002	-0.008	0.004
rs77215114	A	0.93	0.024	0.004	-0.008	0.009
rs7991062	C	0.66	-0.018	0.002	0.005	0.005
rs801733	A	0.64	0.017	0.002	0.000	0.004
rs8043253	C	0.57	-0.012	0.002	-0.002	0.004
rs8756	C	0.48	-0.013	0.002	-0.005	0.004
rs9471333	C	0.45	0.013	0.002	0.005	0.004
rs9563168	G	0.79	0.018	0.003	0.004	0.005
rs9569734	A	0.84	0.019	0.003	-0.011	0.006
rs9718104	T	0.94	-0.041	0.005	0.010	0.009
rs973734	C	0.15	0.017	0.003	-0.006	0.006
rs9834970	T	0.50	0.013	0.002	-0.002	0.004
rs984409	G	0.36	-0.015	0.002	0.004	0.004
rs9867121	C	0.82	0.019	0.003	-0.010	0.006
rs9902312	T	0.68	0.015	0.002	-0.008	0.005
rs9964724	C	0.32	0.018	0.002	0.010	0.005

Supplementary Table 20. Summary data for the genetic association between computer use and outcomes.

SNP	EA	EAF	GX	GX_SE	GY	GY_SE
AS						
rs10208088	C	0.42	0.013	0.002	0.000	0.009
rs10754920	C	0.11	0.020	0.003	0.006	0.014
rs113851275	G	0.89	-0.021	0.004	-0.040	0.015
rs11708955	T	0.69	-0.016	0.002	0.020	0.011
rs11749912	A	0.42	0.014	0.002	-0.005	0.009
rs12145677	G	0.70	-0.017	0.002	0.013	0.010
rs12603813	T	0.75	0.014	0.003	-0.001	0.011
rs12706626	G	0.62	-0.013	0.002	-0.003	0.010
rs13262595	A	0.44	-0.016	0.002	0.000	0.009
rs136553	C	0.62	-0.015	0.002	-0.002	0.010
rs1448355	C	0.38	-0.015	0.002	0.002	0.011
rs1469249	G	0.79	0.016	0.003	-0.028	0.011
rs162894	T	0.33	0.013	0.002	-0.007	0.010
rs166835	C	0.44	0.013	0.002	-0.005	0.009
rs198262	C	0.04	0.030	0.005	0.000	0.023
rs2068625	T	0.30	-0.016	0.002	0.005	0.010
rs206965	T	0.21	0.016	0.003	-0.001	0.012
rs2220599	C	0.63	-0.016	0.002	0.010	0.010
rs2345941	A	0.55	0.015	0.002	0.007	0.009
rs2734849	A	0.49	-0.014	0.002	0.002	0.009
rs2748985	T	0.45	-0.015	0.002	0.009	0.010
rs35933007	G	0.77	-0.015	0.003	-0.005	0.013
rs3730399	A	0.93	0.025	0.004	-0.006	0.018
rs3944151	A	0.28	0.014	0.002	0.001	0.011
rs4592851	G	0.75	-0.014	0.003	0.002	0.011
rs4702	G	0.44	0.012	0.002	-0.021	0.010
rs4977839	G	0.58	-0.020	0.002	0.022	0.010
rs55772938	A	0.70	-0.015	0.002	-0.025	0.011
rs6129084	A	0.36	-0.014	0.002	0.007	0.010
rs6498759	T	0.31	0.013	0.002	-0.009	0.010
rs66643547	C	0.65	-0.015	0.002	0.002	0.012
rs6744254	C	0.47	-0.016	0.002	-0.028	0.009
rs6774533	C	0.29	-0.015	0.002	-0.001	0.010
rs6857629	G	0.76	0.015	0.003	0.013	0.011
rs6935828	C	0.45	-0.012	0.002	0.004	0.009
rs7020477	A	0.73	0.014	0.002	-0.004	0.011
rs7209653	T	0.70	0.016	0.002	-0.004	0.010
rs7288455	A	0.44	0.013	0.002	-0.007	0.010
rs73578186	C	0.68	0.015	0.002	0.008	0.010
rs78082503	G	0.79	-0.015	0.003	0.009	0.011
rs784256	G	0.19	0.019	0.003	-0.012	0.012

rs9372625	G	0.62	-0.018	0.002	0.012	0.010
rs9477970	T	0.81	-0.016	0.003	0.010	0.012
AIS						
rs10208088	C	0.42	0.013	0.002	-0.004	0.010
rs10754920	C	0.11	0.020	0.003	0.011	0.015
rs113851275	G	0.89	-0.021	0.004	-0.047	0.016
rs11708955	T	0.69	-0.016	0.002	0.017	0.012
rs11749912	A	0.42	0.014	0.002	0.002	0.010
rs12145677	G	0.70	-0.017	0.002	0.010	0.011
rs12603813	T	0.75	0.014	0.003	-0.003	0.012
rs12706626	G	0.62	-0.013	0.002	-0.012	0.010
rs13262595	A	0.44	-0.016	0.002	0.000	0.010
rs136553	C	0.62	-0.015	0.002	-0.010	0.010
rs1448355	C	0.38	-0.015	0.002	0.007	0.011
rs1469249	G	0.79	0.016	0.003	-0.021	0.013
rs162894	T	0.33	0.013	0.002	-0.011	0.011
rs166835	C	0.44	0.013	0.002	-0.007	0.010
rs198262	C	0.04	0.030	0.005	-0.009	0.026
rs2068625	T	0.30	-0.016	0.002	0.003	0.011
rs206965	T	0.21	0.016	0.003	0.004	0.013
rs2220599	C	0.63	-0.016	0.002	0.011	0.010
rs2345941	A	0.55	0.015	0.002	0.003	0.010
rs2734849	A	0.49	-0.014	0.002	0.002	0.010
rs2748985	T	0.45	-0.015	0.002	0.010	0.010
rs35933007	G	0.77	-0.015	0.003	-0.010	0.014
rs3730399	A	0.93	0.025	0.004	0.008	0.020
rs3944151	A	0.28	0.014	0.002	0.005	0.011
rs4592851	G	0.75	-0.014	0.003	0.006	0.012
rs4702	G	0.44	0.012	0.002	-0.021	0.010
rs4977839	G	0.58	-0.020	0.002	0.020	0.010
rs55772938	A	0.70	-0.015	0.002	-0.030	0.011
rs6129084	A	0.36	-0.014	0.002	0.001	0.010
rs6498759	T	0.31	0.013	0.002	-0.012	0.011
rs66643547	C	0.65	-0.015	0.002	0.009	0.012
rs6744254	C	0.47	-0.016	0.002	-0.021	0.010
rs6774533	C	0.29	-0.015	0.002	-0.001	0.011
rs6857629	G	0.76	0.015	0.003	0.016	0.011
rs6935828	C	0.45	-0.012	0.002	0.000	0.010
rs7020477	A	0.73	0.014	0.002	-0.004	0.012
rs7209653	T	0.70	0.016	0.002	-0.005	0.011
rs7288455	A	0.44	0.013	0.002	-0.015	0.010
rs73578186	C	0.68	0.015	0.002	0.018	0.011
rs78082503	G	0.79	-0.015	0.003	0.001	0.012
rs784256	G	0.19	0.019	0.003	-0.011	0.013
rs9372625	G	0.62	-0.018	0.002	0.013	0.010

rs9477970	T	0.81	-0.016	0.003	0.014	0.013
CES						
rs10208088	C	0.42	0.013	0.002	-0.027	0.019
rs10754920	C	0.11	0.020	0.003	0.030	0.030
rs113851275	G	0.89	-0.021	0.004	-0.113	0.030
rs11708955	T	0.69	-0.016	0.002	0.023	0.022
rs11749912	A	0.42	0.014	0.002	0.012	0.020
rs12145677	G	0.70	-0.017	0.002	-0.009	0.021
rs12603813	T	0.75	0.014	0.003	-0.002	0.022
rs12706626	G	0.62	-0.013	0.002	-0.026	0.020
rs13262595	A	0.44	-0.016	0.002	0.007	0.019
rs136553	C	0.62	-0.015	0.002	-0.016	0.020
rs1448355	C	0.38	-0.015	0.002	0.024	0.022
rs1469249	G	0.79	0.016	0.003	-0.055	0.024
rs162894	T	0.33	0.013	0.002	-0.003	0.020
rs166835	C	0.44	0.013	0.002	0.000	0.020
rs198262	C	0.04	0.030	0.005	-0.050	0.054
rs2068625	T	0.30	-0.016	0.002	-0.015	0.022
rs206965	T	0.21	0.016	0.003	0.013	0.025
rs2220599	C	0.63	-0.016	0.002	-0.013	0.020
rs2345941	A	0.55	0.015	0.002	-0.020	0.019
rs2734849	A	0.49	-0.014	0.002	0.004	0.019
rs2748985	T	0.45	-0.015	0.002	0.016	0.020
rs35933007	G	0.77	-0.015	0.003	-0.047	0.025
rs3730399	A	0.93	0.025	0.004	-0.002	0.040
rs3944151	A	0.28	0.014	0.002	0.025	0.022
rs4592851	G	0.75	-0.014	0.003	0.008	0.022
rs4702	G	0.44	0.012	0.002	-0.019	0.020
rs4977839	G	0.58	-0.020	0.002	0.006	0.020
rs55772938	A	0.70	-0.015	0.002	-0.057	0.020
rs6129084	A	0.36	-0.014	0.002	0.004	0.020
rs6498759	T	0.31	0.013	0.002	-0.029	0.021
rs66643547	C	0.65	-0.015	0.002	-0.033	0.022
rs6744254	C	0.47	-0.016	0.002	0.002	0.019
rs6774533	C	0.29	-0.015	0.002	-0.004	0.021
rs6857629	G	0.76	0.015	0.003	0.006	0.022
rs6935828	C	0.45	-0.012	0.002	-0.001	0.019
rs7020477	A	0.73	0.014	0.002	0.006	0.023
rs7209653	T	0.70	0.016	0.002	-0.002	0.021
rs7288455	A	0.44	0.013	0.002	-0.014	0.020
rs73578186	C	0.68	0.015	0.002	0.021	0.020
rs78082503	G	0.79	-0.015	0.003	-0.013	0.023
rs784256	G	0.19	0.019	0.003	-0.036	0.026
rs9372625	G	0.62	-0.018	0.002	-0.018	0.020
rs9477970	T	0.81	-0.016	0.003	0.018	0.025

LAS						
rs10208088	C	0.42	0.013	0.002	0.010	0.025
rs10754920	C	0.11	0.020	0.003	-0.041	0.039
rs113851275	G	0.89	-0.021	0.004	0.010	0.040
rs11708955	T	0.69	-0.016	0.002	-0.035	0.029
rs11749912	A	0.42	0.014	0.002	0.013	0.025
rs12145677	G	0.70	-0.017	0.002	-0.003	0.028
rs12603813	T	0.75	0.014	0.003	-0.018	0.029
rs12706626	G	0.62	-0.013	0.002	0.012	0.026
rs13262595	A	0.44	-0.016	0.002	0.005	0.025
rs136553	C	0.62	-0.015	0.002	0.039	0.026
rs1448355	C	0.38	-0.015	0.002	0.009	0.029
rs1469249	G	0.79	0.016	0.003	0.006	0.031
rs162894	T	0.33	0.013	0.002	0.027	0.026
rs166835	C	0.44	0.013	0.002	-0.023	0.025
rs198262	C	0.04	0.030	0.005	-0.046	0.068
rs2068625	T	0.30	-0.016	0.002	-0.053	0.029
rs206965	T	0.21	0.016	0.003	-0.018	0.034
rs2220599	C	0.63	-0.016	0.002	0.036	0.026
rs2345941	A	0.55	0.015	0.002	0.024	0.025
rs2734849	A	0.49	-0.014	0.002	0.072	0.025
rs2748985	T	0.45	-0.015	0.002	0.053	0.026
rs35933007	G	0.77	-0.015	0.003	-0.008	0.032
rs3730399	A	0.93	0.025	0.004	-0.023	0.050
rs3944151	A	0.28	0.014	0.002	-0.018	0.028
rs4592851	G	0.75	-0.014	0.003	0.020	0.029
rs4702	G	0.44	0.012	0.002	-0.029	0.026
rs4977839	G	0.58	-0.020	0.002	0.004	0.026
rs55772938	A	0.70	-0.015	0.002	-0.040	0.027
rs6129084	A	0.36	-0.014	0.002	-0.026	0.026
rs6498759	T	0.31	0.013	0.002	0.010	0.027
rs66643547	C	0.65	-0.015	0.002	-0.014	0.029
rs6744254	C	0.47	-0.016	0.002	-0.031	0.025
rs6774533	C	0.29	-0.015	0.002	0.026	0.027
rs6857629	G	0.76	0.015	0.003	0.000	0.028
rs6935828	C	0.45	-0.012	0.002	-0.006	0.025
rs7020477	A	0.73	0.014	0.002	-0.032	0.029
rs7209653	T	0.70	0.016	0.002	0.000	0.027
rs7288455	A	0.44	0.013	0.002	0.013	0.026
rs73578186	C	0.68	0.015	0.002	0.072	0.026
rs78082503	G	0.79	-0.015	0.003	-0.008	0.030
rs784256	G	0.19	0.019	0.003	-0.033	0.033
rs9372625	G	0.62	-0.018	0.002	0.017	0.026
rs9477970	T	0.81	-0.016	0.003	0.054	0.032
SVS						

rs10208088	C	0.42	0.013	0.002	0.048	0.024
rs10754920	C	0.11	0.020	0.003	0.035	0.036
rs113851275	G	0.89	-0.021	0.004	-0.025	0.038
rs11708955	T	0.69	-0.016	0.002	0.038	0.028
rs11749912	A	0.42	0.014	0.002	0.053	0.024
rs12145677	G	0.70	-0.017	0.002	0.021	0.026
rs12603813	T	0.75	0.014	0.003	-0.002	0.027
rs12706626	G	0.62	-0.013	0.002	-0.024	0.024
rs13262595	A	0.44	-0.016	0.002	0.005	0.023
rs136553	C	0.62	-0.015	0.002	-0.004	0.025
rs1448355	C	0.38	-0.015	0.002	0.014	0.027
rs1469249	G	0.79	0.016	0.003	0.004	0.029
rs162894	T	0.33	0.013	0.002	-0.030	0.024
rs166835	C	0.44	0.013	0.002	0.001	0.023
rs198262	C	0.04	0.030	0.005	-0.055	0.061
rs2068625	T	0.30	-0.016	0.002	0.029	0.025
rs206965	T	0.21	0.016	0.003	0.028	0.030
rs2220599	C	0.63	-0.016	0.002	0.011	0.024
rs2345941	A	0.55	0.015	0.002	-0.024	0.023
rs2734849	A	0.49	-0.014	0.002	0.010	0.024
rs2748985	T	0.45	-0.015	0.002	0.029	0.024
rs35933007	G	0.77	-0.015	0.003	0.011	0.030
rs3730399	A	0.93	0.025	0.004	0.022	0.046
rs3944151	A	0.28	0.014	0.002	0.015	0.026
rs4592851	G	0.75	-0.014	0.003	0.001	0.027
rs4702	G	0.44	0.012	0.002	-0.023	0.025
rs4977839	G	0.58	-0.020	0.002	0.031	0.024
rs55772938	A	0.70	-0.015	0.002	-0.019	0.025
rs6129084	A	0.36	-0.014	0.002	0.042	0.024
rs6498759	T	0.31	0.013	0.002	-0.024	0.025
rs66643547	C	0.65	-0.015	0.002	0.061	0.027
rs6744254	C	0.47	-0.016	0.002	-0.006	0.023
rs6774533	C	0.29	-0.015	0.002	0.031	0.026
rs6857629	G	0.76	0.015	0.003	0.027	0.027
rs6935828	C	0.45	-0.012	0.002	0.016	0.023
rs7020477	A	0.73	0.014	0.002	0.043	0.028
rs7209653	T	0.70	0.016	0.002	-0.005	0.025
rs7288455	A	0.44	0.013	0.002	0.038	0.024
rs73578186	C	0.68	0.015	0.002	-0.016	0.024
rs78082503	G	0.79	-0.015	0.003	-0.008	0.028
rs784256	G	0.19	0.019	0.003	0.011	0.031
rs9372625	G	0.62	-0.018	0.002	0.048	0.024
rs9477970	T	0.81	-0.016	0.003	0.040	0.030
ICH						
rs10754920	C	0.11	0.020	0.003	0.020	0.079

rs113851275	G	0.89	-0.021	0.004	0.155	0.082
rs11708955	T	0.69	-0.016	0.002	0.014	0.057
rs11749912	A	0.42	0.014	0.002	-0.081	0.053
rs12145677	G	0.70	-0.017	0.002	-0.022	0.056
rs12603813	T	0.75	0.014	0.003	-0.049	0.059
rs13262595	A	0.44	-0.016	0.002	0.021	0.052
rs136553	C	0.62	-0.015	0.002	0.030	0.053
rs162894	T	0.33	0.013	0.002	-0.004	0.053
rs166835	C	0.44	0.013	0.002	-0.055	0.052
rs198262	C	0.04	0.030	0.005	-0.018	0.134
rs2068625	T	0.30	-0.016	0.002	0.056	0.055
rs206965	T	0.21	0.016	0.003	-0.035	0.063
rs2345941	A	0.55	0.015	0.002	0.008	0.052
rs2734849	A	0.49	-0.014	0.002	0.036	0.052
rs3730399	A	0.93	0.025	0.004	-0.037	0.104
rs3944151	A	0.28	0.014	0.002	-0.049	0.058
rs4592851	G	0.75	-0.014	0.003	-0.074	0.060
rs4977839	G	0.58	-0.020	0.002	-0.016	0.054
rs55772938	A	0.70	-0.015	0.002	-0.013	0.056
rs6129084	A	0.36	-0.014	0.002	0.056	0.054
rs6498759	T	0.31	0.013	0.002	-0.017	0.055
rs6744254	C	0.47	-0.016	0.002	0.005	0.051
rs6774533	C	0.29	-0.015	0.002	0.020	0.054
rs6857629	G	0.76	0.015	0.003	-0.089	0.059
rs6935828	C	0.45	-0.012	0.002	0.034	0.051
rs7209653	T	0.70	0.016	0.002	-0.054	0.057
rs7288455	A	0.44	0.013	0.002	0.028	0.052
rs73578186	C	0.68	0.015	0.002	-0.029	0.053
rs78082503	G	0.79	-0.015	0.003	0.029	0.062
rs784256	G	0.19	0.019	0.003	-0.039	0.066
rs9372625	G	0.62	-0.018	0.002	0.058	0.053
rs9477970	T	0.81	-0.016	0.003	-0.024	0.066
non-lobar ICH						
rs10754920	C	0.11	0.020	0.003	-0.013	0.095
rs113851275	G	0.89	-0.021	0.004	0.187	0.098
rs11708955	T	0.69	-0.016	0.002	0.031	0.068
rs11749912	A	0.42	0.014	0.002	-0.077	0.063
rs12145677	G	0.70	-0.017	0.002	-0.033	0.067
rs12603813	T	0.75	0.014	0.003	-0.032	0.070
rs13262595	A	0.44	-0.016	0.002	0.057	0.062
rs136553	C	0.62	-0.015	0.002	0.005	0.063
rs162894	T	0.33	0.013	0.002	-0.048	0.064
rs166835	C	0.44	0.013	0.002	-0.065	0.061
rs198262	C	0.04	0.030	0.005	0.025	0.159
rs2068625	T	0.30	-0.016	0.002	0.071	0.066

rs206965	T	0.21	0.016	0.003	-0.013	0.074
rs2345941	A	0.55	0.015	0.002	-0.038	0.061
rs2734849	A	0.49	-0.014	0.002	0.029	0.062
rs3730399	A	0.93	0.025	0.004	-0.022	0.123
rs3944151	A	0.28	0.014	0.002	0.001	0.069
rs4592851	G	0.75	-0.014	0.003	-0.029	0.072
rs4977839	G	0.58	-0.020	0.002	0.053	0.064
rs55772938	A	0.70	-0.015	0.002	-0.011	0.066
rs6129084	A	0.36	-0.014	0.002	0.060	0.064
rs6498759	T	0.31	0.013	0.002	-0.009	0.066
rs6744254	C	0.47	-0.016	0.002	-0.013	0.061
rs6774533	C	0.29	-0.015	0.002	0.024	0.065
rs6857629	G	0.76	0.015	0.003	-0.047	0.069
rs6935828	C	0.45	-0.012	0.002	0.038	0.060
rs7209653	T	0.70	0.016	0.002	-0.105	0.066
rs7288455	A	0.44	0.013	0.002	0.016	0.063
rs73578186	C	0.68	0.015	0.002	-0.015	0.063
rs78082503	G	0.79	-0.015	0.003	0.063	0.074
rs784256	G	0.19	0.019	0.003	-0.046	0.079
rs9372625	G	0.62	-0.018	0.002	0.070	0.063
rs9477970	T	0.81	-0.016	0.003	0.016	0.079
lobar ICH						
rs10754920	C	0.11	0.020	0.003	0.125	0.102
rs113851275	G	0.89	-0.021	0.004	0.140	0.110
rs11708955	T	0.69	-0.016	0.002	0.026	0.073
rs11749912	A	0.42	0.014	0.002	-0.091	0.068
rs12145677	G	0.70	-0.017	0.002	-0.015	0.073
rs13262595	A	0.44	-0.016	0.002	0.001	0.068
rs136553	C	0.62	-0.015	0.002	0.078	0.069
rs162894	T	0.33	0.013	0.002	0.011	0.068
rs166835	C	0.44	0.013	0.002	-0.017	0.068
rs198262	C	0.04	0.030	0.005	-0.048	0.179
rs2068625	T	0.30	-0.016	0.002	0.045	0.071
rs206965	T	0.21	0.016	0.003	-0.115	0.082
rs2345941	A	0.55	0.015	0.002	0.084	0.069
rs2734849	A	0.49	-0.014	0.002	0.035	0.068
rs3730399	A	0.93	0.025	0.004	-0.014	0.138
rs3944151	A	0.28	0.014	0.002	-0.097	0.076
rs4592851	G	0.75	-0.014	0.003	-0.150	0.077
rs4977839	G	0.58	-0.020	0.002	-0.110	0.070
rs55772938	A	0.70	-0.015	0.002	-0.015	0.074
rs6129084	A	0.36	-0.014	0.002	0.074	0.070
rs6498759	T	0.31	0.013	0.002	-0.061	0.072
rs6744254	C	0.47	-0.016	0.002	0.029	0.067
rs6774533	C	0.29	-0.015	0.002	-0.013	0.072

rs6857629	G	0.76	0.015	0.003	-0.161	0.076
rs6935828	C	0.45	-0.012	0.002	0.037	0.067
rs7209653	T	0.70	0.016	0.002	0.029	0.074
rs73578186	C	0.68	0.015	0.002	-0.040	0.070
rs78082503	G	0.79	-0.015	0.003	0.000	0.079
rs784256	G	0.19	0.019	0.003	-0.028	0.087
rs9477970	T	0.81	-0.016	0.003	-0.068	0.085
WMH						
rs10208088	C	0.42	0.013	0.002	-0.007	0.013
rs10754920	C	0.11	0.020	0.003	0.000	0.020
rs113851275	G	0.89	-0.021	0.004	-0.042	0.020
rs11749912	A	0.42	0.014	0.002	-0.025	0.013
rs12145677	G	0.70	-0.017	0.002	-0.005	0.014
rs12603813	T	0.75	0.014	0.003	0.063	0.014
rs12706626	G	0.62	-0.013	0.002	0.004	0.013
rs13262595	A	0.44	-0.016	0.002	-0.001	0.013
rs136553	C	0.62	-0.015	0.002	-0.023	0.013
rs1448355	C	0.38	-0.015	0.002	-0.001	0.013
rs1469249	G	0.79	0.016	0.003	-0.009	0.015
rs162894	T	0.33	0.013	0.002	0.015	0.013
rs166835	C	0.44	0.013	0.002	0.002	0.013
rs198262	C	0.04	0.030	0.005	-0.027	0.031
rs2068625	T	0.30	-0.016	0.002	0.014	0.014
rs206965	T	0.21	0.016	0.003	0.002	0.015
rs2220599	C	0.63	-0.016	0.002	0.004	0.013
rs2345941	A	0.55	0.015	0.002	-0.008	0.012
rs2734849	A	0.49	-0.014	0.002	-0.005	0.012
rs2748985	T	0.45	-0.015	0.002	-0.033	0.013
rs35933007	G	0.77	-0.015	0.003	-0.004	0.015
rs3730399	A	0.93	0.025	0.004	0.015	0.025
rs3944151	A	0.28	0.014	0.002	0.000	0.014
rs4702	G	0.44	0.012	0.002	-0.003	0.012
rs4977839	G	0.58	-0.020	0.002	-0.003	0.013
rs55772938	A	0.70	-0.015	0.002	-0.013	0.014
rs6129084	A	0.36	-0.014	0.002	-0.021	0.013
rs6498759	T	0.31	0.013	0.002	-0.013	0.014
rs66643547	C	0.65	-0.015	0.002	0.007	0.013
rs6744254	C	0.47	-0.016	0.002	-0.005	0.012
rs6774533	C	0.29	-0.015	0.002	0.010	0.014
rs6857629	G	0.76	0.015	0.003	0.023	0.014
rs6935828	C	0.45	-0.012	0.002	0.022	0.012
rs7020477	A	0.73	0.014	0.002	0.022	0.014
rs7209653	T	0.70	0.016	0.002	-0.014	0.014
rs7288455	A	0.44	0.013	0.002	-0.006	0.012
rs73578186	C	0.68	0.015	0.002	0.007	0.013

rs78082503	G	0.79	-0.015	0.003	-0.025	0.015
rs784256	G	0.19	0.019	0.003	0.027	0.016
rs9372625	G	0.62	-0.018	0.002	0.030	0.013
rs9477970	T	0.81	-0.016	0.003	0.008	0.015
AD						
rs10208088	C	0.42	0.013	0.002	-0.021	0.014
rs10754920	C	0.11	0.020	0.003	-0.015	0.022
rs113851275	G	0.89	-0.021	0.004	-0.037	0.025
rs11708955	T	0.69	-0.016	0.002	0.013	0.016
rs11749912	A	0.42	0.014	0.002	-0.038	0.015
rs12145677	G	0.70	-0.017	0.002	0.008	0.016
rs12603813	T	0.75	0.014	0.003	-0.010	0.017
rs12706626	G	0.62	-0.013	0.002	-0.006	0.015
rs13262595	A	0.44	-0.016	0.002	0.019	0.014
rs136553	C	0.62	-0.015	0.002	-0.007	0.015
rs1448355	C	0.38	-0.015	0.002	0.018	0.015
rs1469249	G	0.79	0.016	0.003	0.002	0.018
rs162894	T	0.33	0.013	0.002	0.006	0.015
rs166835	C	0.44	0.013	0.002	-0.012	0.014
rs198262	C	0.04	0.030	0.005	0.020	0.036
rs2068625	T	0.30	-0.016	0.002	0.014	0.016
rs206965	T	0.21	0.016	0.003	-0.011	0.017
rs2220599	C	0.63	-0.016	0.002	0.020	0.015
rs2345941	A	0.55	0.015	0.002	-0.010	0.014
rs2734849	A	0.49	-0.014	0.002	-0.008	0.014
rs2748985	T	0.45	-0.015	0.002	-0.004	0.015
rs35933007	G	0.77	-0.015	0.003	-0.023	0.020
rs3730399	A	0.93	0.025	0.004	-0.014	0.030
rs3944151	A	0.28	0.014	0.002	0.047	0.016
rs4592851	G	0.75	-0.014	0.003	-0.005	0.017
rs4702	G	0.44	0.012	0.002	-0.022	0.015
rs4977839	G	0.58	-0.020	0.002	0.029	0.016
rs55772938	A	0.70	-0.015	0.002	0.015	0.016
rs6129084	A	0.36	-0.014	0.002	-0.015	0.015
rs6498759	T	0.31	0.013	0.002	-0.002	0.015
rs66643547	C	0.65	-0.015	0.002	0.020	0.018
rs6744254	C	0.47	-0.016	0.002	0.019	0.014
rs6774533	C	0.29	-0.015	0.002	0.006	0.015
rs6857629	G	0.76	0.015	0.003	0.004	0.017
rs7020477	A	0.73	0.014	0.002	-0.010	0.017
rs7209653	T	0.70	0.016	0.002	-0.036	0.016
rs7288455	A	0.44	0.013	0.002	-0.003	0.015
rs73578186	C	0.68	0.015	0.002	-0.007	0.015
rs78082503	G	0.79	-0.015	0.003	0.027	0.017
rs784256	G	0.19	0.019	0.003	0.011	0.018

rs9372625	G	0.62	-0.018	0.002	0.032	0.015
rs9477970	T	0.81	-0.016	0.003	0.018	0.018
PD						
rs10208088	C	0.42	0.013	0.002	0.002	0.023
rs10754920	C	0.11	0.020	0.003	0.044	0.034
rs113851275	G	0.89	-0.021	0.004	-0.017	0.030
rs11749912	A	0.42	0.014	0.002	0.010	0.023
rs12145677	G	0.70	-0.017	0.002	0.001	0.020
rs12603813	T	0.75	0.014	0.003	-0.037	0.022
rs12706626	G	0.62	-0.013	0.002	0.027	0.019
rs13262595	A	0.44	-0.016	0.002	0.003	0.018
rs136553	C	0.62	-0.015	0.002	0.005	0.023
rs1448355	C	0.38	-0.015	0.002	0.007	0.023
rs1469249	G	0.79	0.016	0.003	0.076	0.027
rs162894	T	0.33	0.013	0.002	-0.001	0.019
rs166835	C	0.44	0.013	0.002	-0.016	0.023
rs198262	C	0.04	0.030	0.005	0.027	0.057
rs2068625	T	0.30	-0.016	0.002	-0.004	0.021
rs206965	T	0.21	0.016	0.003	-0.028	0.022
rs2220599	C	0.63	-0.016	0.002	0.005	0.023
rs2345941	A	0.55	0.015	0.002	0.014	0.020
rs2734849	A	0.49	-0.014	0.002	0.032	0.017
rs2748985	T	0.45	-0.015	0.002	0.010	0.019
rs35933007	G	0.77	-0.015	0.003	0.004	0.025
rs3730399	A	0.93	0.025	0.004	0.049	0.035
rs3944151	A	0.28	0.014	0.002	0.048	0.025
rs4702	G	0.44	0.012	0.002	0.028	0.020
rs4977839	G	0.58	-0.020	0.002	0.016	0.023
rs55772938	A	0.70	-0.015	0.002	0.014	0.025
rs6129084	A	0.36	-0.014	0.002	-0.023	0.018
rs6498759	T	0.31	0.013	0.002	-0.018	0.024
rs66643547	C	0.65	-0.015	0.002	0.015	0.026
rs6744254	C	0.47	-0.016	0.002	0.018	0.020
rs6774533	C	0.29	-0.015	0.002	-0.034	0.029
rs6857629	G	0.76	0.015	0.003	-0.036	0.022
rs6935828	C	0.45	-0.012	0.002	-0.002	0.022
rs7020477	A	0.73	0.014	0.002	-0.007	0.023
rs7209653	T	0.70	0.016	0.002	-0.011	0.020
rs7288455	A	0.44	0.013	0.002	0.004	0.023
rs73578186	C	0.68	0.015	0.002	-0.026	0.023
rs78082503	G	0.79	-0.015	0.003	-0.014	0.027
rs784256	G	0.19	0.019	0.003	-0.021	0.024
rs9372625	G	0.62	-0.018	0.002	-0.051	0.023
rs9477970	T	0.81	-0.016	0.003	0.017	0.029
MS						

rs10208088	C	0.42	0.013	0.002	0.011	0.193
rs10754920	C	0.11	0.020	0.003	0.024	0.061
rs11708955	T	0.69	-0.016	0.002	-0.005	0.008
rs11749912	A	0.42	0.014	0.002	0.032	0.020
rs12145677	G	0.70	-0.017	0.002	0.009	0.039
rs12603813	T	0.75	0.014	0.003	-0.015	0.094
rs12706626	G	0.62	-0.013	0.002	-0.042	0.019
rs13262595	A	0.44	-0.016	0.002	-0.024	0.022
rs136553	C	0.62	-0.015	0.002	-0.021	0.026
rs1448355	C	0.38	-0.015	0.002	-0.002	0.001
rs1469249	G	0.79	0.016	0.003	0.034	0.026
rs162894	T	0.33	0.013	0.002	0.000	0.000
rs166835	C	0.44	0.013	0.002	-0.012	0.115
rs198262	C	0.04	0.030	0.005	0.058	0.055
rs2068625	T	0.30	-0.016	0.002	-0.018	0.039
rs206965	T	0.21	0.016	0.003	0.038	0.024
rs2220599	C	0.63	-0.016	0.002	-0.012	0.254
rs2345941	A	0.55	0.015	0.002	0.011	0.384
rs2734849	A	0.49	-0.014	0.002	0.019	0.027
rs2748985	T	0.45	-0.015	0.002	0.049	0.019
rs35933007	G	0.77	-0.015	0.003	0.000	0.000
rs3730399	A	0.93	0.025	0.004	-0.027	0.110
rs3944151	A	0.28	0.014	0.002	0.048	0.020
rs4592851	G	0.75	-0.014	0.003	0.006	0.009
rs4702	G	0.44	0.012	0.002	0.003	0.003
rs4977839	G	0.58	-0.020	0.002	-0.007	0.013
rs55772938	A	0.70	-0.015	0.002	-0.014	0.084
rs6129084	A	0.36	-0.014	0.002	-0.010	0.084
rs6498759	T	0.31	0.013	0.002	-0.002	0.002
rs66643547	C	0.65	-0.015	0.002	-0.014	0.032
rs6744254	C	0.47	-0.016	0.002	-0.018	0.029
rs6774533	C	0.29	-0.015	0.002	0.019	0.033
rs6857629	G	0.76	0.015	0.003	-0.027	0.025
rs6935828	C	0.45	-0.012	0.002	-0.009	0.032
rs7020477	A	0.73	0.014	0.002	-0.001	0.000
rs7209653	T	0.70	0.016	0.002	0.005	0.006
rs7288455	A	0.44	0.013	0.002	-0.014	0.074
rs73578186	C	0.68	0.015	0.002	0.014	0.072
rs784256	G	0.19	0.019	0.003	0.026	0.034
rs9372625	G	0.62	-0.018	0.002	0.022	0.026
rs9477970	T	0.81	-0.016	0.003	0.006	0.009
SBP						
rs10208088	C	0.42	0.013	0.002	-0.097	0.031
rs10754920	C	0.11	0.020	0.003	-0.166	0.047
rs113851275	G	0.89	-0.021	0.004	0.033	0.049

rs11749912	A	0.42	0.014	0.002	0.085	0.031
rs12145677	G	0.70	-0.017	0.002	-0.043	0.033
rs12603813	T	0.75	0.014	0.003	-0.433	0.035
rs12706626	G	0.62	-0.013	0.002	-0.026	0.032
rs13262595	A	0.44	-0.016	0.002	0.185	0.031
rs136553	C	0.62	-0.015	0.002	0.026	0.031
rs1448355	C	0.38	-0.015	0.002	-0.083	0.031
rs1469249	G	0.79	0.016	0.003	-0.156	0.037
rs162894	T	0.33	0.013	0.002	0.038	0.032
rs166835	C	0.44	0.013	0.002	-0.103	0.030
rs198262	C	0.04	0.030	0.005	-0.036	0.074
rs2068625	T	0.30	-0.016	0.002	0.019	0.033
rs206965	T	0.21	0.016	0.003	-0.025	0.037
rs2220599	C	0.63	-0.016	0.002	0.122	0.031
rs2345941	A	0.55	0.015	0.002	-0.026	0.030
rs2734849	A	0.49	-0.014	0.002	0.057	0.030
rs2748985	T	0.45	-0.015	0.002	0.285	0.031
rs35933007	G	0.77	-0.015	0.003	-0.006	0.038
rs3730399	A	0.93	0.025	0.004	-0.037	0.060
rs3944151	A	0.28	0.014	0.002	0.002	0.034
rs4702	G	0.44	0.012	0.002	-0.432	0.031
rs4977839	G	0.58	-0.020	0.002	0.006	0.031
rs55772938	A	0.70	-0.015	0.002	-0.013	0.033
rs6129084	A	0.36	-0.014	0.002	-0.009	0.032
rs6498759	T	0.31	0.013	0.002	-0.051	0.032
rs66643547	C	0.65	-0.015	0.002	0.197	0.033
rs6744254	C	0.47	-0.016	0.002	0.044	0.030
rs6774533	C	0.29	-0.015	0.002	0.075	0.034
rs6857629	G	0.76	0.015	0.003	-0.003	0.035
rs6935828	C	0.45	-0.012	0.002	-0.032	0.030
rs7020477	A	0.73	0.014	0.002	0.058	0.035
rs7209653	T	0.70	0.016	0.002	-0.130	0.033
rs7288455	A	0.44	0.013	0.002	-0.044	0.031
rs73578186	C	0.68	0.015	0.002	-0.010	0.032
rs78082503	G	0.79	-0.015	0.003	0.076	0.036
rs784256	G	0.19	0.019	0.003	0.199	0.039
rs9372625	G	0.62	-0.018	0.002	0.133	0.031
rs9477970	T	0.81	-0.016	0.003	0.092	0.039
DBP						
rs10208088	C	0.42	0.013	0.002	-0.044	0.018
rs10754920	C	0.11	0.020	0.003	-0.089	0.027
rs113851275	G	0.89	-0.021	0.004	-0.046	0.028
rs11749912	A	0.42	0.014	0.002	0.032	0.018
rs12145677	G	0.70	-0.017	0.002	0.011	0.019
rs12603813	T	0.75	0.014	0.003	-0.173	0.020

rs12706626	G	0.62	-0.013	0.002	0.009	0.018
rs13262595	A	0.44	-0.016	0.002	0.092	0.018
rs136553	C	0.62	-0.015	0.002	0.012	0.018
rs1448355	C	0.38	-0.015	0.002	-0.056	0.018
rs1469249	G	0.79	0.016	0.003	-0.072	0.021
rs162894	T	0.33	0.013	0.002	0.121	0.018
rs166835	C	0.44	0.013	0.002	-0.055	0.018
rs198262	C	0.04	0.030	0.005	0.035	0.043
rs2068625	T	0.30	-0.016	0.002	0.002	0.019
rs206965	T	0.21	0.016	0.003	-0.020	0.021
rs2220599	C	0.63	-0.016	0.002	0.054	0.018
rs2345941	A	0.55	0.015	0.002	-0.034	0.017
rs2734849	A	0.49	-0.014	0.002	0.027	0.017
rs2748985	T	0.45	-0.015	0.002	0.120	0.018
rs35933007	G	0.77	-0.015	0.003	0.035	0.022
rs3730399	A	0.93	0.025	0.004	-0.058	0.035
rs3944151	A	0.28	0.014	0.002	0.049	0.020
rs4702	G	0.44	0.012	0.002	-0.211	0.018
rs4977839	G	0.58	-0.020	0.002	0.002	0.018
rs55772938	A	0.70	-0.015	0.002	0.006	0.019
rs6129084	A	0.36	-0.014	0.002	-0.016	0.018
rs6498759	T	0.31	0.013	0.002	-0.052	0.019
rs66643547	C	0.65	-0.015	0.002	0.110	0.019
rs6744254	C	0.47	-0.016	0.002	0.038	0.017
rs6774533	C	0.29	-0.015	0.002	0.030	0.019
rs6857629	G	0.76	0.015	0.003	-0.006	0.020
rs6935828	C	0.45	-0.012	0.002	0.035	0.017
rs7020477	A	0.73	0.014	0.002	0.016	0.020
rs7209653	T	0.70	0.016	0.002	-0.036	0.019
rs7288455	A	0.44	0.013	0.002	-0.020	0.018
rs73578186	C	0.68	0.015	0.002	-0.004	0.018
rs78082503	G	0.79	-0.015	0.003	0.012	0.021
rs784256	G	0.19	0.019	0.003	0.090	0.022
rs9372625	G	0.62	-0.018	0.002	0.060	0.018
rs9477970	T	0.81	-0.016	0.003	0.023	0.022
PP						
rs10208088	C	0.42	0.013	0.002	-0.043	0.021
rs10754920	C	0.11	0.020	0.003	-0.063	0.032
rs113851275	G	0.89	-0.021	0.004	0.069	0.034
rs11749912	A	0.42	0.014	0.002	0.062	0.021
rs12145677	G	0.70	-0.017	0.002	-0.052	0.022
rs12603813	T	0.75	0.014	0.003	-0.268	0.024
rs12706626	G	0.62	-0.013	0.002	-0.025	0.021
rs13262595	A	0.44	-0.016	0.002	0.094	0.021
rs136553	C	0.62	-0.015	0.002	0.017	0.021

rs1448355	C	0.38	-0.015	0.002	-0.025	0.021
rs1469249	G	0.79	0.016	0.003	-0.096	0.025
rs162894	T	0.33	0.013	0.002	-0.068	0.022
rs166835	C	0.44	0.013	0.002	-0.047	0.021
rs198262	C	0.04	0.030	0.005	-0.058	0.051
rs2068625	T	0.30	-0.016	0.002	0.012	0.022
rs206965	T	0.21	0.016	0.003	-0.005	0.025
rs2220599	C	0.63	-0.016	0.002	0.065	0.021
rs2345941	A	0.55	0.015	0.002	0.002	0.021
rs2734849	A	0.49	-0.014	0.002	0.037	0.021
rs2748985	T	0.45	-0.015	0.002	0.158	0.021
rs35933007	G	0.77	-0.015	0.003	-0.036	0.026
rs3730399	A	0.93	0.025	0.004	0.017	0.041
rs3944151	A	0.28	0.014	0.002	-0.042	0.023
rs4702	G	0.44	0.012	0.002	-0.223	0.021
rs4977839	G	0.58	-0.020	0.002	0.006	0.021
rs55772938	A	0.70	-0.015	0.002	-0.013	0.023
rs6129084	A	0.36	-0.014	0.002	0.009	0.021
rs6498759	T	0.31	0.013	0.002	-0.004	0.022
rs66643547	C	0.65	-0.015	0.002	0.091	0.023
rs6744254	C	0.47	-0.016	0.002	0.011	0.021
rs6774533	C	0.29	-0.015	0.002	0.057	0.023
rs6857629	G	0.76	0.015	0.003	-0.001	0.024
rs6935828	C	0.45	-0.012	0.002	-0.066	0.021
rs7020477	A	0.73	0.014	0.002	0.043	0.024
rs7209653	T	0.70	0.016	0.002	-0.095	0.022
rs7288455	A	0.44	0.013	0.002	-0.027	0.021
rs73578186	C	0.68	0.015	0.002	-0.005	0.022
rs78082503	G	0.79	-0.015	0.003	0.053	0.025
rs784256	G	0.19	0.019	0.003	0.116	0.026
rs9372625	G	0.62	-0.018	0.002	0.072	0.021
rs9477970	T	0.81	-0.016	0.003	0.068	0.026
HDL-C						
rs12145677	G	0.70	-0.017	0.002	-0.004	0.005
rs2734849	A	0.49	-0.014	0.002	0.001	0.005
rs1448355	C	0.38	-0.015	0.002	0.002	0.005
rs206965	T	0.21	0.016	0.003	0.003	0.006
rs1319734	A	0.60	0.013	0.002	0.000	0.005
rs198262	C	0.04	0.030	0.005	0.000	0.012
rs166835	C	0.44	0.013	0.002	0.008	0.005
rs4702	G	0.44	0.012	0.002	-0.011	0.004
rs6498759	T	0.31	0.013	0.002	0.003	0.005
rs4073003	A	0.87	0.020	0.003	-0.003	0.008
rs7209653	T	0.70	0.016	0.002	0.005	0.005
rs12603813	T	0.75	0.014	0.003	0.003	0.004

rs784256	G	0.19	0.019	0.003	0.004	0.006
rs6744254	C	0.47	-0.016	0.002	0.001	0.005
rs2068625	T	0.30	-0.016	0.002	0.001	0.005
rs1469249	G	0.79	0.016	0.003	0.010	0.006
rs162894	T	0.33	0.013	0.002	-0.008	0.005
rs17789218	T	0.76	-0.015	0.003	-0.004	0.004
rs6935828	C	0.45	-0.012	0.002	-0.004	0.005
rs9372625	G	0.62	-0.018	0.002	-0.008	0.005
rs3944151	A	0.28	0.014	0.002	0.000	0.005
rs2345941	A	0.55	0.015	0.002	-0.001	0.005
rs13262595	A	0.44	-0.016	0.002	-0.002	0.005
rs9477970	T	0.81	-0.016	0.003	0.010	0.006
rs2220599	C	0.63	-0.016	0.002	-0.002	0.005
LDL-C						
rs12145677	G	0.70	-0.017	0.002	0.026	0.005
rs2734849	A	0.49	-0.014	0.002	-0.001	0.005
rs1448355	C	0.38	-0.015	0.002	-0.003	0.006
rs206965	T	0.21	0.016	0.003	0.004	0.006
rs1319734	A	0.60	0.013	0.002	-0.001	0.005
rs198262	C	0.04	0.030	0.005	0.018	0.013
rs166835	C	0.44	0.013	0.002	-0.005	0.005
rs4702	G	0.44	0.012	0.002	-0.001	0.004
rs6498759	T	0.31	0.013	0.002	-0.008	0.006
rs4073003	A	0.87	0.020	0.003	-0.001	0.009
rs7209653	T	0.70	0.016	0.002	-0.003	0.006
rs12603813	T	0.75	0.014	0.003	0.005	0.004
rs784256	G	0.19	0.019	0.003	-0.005	0.007
rs6744254	C	0.47	-0.016	0.002	0.003	0.006
rs2068625	T	0.30	-0.016	0.002	0.007	0.006
rs1469249	G	0.79	0.016	0.003	0.009	0.006
rs162894	T	0.33	0.013	0.002	-0.002	0.005
rs17789218	T	0.76	-0.015	0.003	0.024	0.004
rs6935828	C	0.45	-0.012	0.002	-0.003	0.005
rs9372625	G	0.62	-0.018	0.002	0.017	0.005
rs3944151	A	0.28	0.014	0.002	0.000	0.006
rs2345941	A	0.55	0.015	0.002	0.007	0.005
rs13262595	A	0.44	-0.016	0.002	0.000	0.005
rs9477970	T	0.81	-0.016	0.003	-0.007	0.007
rs2220599	C	0.63	-0.016	0.002	0.005	0.006
TC						
rs12145677	G	0.70	-0.017	0.002	0.018	0.005
rs2734849	A	0.49	-0.014	0.002	0.000	0.005
rs1448355	C	0.38	-0.015	0.002	-0.004	0.006
rs206965	T	0.21	0.016	0.003	0.006	0.006
rs1319734	A	0.60	0.013	0.002	-0.003	0.005

rs198262	C	0.04	0.030	0.005	0.012	0.013
rs166835	C	0.44	0.013	0.002	0.001	0.005
rs4702	G	0.44	0.012	0.002	-0.002	0.004
rs6498759	T	0.31	0.013	0.002	-0.005	0.006
rs4073003	A	0.87	0.020	0.003	-0.001	0.009
rs7209653	T	0.70	0.016	0.002	-0.003	0.006
rs12603813	T	0.75	0.014	0.003	0.004	0.004
rs784256	G	0.19	0.019	0.003	-0.007	0.007
rs6744254	C	0.47	-0.016	0.002	0.006	0.005
rs2068625	T	0.30	-0.016	0.002	0.009	0.006
rs1469249	G	0.79	0.016	0.003	0.010	0.006
rs162894	T	0.33	0.013	0.002	-0.001	0.005
rs17789218	T	0.76	-0.015	0.003	0.020	0.004
rs6935828	C	0.45	-0.012	0.002	-0.007	0.005
rs9372625	G	0.62	-0.018	0.002	0.013	0.005
rs3944151	A	0.28	0.014	0.002	0.001	0.006
rs2345941	A	0.55	0.015	0.002	0.006	0.005
rs13262595	A	0.44	-0.016	0.002	-0.001	0.005
rs9477970	T	0.81	-0.016	0.003	-0.007	0.007
rs2220599	C	0.63	-0.016	0.002	0.005	0.005
TG						
rs12145677	G	0.70	-0.017	0.002	0.003	0.004
rs2734849	A	0.49	-0.014	0.002	0.003	0.005
rs1448355	C	0.38	-0.015	0.002	-0.005	0.005
rs206965	T	0.21	0.016	0.003	-0.008	0.006
rs1319734	A	0.60	0.013	0.002	0.008	0.005
rs198262	C	0.04	0.030	0.005	-0.010	0.012
rs166835	C	0.44	0.013	0.002	-0.007	0.005
rs4702	G	0.44	0.012	0.002	0.006	0.004
rs6498759	T	0.31	0.013	0.002	-0.002	0.005
rs4073003	A	0.87	0.020	0.003	0.010	0.008
rs7209653	T	0.70	0.016	0.002	0.000	0.005
rs12603813	T	0.75	0.014	0.003	-0.013	0.004
rs784256	G	0.19	0.019	0.003	-0.006	0.006
rs6744254	C	0.47	-0.016	0.002	0.001	0.005
rs2068625	T	0.30	-0.016	0.002	0.010	0.005
rs1469249	G	0.79	0.016	0.003	0.003	0.006
rs162894	T	0.33	0.013	0.002	0.013	0.005
rs17789218	T	0.76	-0.015	0.003	0.006	0.004
rs6935828	C	0.45	-0.012	0.002	-0.007	0.005
rs9372625	G	0.62	-0.018	0.002	0.005	0.005
rs3944151	A	0.28	0.014	0.002	-0.003	0.005
rs2345941	A	0.55	0.015	0.002	0.000	0.005
rs13262595	A	0.44	-0.016	0.002	-0.005	0.005
rs9477970	T	0.81	-0.016	0.003	-0.013	0.006

rs2220599	C	0.63	-0.016	0.002	0.006	0.005
HbA1c						
rs12145677	G	0.70	-0.017	0.002	0.000	0.002
rs10754920	C	0.11	0.020	0.003	-0.004	0.006
rs6744254	C	0.47	-0.016	0.002	0.001	0.002
rs2068625	T	0.30	-0.016	0.002	-0.001	0.002
rs2220599	C	0.63	-0.016	0.002	0.003	0.002
rs1469249	G	0.79	0.016	0.003	0.001	0.002
rs162894	T	0.33	0.013	0.002	0.002	0.002
rs9477970	T	0.81	-0.016	0.003	-0.001	0.002
rs9372625	G	0.62	-0.018	0.002	-0.001	0.002
rs17789218	T	0.76	-0.015	0.003	0.000	0.002
rs6935828	C	0.45	-0.012	0.002	-0.005	0.002
rs3944151	A	0.28	0.014	0.002	0.002	0.002
rs2345941	A	0.55	0.015	0.002	-0.002	0.002
rs13262595	A	0.44	-0.016	0.002	-0.001	0.002
rs2734849	A	0.49	-0.014	0.002	0.000	0.002
rs1448355	C	0.38	-0.015	0.002	-0.003	0.002
rs1319734	A	0.60	0.013	0.002	0.001	0.002
rs206965	T	0.21	0.016	0.003	-0.003	0.002
rs198262	C	0.04	0.030	0.005	-0.005	0.004
rs166835	C	0.44	0.013	0.002	0.002	0.002
rs4702	G	0.44	0.012	0.002	0.000	0.002
rs6498759	T	0.31	0.013	0.002	-0.004	0.002
rs4073003	A	0.87	0.020	0.003	0.001	0.003
rs7209653	T	0.70	0.016	0.002	0.003	0.002
rs12603813	T	0.75	0.014	0.003	-0.001	0.002
rs784256	G	0.19	0.019	0.003	0.000	0.002
ISI						
rs12145677	G	0.70	-0.017	0.002	-0.100	0.084
rs6744254	C	0.47	-0.016	0.002	-0.068	0.076
rs2068625	T	0.30	-0.016	0.002	0.078	0.082
rs2220599	C	0.63	-0.016	0.002	-0.120	0.080
rs1469249	G	0.79	0.016	0.003	-0.035	0.093
rs162894	T	0.33	0.013	0.002	-0.130	0.080
rs9477970	T	0.81	-0.016	0.003	-0.130	0.110
rs9372625	G	0.62	-0.018	0.002	-0.052	0.080
rs17789218	T	0.76	-0.015	0.003	-0.006	0.084
rs6935828	C	0.45	-0.012	0.002	-0.011	0.075
rs3944151	A	0.28	0.014	0.002	-0.062	0.088
rs2345941	A	0.55	0.015	0.002	0.034	0.072
rs13262595	A	0.44	-0.016	0.002	0.034	0.079
rs2734849	A	0.49	-0.014	0.002	-0.110	0.075
rs1448355	C	0.38	-0.015	0.002	-0.098	0.081
rs1319734	A	0.60	0.013	0.002	0.009	0.077

rs206965	T	0.21	0.016	0.003	0.100	0.094
rs198262	C	0.04	0.030	0.005	0.140	0.220
rs166835	C	0.44	0.013	0.002	0.140	0.074
rs4702	G	0.44	0.012	0.002	0.025	0.087
rs6498759	T	0.31	0.013	0.002	0.054	0.078
rs4073003	A	0.87	0.020	0.003	-0.080	0.110
rs7209653	T	0.70	0.016	0.002	-0.140	0.078
rs12603813	T	0.75	0.014	0.003	-0.084	0.089
rs784256	G	0.19	0.019	0.003	-0.031	0.098
BMI						
rs10208088	C	0.42	0.013	0.002	0.002	0.002
rs10754920	C	0.11	0.020	0.003	0.007	0.003
rs113851275	G	0.89	-0.021	0.004	-0.002	0.003
rs11749912	A	0.42	0.014	0.002	0.008	0.002
rs12145677	G	0.70	-0.017	0.002	-0.011	0.002
rs12603813	T	0.75	0.014	0.003	-0.001	0.002
rs12706626	G	0.62	-0.013	0.002	-0.002	0.002
rs13262595	A	0.44	-0.016	0.002	-0.005	0.002
rs136553	C	0.62	-0.015	0.002	-0.007	0.002
rs1448355	C	0.38	-0.015	0.002	0.001	0.002
rs1469249	G	0.79	0.016	0.003	-0.010	0.002
rs162894	T	0.33	0.013	0.002	0.004	0.002
rs166835	C	0.44	0.013	0.002	0.001	0.002
rs198262	C	0.04	0.030	0.005	-0.001	0.004
rs2068625	T	0.30	-0.016	0.002	0.007	0.002
rs206965	T	0.21	0.016	0.003	0.003	0.002
rs2220599	C	0.63	-0.016	0.002	0.004	0.002
rs2345941	A	0.55	0.015	0.002	-0.002	0.002
rs2734849	A	0.49	-0.014	0.002	0.009	0.002
rs2748985	T	0.45	-0.015	0.002	-0.014	0.002
rs35933007	G	0.77	-0.015	0.003	0.008	0.002
rs3730399	A	0.93	0.025	0.004	-0.011	0.004
rs3944151	A	0.28	0.014	0.002	0.000	0.002
rs4702	G	0.44	0.012	0.002	0.005	0.002
rs4977839	G	0.58	-0.020	0.002	-0.010	0.002
rs55772938	A	0.70	-0.015	0.002	-0.009	0.002
rs6129084	A	0.36	-0.014	0.002	-0.005	0.002
rs6498759	T	0.31	0.013	0.002	-0.001	0.002
rs66643547	C	0.65	-0.015	0.002	0.002	0.002
rs6744254	C	0.47	-0.016	0.002	-0.006	0.002
rs6774533	C	0.29	-0.015	0.002	-0.012	0.002
rs6857629	G	0.76	0.015	0.003	-0.004	0.002
rs6935828	C	0.45	-0.012	0.002	-0.001	0.002
rs7020477	A	0.73	0.014	0.002	0.002	0.002
rs7209653	T	0.70	0.016	0.002	0.003	0.002

rs7288455	A	0.44	0.013	0.002	-0.001	0.002
rs73578186	C	0.68	0.015	0.002	-0.005	0.002
rs78082503	G	0.79	-0.015	0.003	-0.001	0.002
rs784256	G	0.19	0.019	0.003	-0.015	0.002
rs9372625	G	0.62	-0.018	0.002	0.015	0.002
rs9477970	T	0.81	-0.016	0.003	-0.010	0.002
WHR						
rs10208088	C	0.42	0.013	0.002	0.000	0.002
rs10754920	C	0.11	0.020	0.003	0.001	0.003
rs113851275	G	0.89	-0.021	0.004	0.011	0.003
rs11749912	A	0.42	0.014	0.002	0.003	0.002
rs12145677	G	0.70	-0.017	0.002	-0.007	0.002
rs12603813	T	0.75	0.014	0.003	-0.005	0.002
rs12706626	G	0.62	-0.013	0.002	-0.002	0.002
rs13262595	A	0.44	-0.016	0.002	-0.003	0.002
rs136553	C	0.62	-0.015	0.002	-0.003	0.002
rs1448355	C	0.38	-0.015	0.002	0.003	0.002
rs1469249	G	0.79	0.016	0.003	-0.004	0.002
rs162894	T	0.33	0.013	0.002	-0.002	0.002
rs166835	C	0.44	0.013	0.002	-0.001	0.002
rs198262	C	0.04	0.030	0.005	-0.005	0.005
rs2068625	T	0.30	-0.016	0.002	-0.001	0.002
rs206965	T	0.21	0.016	0.003	0.004	0.002
rs2220599	C	0.63	-0.016	0.002	0.004	0.002
rs2345941	A	0.55	0.015	0.002	-0.001	0.002
rs2734849	A	0.49	-0.014	0.002	0.011	0.002
rs2748985	T	0.45	-0.015	0.002	-0.009	0.002
rs35933007	G	0.77	-0.015	0.003	0.002	0.002
rs3730399	A	0.93	0.025	0.004	-0.002	0.004
rs3944151	A	0.28	0.014	0.002	0.008	0.002
rs4702	G	0.44	0.012	0.002	0.007	0.002
rs4977839	G	0.58	-0.020	0.002	-0.004	0.002
rs55772938	A	0.70	-0.015	0.002	-0.011	0.002
rs6129084	A	0.36	-0.014	0.002	-0.002	0.002
rs6498759	T	0.31	0.013	0.002	-0.003	0.002
rs66643547	C	0.65	-0.015	0.002	0.003	0.002
rs6744254	C	0.47	-0.016	0.002	-0.003	0.002
rs6774533	C	0.29	-0.015	0.002	-0.013	0.002
rs6857629	G	0.76	0.015	0.003	-0.004	0.002
rs6935828	C	0.45	-0.012	0.002	-0.001	0.002
rs7020477	A	0.73	0.014	0.002	0.000	0.002
rs7209653	T	0.70	0.016	0.002	0.001	0.002
rs7288455	A	0.44	0.013	0.002	-0.007	0.002
rs73578186	C	0.68	0.015	0.002	-0.003	0.002
rs78082503	G	0.79	-0.015	0.003	-0.003	0.002

rs784256	G	0.19	0.019	0.003	-0.013	0.002
rs9372625	G	0.62	-0.018	0.002	0.012	0.002
rs9477970	T	0.81	-0.016	0.003	-0.007	0.002
Smoking						
rs10208088	C	0.42	0.013	0.002	-0.003	0.004
rs10754920	C	0.11	0.020	0.003	0.000	0.006
rs113851275	G	0.89	-0.021	0.004	-0.002	0.005
rs11749912	A	0.42	0.014	0.002	-0.001	0.004
rs12145677	G	0.70	-0.017	0.002	0.002	0.004
rs12603813	T	0.75	0.014	0.003	0.009	0.004
rs12706626	G	0.62	-0.013	0.002	-0.008	0.004
rs13262595	A	0.44	-0.016	0.002	0.005	0.004
rs136553	C	0.62	-0.015	0.002	-0.008	0.004
rs1448355	C	0.38	-0.015	0.002	0.006	0.004
rs1469249	G	0.79	0.016	0.003	0.002	0.005
rs162894	T	0.33	0.013	0.002	-0.007	0.004
rs166835	C	0.44	0.013	0.002	0.004	0.004
rs198262	C	0.04	0.030	0.005	0.007	0.009
rs2068625	T	0.30	-0.016	0.002	-0.004	0.004
rs206965	T	0.21	0.016	0.003	0.002	0.005
rs2220599	C	0.63	-0.016	0.002	-0.004	0.004
rs2345941	A	0.55	0.015	0.002	0.003	0.004
rs2734849	A	0.49	-0.014	0.002	-0.010	0.004
rs2748985	T	0.45	-0.015	0.002	0.008	0.004
rs35933007	G	0.77	-0.015	0.003	0.006	0.005
rs3730399	A	0.93	0.025	0.004	-0.011	0.007
rs3944151	A	0.28	0.014	0.002	-0.006	0.004
rs4702	G	0.44	0.012	0.002	-0.003	0.004
rs4977839	G	0.58	-0.020	0.002	0.000	0.004
rs55772938	A	0.70	-0.015	0.002	0.002	0.004
rs6129084	A	0.36	-0.014	0.002	-0.002	0.004
rs6498759	T	0.31	0.013	0.002	-0.005	0.005
rs66643547	C	0.65	-0.015	0.002	0.001	0.004
rs6744254	C	0.47	-0.016	0.002	0.003	0.004
rs6774533	C	0.29	-0.015	0.002	0.006	0.004
rs6857629	G	0.76	0.015	0.003	-0.003	0.004
rs6935828	C	0.45	-0.012	0.002	-0.003	0.004
rs7020477	A	0.73	0.014	0.002	0.000	0.004
rs7209653	T	0.70	0.016	0.002	-0.002	0.004
rs7288455	A	0.44	0.013	0.002	0.009	0.004
rs73578186	C	0.68	0.015	0.002	-0.003	0.004
rs78082503	G	0.79	-0.015	0.003	0.001	0.005
rs784256	G	0.19	0.019	0.003	0.004	0.005
rs9372625	G	0.62	-0.018	0.002	-0.002	0.004
rs9477970	T	0.81	-0.016	0.003	0.002	0.005

Drinking						
rs10208088	C	0.42	0.013	0.002	-0.001	0.003
rs10754920	C	0.11	0.020	0.003	0.004	0.004
rs113851275	G	0.89	-0.021	0.004	0.000	0.005
rs11749912	A	0.42	0.014	0.002	0.003	0.003
rs12145677	G	0.70	-0.017	0.002	0.001	0.003
rs12603813	T	0.75	0.014	0.003	-0.004	0.003
rs12706626	G	0.62	-0.013	0.002	-0.002	0.003
rs13262595	A	0.44	-0.016	0.002	0.005	0.003
rs136553	C	0.62	-0.015	0.002	-0.005	0.003
rs1448355	C	0.38	-0.015	0.002	0.003	0.003
rs1469249	G	0.79	0.016	0.003	-0.003	0.004
rs162894	T	0.33	0.013	0.002	-0.002	0.003
rs166835	C	0.44	0.013	0.002	-0.008	0.003
rs198262	C	0.04	0.030	0.005	0.002	0.007
rs2068625	T	0.30	-0.016	0.002	-0.003	0.003
rs206965	T	0.21	0.016	0.003	0.001	0.004
rs2220599	C	0.63	-0.016	0.002	0.000	0.003
rs2345941	A	0.55	0.015	0.002	-0.004	0.003
rs2734849	A	0.49	-0.014	0.002	-0.010	0.003
rs2748985	T	0.45	-0.015	0.002	-0.006	0.003
rs35933007	G	0.77	-0.015	0.003	-0.011	0.004
rs3730399	A	0.93	0.025	0.004	0.002	0.006
rs3944151	A	0.28	0.014	0.002	-0.003	0.003
rs4702	G	0.44	0.012	0.002	-0.004	0.003
rs4977839	G	0.58	-0.020	0.002	0.001	0.003
rs55772938	A	0.70	-0.015	0.002	0.006	0.003
rs6129084	A	0.36	-0.014	0.002	0.008	0.003
rs6498759	T	0.31	0.013	0.002	0.000	0.003
rs66643547	C	0.65	-0.015	0.002	0.002	0.003
rs6744254	C	0.47	-0.016	0.002	0.001	0.003
rs6774533	C	0.29	-0.015	0.002	-0.003	0.003
rs6857629	G	0.76	0.015	0.003	-0.002	0.003
rs6935828	C	0.45	-0.012	0.002	-0.005	0.003
rs7020477	A	0.73	0.014	0.002	0.002	0.003
rs7209653	T	0.70	0.016	0.002	-0.001	0.003
rs7288455	A	0.44	0.013	0.002	-0.001	0.003
rs73578186	C	0.68	0.015	0.002	0.000	0.003
rs78082503	G	0.79	-0.015	0.003	-0.004	0.004
rs784256	G	0.19	0.019	0.003	-0.011	0.004
rs9372625	G	0.62	-0.018	0.002	0.010	0.003
rs9477970	T	0.81	-0.016	0.003	0.001	0.004
Physical activity						
rs10208088	C	0.42	0.013	0.002	-0.012	0.004
rs10754920	C	0.11	0.020	0.003	-0.011	0.007

rs113851275	G	0.89	-0.021	0.004	0.004	0.007
rs11708955	T	0.69	-0.016	0.002	0.010	0.005
rs11749912	A	0.42	0.014	0.002	-0.015	0.004
rs12145677	G	0.70	-0.017	0.002	0.003	0.005
rs12603813	T	0.75	0.014	0.003	0.002	0.005
rs12706626	G	0.62	-0.013	0.002	0.006	0.004
rs13262595	A	0.44	-0.016	0.002	0.001	0.004
rs136553	C	0.62	-0.015	0.002	0.009	0.004
rs1448355	C	0.38	-0.015	0.002	-0.003	0.004
rs1469249	G	0.79	0.016	0.003	-0.005	0.005
rs162894	T	0.33	0.013	0.002	0.006	0.005
rs166835	C	0.44	0.013	0.002	-0.003	0.004
rs198262	C	0.04	0.030	0.005	-0.013	0.010
rs2068625	T	0.30	-0.016	0.002	0.009	0.005
rs206965	T	0.21	0.016	0.003	-0.002	0.005
rs2220599	C	0.63	-0.016	0.002	0.003	0.004
rs2345941	A	0.55	0.015	0.002	-0.018	0.004
rs2734849	A	0.49	-0.014	0.002	0.005	0.004
rs2748985	T	0.45	-0.015	0.002	-0.001	0.004
rs35933007	G	0.77	-0.015	0.003	0.017	0.005
rs3730399	A	0.93	0.025	0.004	-0.009	0.009
rs3944151	A	0.28	0.014	0.002	-0.009	0.005
rs4592851	G	0.75	-0.014	0.003	0.005	0.005
rs4702	G	0.44	0.012	0.002	-0.010	0.004
rs4977839	G	0.58	-0.020	0.002	0.003	0.004
rs55772938	A	0.70	-0.015	0.002	0.000	0.005
rs6129084	A	0.36	-0.014	0.002	0.000	0.004
rs6498759	T	0.31	0.013	0.002	-0.006	0.005
rs66643547	C	0.65	-0.015	0.002	-0.006	0.004
rs6744254	C	0.47	-0.016	0.002	-0.004	0.004
rs6774533	C	0.29	-0.015	0.002	0.010	0.005
rs6857629	G	0.76	0.015	0.003	-0.007	0.005
rs6935828	C	0.45	-0.012	0.002	0.000	0.004
rs7020477	A	0.73	0.014	0.002	0.010	0.005
rs7209653	T	0.70	0.016	0.002	0.003	0.005
rs7288455	A	0.44	0.013	0.002	0.001	0.004
rs73578186	C	0.68	0.015	0.002	0.004	0.005
rs78082503	G	0.79	-0.015	0.003	-0.001	0.005
rs784256	G	0.19	0.019	0.003	0.014	0.005
rs9372625	G	0.62	-0.018	0.002	-0.004	0.004
rs9477970	T	0.81	-0.016	0.003	-0.011	0.005

Supplementary Table 21. Summary data for the genetic association between driving and outcomes.

SNP	EA	EAF	GX	GX_SE	GY	GY_SE
AS						
rs1198575	T	0.19	0.019	0.003	-0.015	0.012
rs4765541	T	0.66	0.014	0.002	0.012	0.010
rs9840902	G	0.78	-0.015	0.003	0.001	0.013
rs10186876	A	0.36	0.014	0.002	0.001	0.010
rs6012558	G	0.58	0.014	0.002	0.003	0.009
AIS						
rs1198575	T	0.19	0.019	0.003	-0.016	0.013
rs4765541	T	0.66	0.014	0.002	0.016	0.010
rs9840902	G	0.78	-0.015	0.003	0.003	0.014
rs10186876	A	0.36	0.014	0.002	0.006	0.011
rs6012558	G	0.58	0.014	0.002	0.008	0.011
CES						
rs1198575	T	0.19	0.019	0.003	-0.021	0.025
rs4765541	T	0.66	0.014	0.002	0.022	0.020
rs9840902	G	0.78	-0.015	0.003	-0.017	0.026
rs10186876	A	0.36	0.014	0.002	0.005	0.020
rs6012558	G	0.58	0.014	0.002	0.016	0.020
LAS						
rs1198575	T	0.19	0.019	0.003	-0.047	0.033
rs4765541	T	0.66	0.014	0.002	0.055	0.026
rs9840902	G	0.78	-0.015	0.003	0.024	0.034
rs10186876	A	0.36	0.014	0.002	0.041	0.027
rs6012558	G	0.58	0.014	0.002	0.021	0.025
SVS						
rs1198575	T	0.19	0.019	0.003	-0.013	0.030
rs4765541	T	0.66	0.014	0.002	0.054	0.025
rs9840902	G	0.78	-0.015	0.003	0.032	0.032
rs10186876	A	0.36	0.014	0.002	-0.042	0.024
rs6012558	G	0.58	0.014	0.002	0.004	0.024
ICH						
rs10186876	A	0.36	0.014	0.002	-0.035	0.054
rs4765541	T	0.66	0.014	0.002	-0.003	0.054
rs9840902	A	0.22	0.015	0.003	0.014	0.065
non-lobar ICH						
rs10186876	A	0.36	0.014	0.002	-0.091	0.065
rs4765541	T	0.66	0.014	0.002	0.065	0.064
rs9840902	A	0.22	0.015	0.003	0.038	0.077
lobar ICH						
rs4765541	T	0.66	0.014	0.002	-0.074	0.070
rs10186876	A	0.36	0.014	0.002	0.026	0.070
rs9840902	A	0.22	0.015	0.003	-0.055	0.087

WMH						
rs1198575	T	0.19	0.019	0.003	0.012	0.016
rs10186876	A	0.36	0.014	0.002	-0.010	0.013
rs6012558	G	0.58	0.014	0.002	0.008	0.013
rs4765541	T	0.66	0.014	0.002	0.008	0.013
AD						
rs1198575	T	0.19	0.019	0.003	0.015	0.019
rs10186876	A	0.36	0.014	0.002	-0.010	0.015
rs9840902	G	0.78	-0.015	0.003	-0.006	0.019
rs4765541	T	0.66	0.014	0.002	-0.007	0.015
rs6012558	G	0.58	0.014	0.002	0.015	0.015
PD						
rs1198575	T	0.19	0.019	0.003	-0.016	0.028
rs10186876	A	0.36	0.014	0.002	-0.013	0.023
rs4765541	T	0.66	0.014	0.002	-0.051	0.018
rs6012558	G	0.58	0.014	0.002	0.056	0.020
MS						
rs1198575	T	0.19	0.019	0.003	-0.017	0.086
rs10186876	A	0.36	0.014	0.002	-0.025	0.023
rs4765541	T	0.66	0.014	0.002	-0.010	0.098
rs6012558	G	0.58	0.014	0.002	0.010	0.052
CP						
rs6012558	G	0.58	0.014	0.002	-0.024	0.003
rs4765541	T	0.66	0.014	0.002	-0.009	0.003
rs10186876	A	0.36	0.014	0.002	-0.009	0.003
rs1198575	T	0.19	0.019	0.003	-0.005	0.004
SBP						
rs4765541	T	0.66	0.014	0.002	0.099	0.032
rs1198575	T	0.19	0.019	0.003	-0.086	0.039
rs6012558	G	0.58	0.014	0.002	0.116	0.031
rs10186876	A	0.36	0.014	0.002	-0.072	0.032
DBP						
rs4765541	T	0.66	0.014	0.002	0.027	0.018
rs1198575	T	0.19	0.019	0.003	-0.030	0.022
rs6012558	G	0.58	0.014	0.002	0.077	0.018
rs10186876	A	0.36	0.014	0.002	-0.044	0.018
PP						
rs4765541	T	0.66	0.014	0.002	0.072	0.022
rs1198575	T	0.19	0.019	0.003	-0.060	0.026
rs6012558	G	0.58	0.014	0.002	0.045	0.021
rs10186876	A	0.36	0.014	0.002	-0.023	0.021
HDL-C						
rs1198575	T	0.19	0.019	0.003	-0.010	0.007
rs6012558	G	0.58	0.014	0.002	0.003	0.004
rs9840902	G	0.78	-0.015	0.003	0.004	0.006

LDL-C						
rs1198575	T	0.19	0.019	0.003	0.016	0.007
rs6012558	G	0.58	0.014	0.002	0.000	0.004
rs9840902	G	0.78	-0.015	0.003	-0.005	0.006
TC						
rs1198575	T	0.19	0.019	0.003	0.013	0.007
rs6012558	G	0.58	0.014	0.002	0.001	0.004
rs9840902	G	0.78	-0.015	0.003	-0.004	0.006
TG						
rs1198575	T	0.19	0.019	0.003	0.014	0.006
rs6012558	G	0.58	0.014	0.002	0.004	0.003
rs9840902	G	0.78	-0.015	0.003	0.006	0.006
HbA1c						
rs1198575	T	0.19	0.019	0.003	-0.004	0.003
rs6012558	G	0.58	0.014	0.002	0.001	0.002
rs9840902	G	0.78	-0.015	0.003	-0.003	0.002
ISI						
rs1198575	T	0.19	0.019	0.003	0.031	0.099
rs6012558	G	0.58	0.014	0.002	0.024	0.078
rs9840902	G	0.78	-0.015	0.003	0.079	0.086
BMI						
rs1198575	T	0.19	0.019	0.003	0.011	0.002
rs10186876	A	0.36	0.014	0.002	-0.002	0.002
rs4765541	T	0.66	0.014	0.002	-0.011	0.002
rs6012558	G	0.58	0.014	0.002	0.005	0.002
WHR						
rs1198575	T	0.19	0.019	0.003	0.014	0.002
rs10186876	A	0.36	0.014	0.002	-0.006	0.002
rs4765541	T	0.66	0.014	0.002	0.029	0.002
rs6012558	G	0.58	0.014	0.002	0.002	0.002
Smoking						
rs1198575	T	0.19	0.019	0.003	-0.006	0.005
rs10186876	A	0.36	0.014	0.002	0.003	0.004
rs4765541	T	0.66	0.014	0.002	-0.006	0.004
rs6012558	G	0.58	0.014	0.002	-0.004	0.004
Drinking						
rs1198575	T	0.19	0.019	0.003	-0.003	0.004
rs10186876	A	0.36	0.014	0.002	-0.004	0.003
rs4765541	T	0.66	0.014	0.002	0.002	0.003
rs6012558	G	0.58	0.014	0.002	0.001	0.003
Physical activity						
rs1198575	T	0.19	0.019	0.003	0.009	0.005
rs10186876	A	0.36	0.014	0.002	0.010	0.004
rs9840902	G	0.78	-0.015	0.003	-0.014	0.005
rs4765541	T	0.66	0.014	0.002	0.004	0.005

rs6012558	G	0.58	0.014	0.002	0.007	0.004
